# Nitrogen monoxide and calix[4]pyrrolato aluminate: structural constraint enabled NO dimerization†

**DOI:** 10.1039/d4sc02378a

**Published:** 2024-06-17

**Authors:** Senta J. Kohl, Lukas M. Sigmund, Manuel Schmitt, Lutz Greb

**Affiliations:** a Anorganisch-Chemisches Institut Ruprecht-Karls-Universität Heidelberg Im Neuenheimer Feld 270 69120 Heidelberg Germany greb@uni-heidelberg.de

## Abstract

The dimerization of nitrogen monoxide (NO) is highly relevant in homo- and heterogeneous biochemical and environmental redox processes, but a broader understanding is challenged by the endergonic nature of this equilibrium. The present work describes NO-dimerization leveraged by structurally constrained aluminum and metal–ligand cooperativity at the anionic calix[4]pyrrolato aluminate(III). Quantum chemical calculations reveal the driving force for N–N bond formation, while reactivity tests shed light on subsequent redox chemistry and NO decomposition at metal surfaces. Inhibiting the dimerization pathway by saturating NO's unpaired electron with a phenyl group (nitrosobenzene) allows trapping the 1,2-adduct as a key intermediate. Elevated temperatures result in an unprecedented and high-yielding rearrangement of the calix[4]pyrrolato ligand scaffold. Kinetic and theoretical studies provide a comprehensive picture of the rearrangement mechanism and delineate systematics for ring modification of the prominent calix[4]pyrrole macrocycle.

## Abstract 2

The dimerization of nitrogen monoxide (NO) is highly relevant in biochemical and environmental redox processes. Here, it is shown how structral constraint and element-ligand cooperativity can steer this reaction.

## Graphical abstract

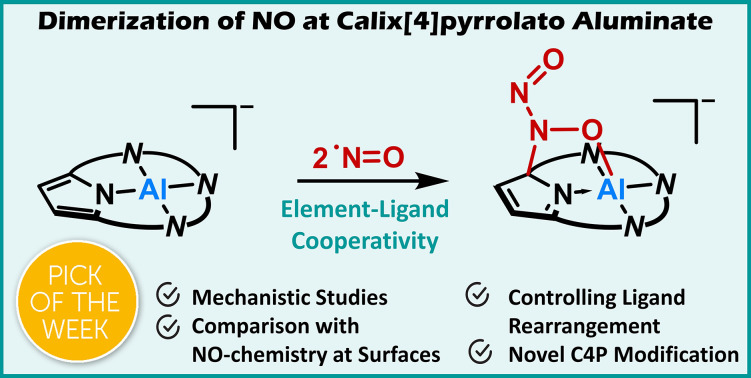

## Introduction

Nitrogen monoxide (NO) plays a pivotal role in atmospheric and physiological processes.^1–3^ Its cellular concentration is tightly regulated *via* oxidative interconversion with a nitrite (NO_2_^−^) pool.^4^ This, and essentially all other NO-related redox reactions, are sensibly controlled by the dimerization of NO to (NO)_2_.^5–14^ Several kinetic studies, such as on the environmentally relevant NO to NO_2_ transformation or the oxidation of PPh_3_, confirmed the required dimerization to be of third-order in NO.^15,16^ However, the binding energy of two NO molecules is only around 9 kJ mol^−1^ with a corresponding equilibrium constant for dimerization of < 10^−4^ M^−1^.^9,13,17–20^ Houk and coworkers showed that aromatic environments promote the formation of (NO)_2_, thus facilitating respective oxidation pathways.^13^ Still, due to the paramount bio- and geochemical relevance, further understanding of NO dimerization and its implications on NO-related reactivity is of high importance.

**Fig. 1:**
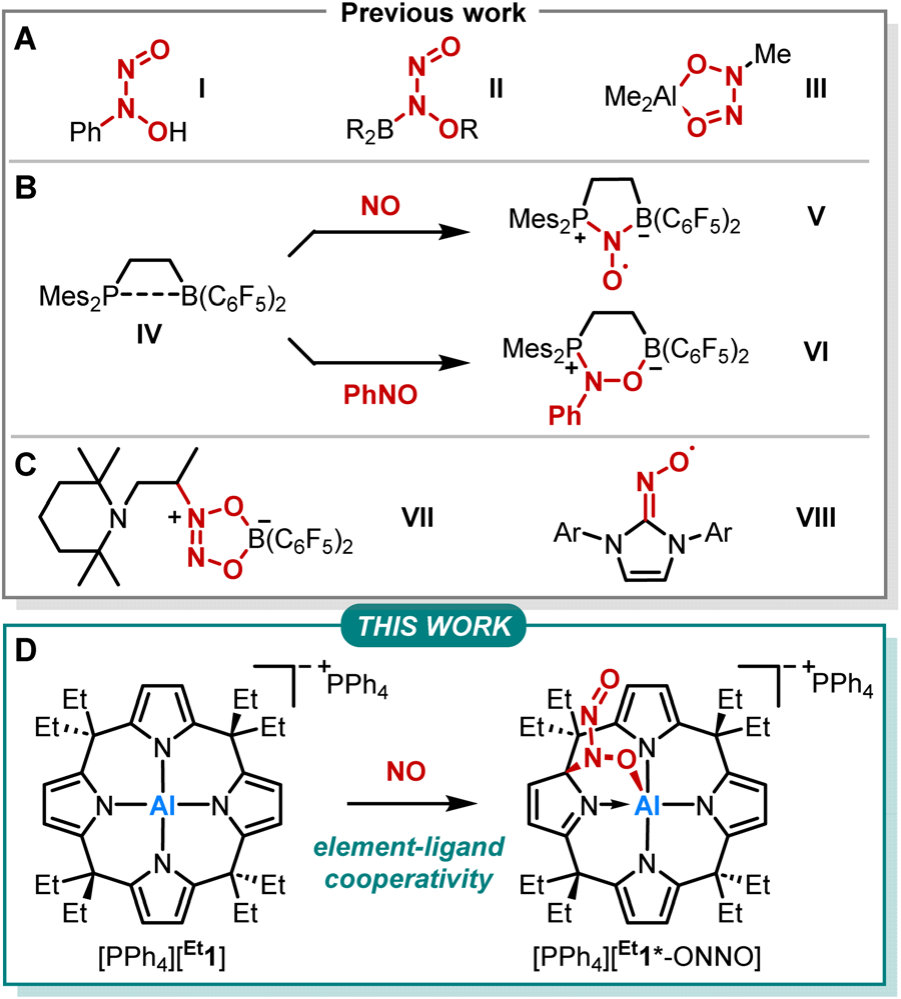
(A–C) Previously reported reactivities of NO and nitrosobenzene with main-group element compounds. (D) Reactivity of calix[4]pyrrolato aluminate ([^Et^1]^−^) with NO studied within this work. The asterisk in [^Et^1*-ONNO]^−^ indicates the dearomatization of one pyrrole unit.

The reactivity of NO with main-group element compounds is another area of active study in this field. As early as 1856, Frankland reported on NO insertion into the Zn–C bond of dialkylzinc compounds.^21–23^ Sand and Singer showed that NO interaction with Grignard reagents followed by hydrolysis provides *N*-aryl-*N*-nitrosohydroxylamines (Fig. 1A, I).^24–26^ The respective ammonium salt of the *N*-nitroso-*N*-phenylhydroxylamine anion is a versatile ligand for transition metals (cupferron).^27^ Brois described an early mechanistic study of the reaction of NO with trialkylboranes (Fig. 1A, II).^28^ Furthermore, NO inserts into the C–Al bond of trimethylaluminum, yielding an ONNO unit (Fig. 1A, III).^29,30^ Erker and coworkers extensively studied the capture of NO by various vicinal P/B frustrated Lewis pairs (FLPs) forming persistent *N*-oxyl radicals V (Fig. 1B).^31–38^ In addition, IV was treated with nitrosobenzene, which led to the formation of six-membered heterocycle VI*via* 1,2-addition (Fig. 1B).^39^ In contrast, intermolecular FLPs (*t*Bu_3_P/B(C_6_F_5_)_3_) disproportionate NO to N_2_O and the oxide of the phosphine base.^31^ The reaction of the C/B Lewis pair enamine/HB(C_6_F_5_)_2_ with NO results in the coupling of two NO molecules (Fig. 1C, VII).^40^ Lee *et al.* reported on the capture of NO with NHCs providing stable N-heterocyclic carbene nitrogen monoxide radicals ([NHC–NO]˙, Fig. 1C, VIII) exhibiting similar redox chemistry compared to transition metal nitrosyl complexes.^41,42^ Despite this plethora of examples, studies on element-ligand cooperative binding of NO different from FLP-type reactions are rare.

We previously reported on *meso*-octaalkylcalix[4]pyrrolato aluminates ([^R^1]−), which are anionic Lewis acids exhibiting a square planar anti-van't-Hoff-Le-Bel coordination of the aluminum center imposed by the macrocyclic ligand scaffold (Fig. 1D).^43–46^ The structural constraint provokes biphilic reactivity toward polarized substrates due to LUMO energy lowering and HOMO energy increase.^47,48^ Hence, aluminum-ligand cooperativity (AlLC) of calix[4]pyrrolato aluminates becomes viable for various substrates, *e.g.*, dioxygen,^49^ carbonylic,^50^ or protic substrates,^51^ showcasing the unique and diverse reactivity of [^R^1]− towards polar and apolar bonds. Aluminum-ligand cooperative bond activation towards polar bonds was also explored, *e.g.*, by Berben^52–56^ and Fedushkin.^57,58^ In the present work, we report on the reaction of calix[4]pyrrolato aluminate with another less polar substrate, namely nitrogen monoxide (Fig. 1D). In doing so, we provide insights into the critical N–N bond formation in the NO-dimer and its implications on ensuing redox chemistry. Further, we identify a novel calix[4]pyrrolato-based ring rearrangement and offer a rationale that enables generalizable ring modifications of this privileged macrocycle.

## Results and discussion

**Fig. 2:**
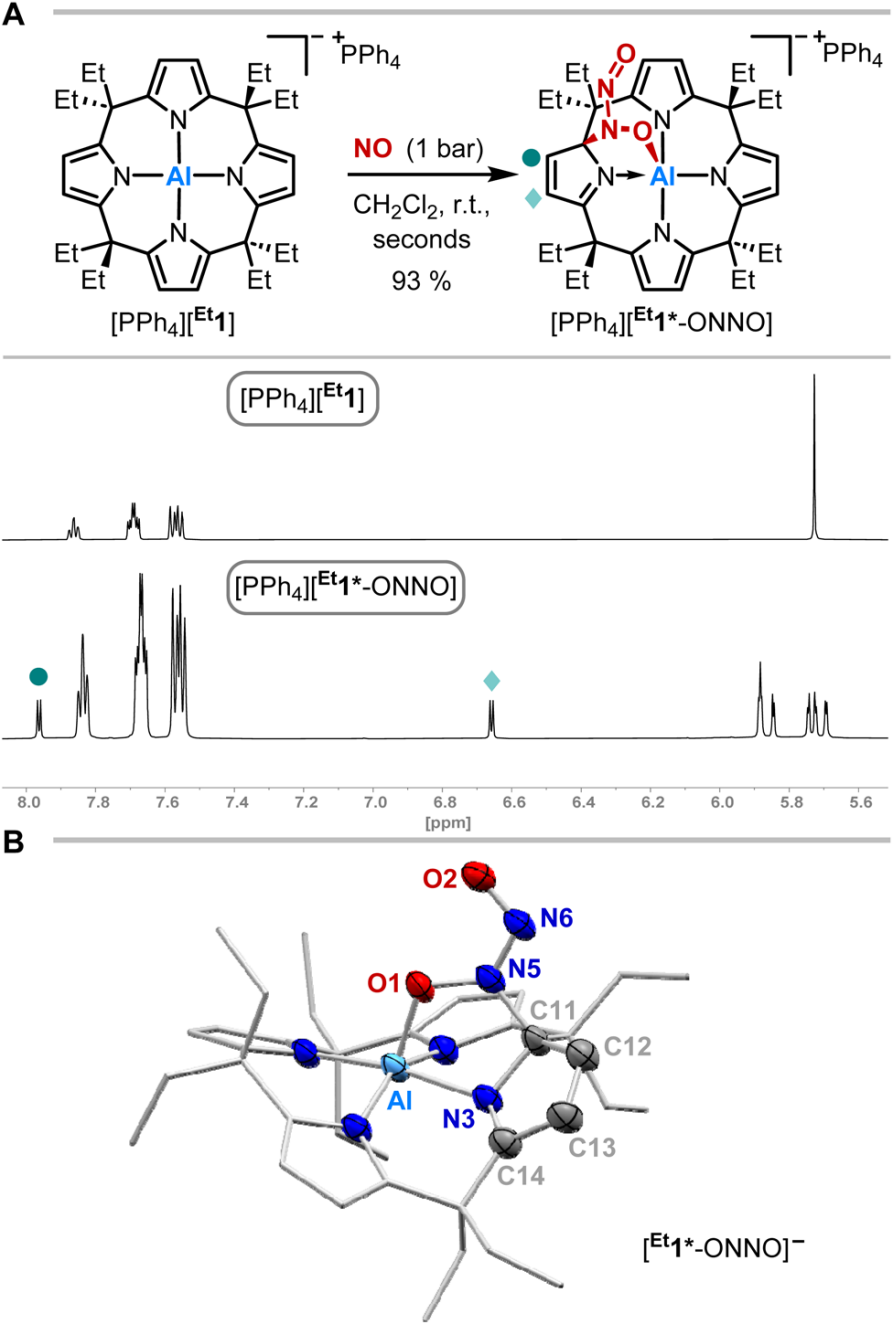
(A) Synthetic scheme for the reaction of [PPh_4_][^Et^1] with nitrogen monoxide and excerpts of the ^1^H NMR spectra (600 MHz, CD_2_Cl_2_) of [PPh_4_][^Et^1] (top) and [PPh_4_][^Et^1*-ONNO] (bottom). (B) Solid state molecular structure of [^Et^1*-ONNO]^−^ as determined by SCXRD analysis. The tetraphenylphosphonium counterion, cocrystallized solvent molecules, and all hydrogen atoms are omitted for clarity. Thermal displacement ellipsoids are displayed at the 50% probability level. Selected bond lengths [pm]: Al–O1: 185.0(2), O1–N5: 135.9(3), N5–N6: 130.1(3), N6–O2: 123.3(3), N5–C11: 149.4(4), N3–C11: 146.4(3), C11–C12: 150.6(4), C12–C13: 133.0(4), C13–C14: 147.4(4), C14–N3: 128.3(4), N3–C11: 146.4(3), Al–N3: 199.7(3). Selected bond angles [°]: Al–O1–N5: 112.52(17), O1–N5–N6: 123.1(3), N5–N6–O2: 115.7(2). Selected dihedral angle [°]: O1–N5–N6–O2: 1.8(4).

The exposure of [PPh_4_][^Et^1] to 1 bar of nitrogen monoxide in dichloromethane-d_2_ at room temperature led to a color change of the reaction mixture from colorless to dark red. *In situ*^1^H NMR spectroscopy showed a complete conversion of [PPh_4_][^Et^1] to a diamagnetic C_1_-symmetric species (Fig. 2A). The observed splitting pattern implies the dearomatization of one pyrrole unit, in analogy to observed reactivities with dioxygen,^49^ carbonylic,^50^ and protic substances.^51^

**Fig. 3:**
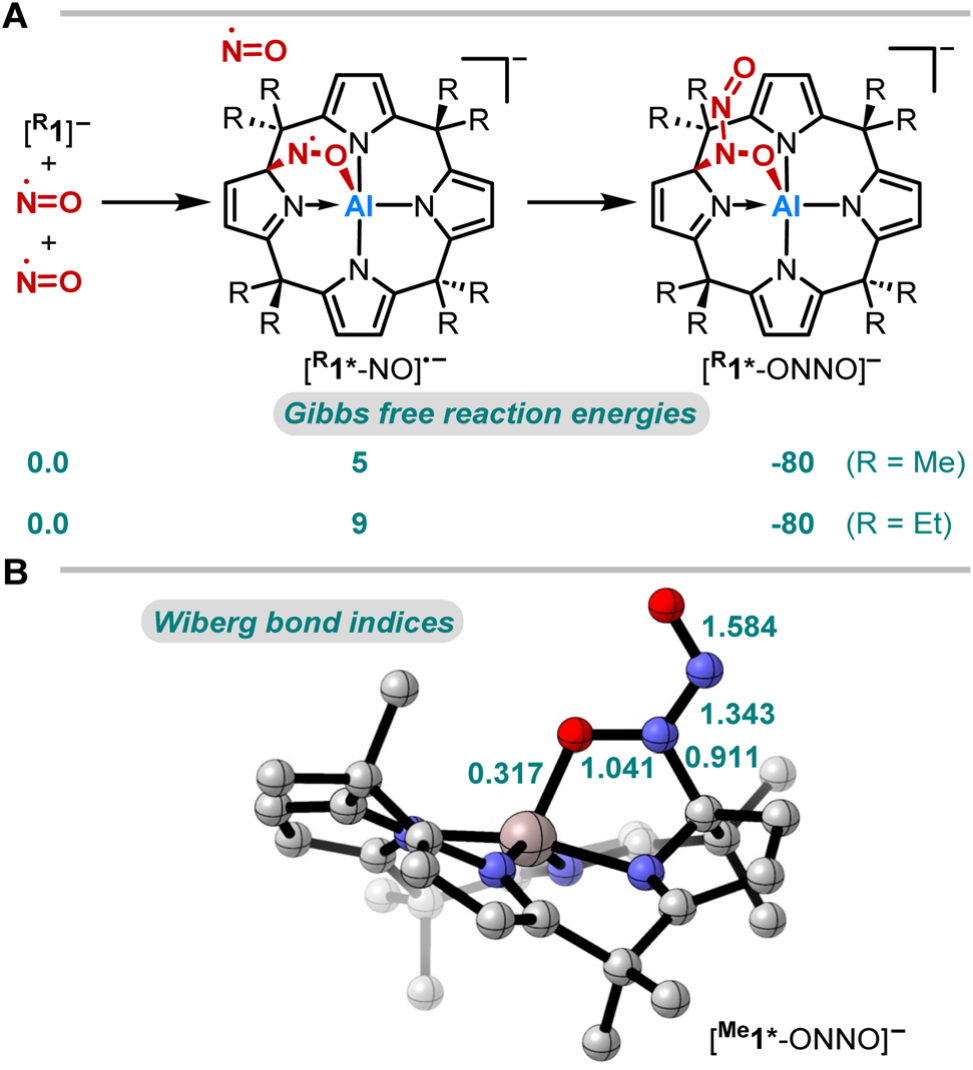
(A) Computed Gibbs free reaction energies (kJ mol^−1^) for the reaction of [^R^1] with NO at the RI-DSD-PBEB95-D3(BJ)/def2-QZVPP, COSMO-RS(CH_2_Cl_2_)//PBEh-3c level of theory and (B) Wiberg bond indices of [^Me^1*-ONNO]^−^ (PBE0/def2-TZVPP).

SCXRD analysis unveiled the dimerization of nitrogen monoxide within [PPh_4_][^Et^1*-ONNO] induced by aluminum-ligand cooperativity (Fig. 2B). Elongated C11–C12 and C13–C14 bond lengths confirm the dearomatization of one pyrrole unit. The *N*-nitrosohydroxylaminato group is bound to the aluminate in 1,2-fashion *via* a C–N and Al–O bond. The Al–O bond length (185.0(2) pm) is slightly longer than that of the addition products of dioxygen ([^Me^1*-OO]^−^: 183.11(12) pm)^49^ or CO_2_ ([^Me^1*-CO_2_]^−^: 184.8(3) pm)^50^ to [^Me^1]^−^. While the N6–O2 bond length is relatively short (123.3(3) pm), the N5–O1 distance of 135.9(3) pm in [^Et^1*-ONNO]^−^ is significantly elongated compared to the N–O bond length of the *cis*-NO-dimer (112(2) pm) observed in the solid state at low temperature.^10,11,14^ Similar distances (131.6–135.5 pm) are found in other N,O-bridging transition metal-hyponitrite complexes.^59,60^ The N5–N6 distance (130.1(3) pm) is significantly shortened compared to the *cis*-NO dimer (218(6) pm),^10,11,14^ but longer than that of a typical N=N double bond (*ca.* 124 pm),^61^ or N–N distances (123.7–128.2 pm)^62,63^ of O,O-bridging *N-*organo-*N*-nitroso-hydroxylaminato moieties in transition metal complexes. The Wiberg bond index of the N–N bond was calculated to 1.343, indicating N–N double bond character (Fig. 3B). The *N*-nitrosohydroxylaminato group has a coplanar structure with an O1–N5–N6–O2 dihedral angle of 1.8(4)°. Decomposition of [PPh_4_][^Et^1*-ONNO] was observed upon heating at 60 °C, which may be due to the release of one nitrogen monoxide. This is in line with the analysis of the reaction mixture *via* ESI(−) mass spectrometry (measured *m/z* = 593.3804; calculated *m/z* [^Et^1*-NO]˙^−^: 593.3679).

The thermodynamics of the addition reaction of NO to [^R^1]^−^ were studied using DFT calculations^64–66^ (RI-DSD-PBEB95-D3(BJ)/def2-QZVPP, COSMO-RS(CH_2_Cl_2_)//PBEh-3c, Fig. 3A). The 1,2-addition of one NO molecule by aluminum-ligand cooperativity provides [^R^1*-NO]˙^−^. This step is slightly endergonic (5 and 9 kJ mol^−1^) for the methyl and the ethyl-substituted ligand system. Upon NO dimerization, the process turns from endergonic to exergonic (*D*_R_*G* = −80 kJ mol^−1^ for both derivatives). The identified transition state energies of both steps agree with the swift reactivity at room temperature (Fig. S24 and S26†). The application of other computational methods led to consistent values. Binding modes other than the 1,2-adduct ([^R^1*-NO]˙^−^) were considered but were found to be higher in energy (Fig. S25†). Analogous calculations were conducted to confirm the critical role of structural constraint on the reaction of NO with the VSEPR-compliant [Al(pyrrolato)_4_]^−^ (Fig. S27†). A substantially more endergonic profile highlights that the calix[4]pyrrole ligand scaffold is key for the observed reactivity towards NO.

Aiming to understand the driving force of N–N bond formation, the quantum chemical data was compared with the additive-free NO dimerization process. MO theory ascribes nitrogen monoxide's unpaired electron to a π* anti-bonding orbital, which is energetically degenerate with another empty π* orbital. Bare NO can only form a loosely bound dimer. The most stable configuration was shown to be of *s-cis* configuration with a long N–N bond (218 pm in the solid state)^10,11^ and a dissociation energy of below 9 kJ mol^−1^.^18–20^ Due to the long N–N bond, the description of the electronic structure of the (NO)_2_ system requires multiconfigurational/multireference approaches.^67^ Substantial contributions of configurations with the 
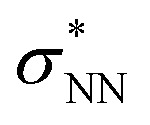
 orbital populated (*σ*_NN_ → 
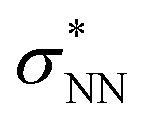
 double excitation) rationalize the low stability of (NO)_2_.^68^ In the case of [^R^1*-NO]˙^−^, the electronic structure of the bound NO is significantly altered compared to free nitrogen monoxide. Charge transfer is reflected in the elongated N–O bond length of [^Me^1*-NO]˙^−^ (131.46 pm) compared to free NO (114.02 pm). The N–O π bond is broken and the population of π*-type orbitals primarily located at the NO fragment is larger (see Fig. S24 in the ESI† for isodensity surface plots). Moreover, the degeneracy of the π* orbitals is lost and the unpaired electron is considerably increased in energy (SOMO: −7.3 eV in the free NO *vs.* −6.1 eV in [^Me^1*-NO]˙^−^, calculated at PBEh-3c, SMD(CH_2_Cl_2_) level of theory). The natural charge of the NO fragment in [^Me^1*-NO]˙^−^ was calculated to −0.71 e. The natural spin density is entirely located at the NO fragment. These electronic changes drive the formation of the N–N bond to yield [^R^1*-ONNO]^−^. Compared with the free NO dimer, the natural population of the 
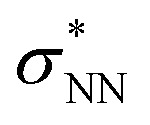
 orbital is substantially reduced in [^Me^1*-ONNO]^−^ (0.101 *versus* 0.487 e^69^). Interestingly, previous research on NO dimerization on silver or copper surfaces identified a related metal-to-NO charge transfer into NO π* orbitals as crucial for N–N bond formation.^70^ In the present case, the pyrrolato-unit serves as electron donor instead of the metal surface's Fermi electrons. But how does this specific form of covalent NO dimerization influence the reactivity?

Knowing about the relevance of the NO-dimer for oxidative transformations (see introduction), we were interested in the impact on ensuing redox chemistry. Thus, we compared the oxidation rates of P(*para*-F-C_6_H_4_)_3_ with NO gas as the oxidant in the presence and the absence of [PPh_4_][^Et^1]. Without [PPh_4_][^Et^1], the reaction is happening smoothly at ambient temperature (Section S3 in the ESI†). In the presence of 10 mol% of [PPh_4_][^Et^1], the oxidation reaction was inhibited significantly. It suggests that in the case of excessive charge transfer onto the NO unit, as described above, redox chemistry of this pseudo-dimer is blocked. Accordingly, the present system may be considered a dysfunctional model of metal surfaces with detrimentally high Fermi energy that do not enable NO decomposition. Heating of stoichiometric amounts of isolated [PPh_4_][^Et^1*-ONNO] with P(*para*-F-C_6_H_4_)_3_ did not lead to the transformation to the respective phosphine oxide. Apparently, the NO release from [PPh_4_][^Et^1*-ONNO] as observed by mass spectrometry is not sufficient to deliver enough NO for dimerization in its free form, giving further proof of the required dimerization.

**Fig. 4:**
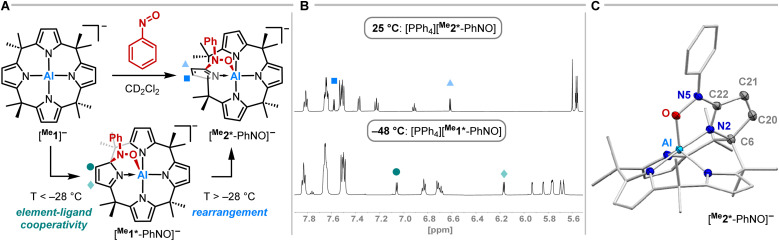
(A) Synthetic scheme for the reaction of [PPh_4_][^Me^1] with nitrosobenzene in dependence of the temperature. The tetraphenylphosphonium counterion is omitted for clarity. (B) ^1^H NMR spectra (600 MHz, CD_2_Cl_2_) of the aromatic section of [PPh_4_][^Me^1*-PhNO] (bottom) and [PPh_4_][^Me^2*-PhNO] (top). (C) Solid state molecular structure of [^Me^2*-PhNO]^−^ as determined by SCXRD analysis. The tetraphenylphosphonium counterion and all hydrogen atoms are omitted for clarity. Thermal displacement ellipsoids are displayed at the 50% probability level. Selected bond lengths [pm]: Al–O: 188.40(7), O–N5: 140.47(10), N5–C22: 135.28(12), C21–C22: 147.48(13), C20–C21: 134.27(14), C6–C20: 152.01(13), C6–N2: 143.94(12), N2–C22: 129.34(11). Selected bond angles [°]: Al–O–N5: 113.77(5), O–N5–C22: 112.88(7).

Having studied the impact of NO dimerization on follow reactivity, the idea arose to understand the mechanism further. Thus, [PPh_4_][^Me^1] was subjected to nitrosobenzene, which can be considered as a NO congener with the radical position blocked by a phenyl group (Fig. 4A). ^1^H NMR spectroscopy revealed the selective formation of a pristine high-symmetry species, featuring only five signals for the pyrrolic b-protons (eight were observed for [^Me^1*-ONNO]^−^) and four signals for the methyl groups (Fig. 4B, top). Two downfield-shifted signals of pyrrolic b-protons indicated a dearomatized pyrrole ring.

SCXRD studies unveiled an unprecedented rearrangement of the calix[4]pyrrolato ligand framework toward [^Me^2*-PhNO]^−^, wherein the dearomatized pyrrole unit is positioned perpendicular to the calix[4]pyrrolato plane (Fig. 4C). The central aluminum adopts almost ideal trigonal bipyramidal N_4_O-coordination. Substrate binding occurs *via* the formation of an Al–O (188.40(7) pm) and a C–N bond (135.28(12) pm). Compared to [PPh_4_][^Et^1*-ONNO], the Al–O distance is slightly elongated ([PPh_4_][^Et^1*-ONNO]: 185.0(2) pm), while the C–N bond length is significantly contracted ([PPh_4_][^Et^1*-ONNO]: 149.4(4) pm). The dearomatized state of the pyrrole ring can be verified by shortened N2–C22 and C20–C21 distances. [PPh_4_][^Me^2*-PhNO] exhibits Al–N distances ranging from 190 to 192 pm for the aromatic pyrrole rings but a shortened dative Al–N bond (188.13(8) pm) for the dearomatized pyrrole.

**Fig. 5:**
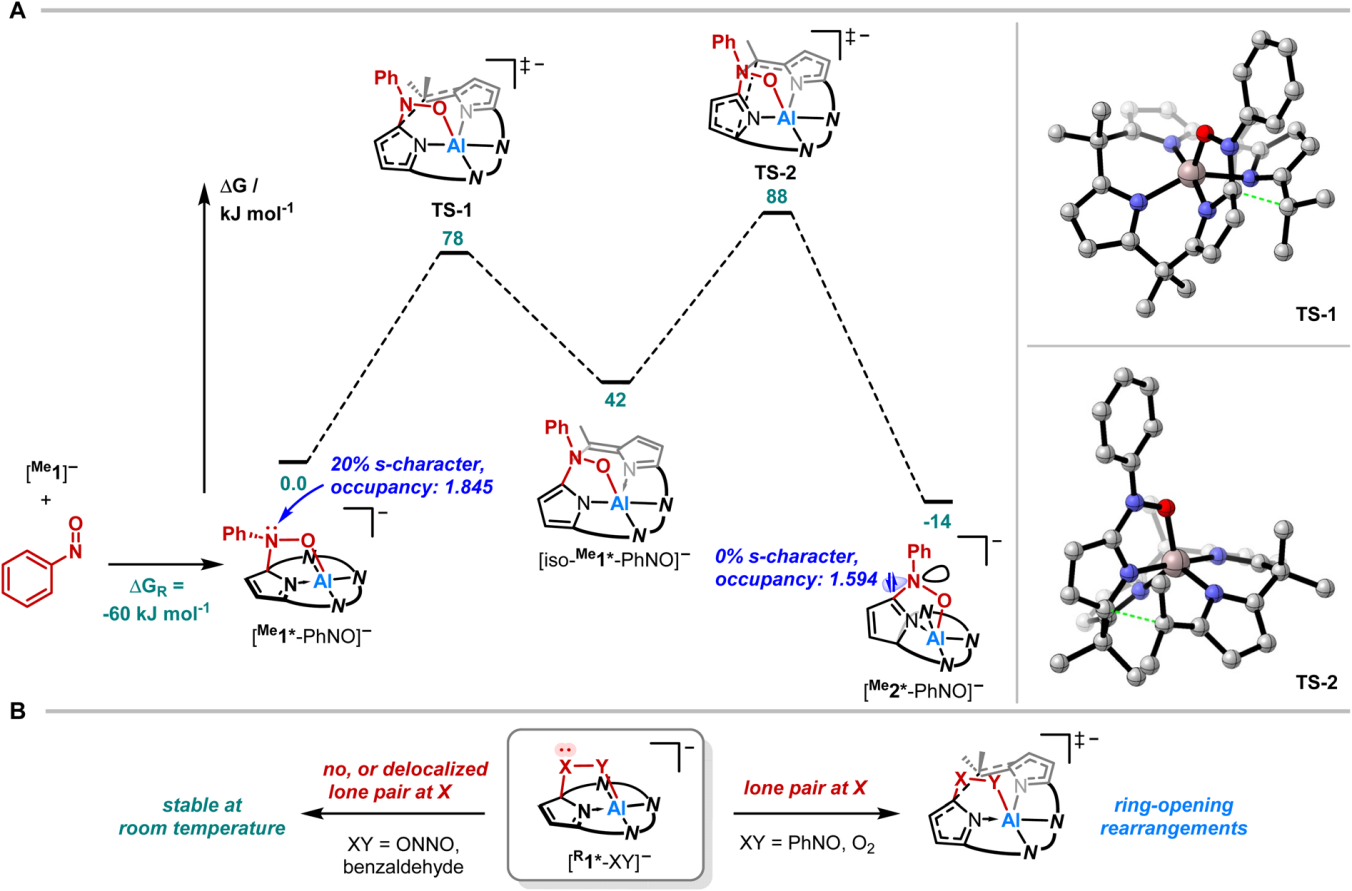
(A) Computed reaction profile for the rearrangement of [PPh_4_][^Me^1*-PhNO] at the RI-DSD-PBEB95-D3(BJ)/def2-QZVPP, COSMO-RS(CH_2_Cl_2_)//PBEh-3c level of theory and the respective transition structures. (B) Schematic representation on the influence of the presence of a lone-pair at the C-bound X on the stability of 1,2-adducts generalized as [^R^1*-XY]^−^.

Quantum chemical calculations were conducted to elucidate the formation mechanism of [PPh_4_][^Me^2*-PhNO] (Fig. 5A and B). We assumed [^Me^1*-PhNO]^−^ as the first key intermediate, which forms with *D*_R_*G* = −60 kJ mol^−1^. C–C bond cleavage results in the formation of [*iso*-^Me^1*-PhNO]^−^, and subsequent C–C bond formation leads to the even more exergonic [^Me^2*-PhNO]^−^ (*D*_R_*G* = −14 kJ mol^−1^ relative to [^Me^1*-PhNO]^−^). Both steps feature energetic barriers that align with the feasibility of the rearrangement at room temperature.

To experimentally corroborate the 1,2-adduct [^Me^1*-PhNO]^−^ as an intermediate, the reaction was monitored by ^1^H VT-NMR spectroscopy. Equimolar amounts of [PPh_4_][^Me^1] and nitrosobenzene were separately dissolved in CD_2_Cl_2_, combined at −78 °C, and immediately analyzed at low temperature. Characteristic signals of the 1,2-adduct [PPh_4_][^Me^1*-PhNO] were observed at −28 °C (Fig. 4B, lower spectrum). Upon warming to −13 °C, the signals for [PPh_4_][^Me^2*-PhNO] appeared. The kinetics of the rearrangement process were examined *via* time-resolved VT-NMR measurements. Based on the obtained spectra, the rate constant was determined to *k*_obs_ = 1.35 × 10^−4^ s^−1^ at −13 °C, with the corresponding Gibbs free activation energy for the rearrangement as 
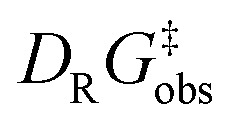
 = 83 kJ mol^−1^. This value is in line with the DFT-computed Gibbs free activation energy (*D*_R_*G*^‡^ = 88 kJ mol^−1^ at 25 °C, Fig. 5).

Finally, we aimed to derive a rationale for the ring-rearrangement reaction that is occurring in [^Me^1*-PhNO]^−^, but which is absent in [PPh_4_][^Et^1*-ONNO]. NBO calculations (PBE0/def2-TZVPP) showed that the rearrangement from [^Me^1*-PhNO]^−^ to [^Me^2*-PhNO]^−^ leads to rehybridization of the N-lone pair of the nitrogen atom of the added nitrobenzene from 20 to 0% s-character. The ensuing extension of the p-system, illustrated by the decreased natural population of the respective N-lone pair and the experimentally observed C22–N5 bond shortening, stabilizes [^Me^2*-PhNO]^−^ and can be associated with the driving force of the rearrangement. This situation is different in the case of [^Me^1*-ONNO]^−^, where the respective N-lone pair is strongly delocalized within the *N-*nitrosohydroxylaminato unit (coplanar ONNO unit with N=N double bond character). Indeed, the rearrangement of [^Me^1*-ONNO]^−^ to [^Me^2*-ONNO]^−^ is endergonic by 23 kJ mol^−1^ (see ESI, Fig. S26†).

This comparison allows even broader statements on the tendency of calix[4]pyrrole adducts toward rearrangements. In the corresponding 1,2-adduct to benzaldehyde, no rearrangement occurs.^71^ Due to the lack of stabilization in a rearranged product by p-delocalization (Fig. 5B, left), the analogous reaction product is endergonic by *D*_R_*G* = 34 kJ mol^−1^ relative to the experimentally observed 1,2-addition product (see ESI, Fig. S25†). It also explains why the presence of the lone-pair at the C-bound oxygen in the aluminum peroxide product [^Me^1*-OO]^−^ after O_2_ binding (X/Y = O, Fig. 5B, right) induces the experimentally observed rearrangement.^49^ Hence, these insights provide a design concept for reactions toward ring-modified derivatives of this prominent macrocycle in coordination and supramolecular chemistry.^72–75^

## Conclusions

The present study describes the first dimerizing capture of nitrogen monoxide by metal–ligand cooperativity and a novel calix[4]pyrrole rearrangement upon reacting the aluminate with nitrosobenzene. Hence, the results impact two broader fields: general NO-redox and calix[4]pyrrolato-related transformations.

(1) The implications of the NO dimerization on the oxidation capability were gauged by reacting with a phosphine. Minor amounts of spontaneously formed (NO)_2_ in solution (<10^−4^ M^−1^) exhibit a much higher activity compared to the stoichiometric but covalent (NO)_2_ dimer in [PPh_4_][^Et^1*-ONNO]. Computations reveal ligand-to-NO charge transfer to drive the dimerization. Hence, this comparison indicates that pronounced electronic perturbations of (NO)_2_ by charge transfer are detrimental to ensuing redox reactivity. Contextualized with NO dimerization driven by absorption on metal surfaces in combustion engine car-exhaust catalysts, these insights might guide the impact of Fermi electron levels.^76–78^

(2) The binding of [PPh_4_][^Me^1] with nitrosobenzene induces a novel calix[4]pyrrole-based rearrangement, which gives a pentadentate N_4_O scaffold in two steps with 70% yield starting from octamethylcalix[4]pyrrole. The product represents the first example of a calix[4]pyrrole with one orthogonal pyrrole ring, an attractive rigid ligand for other metals. In its protonated form, it is a promising shape-persistent anion acceptor and an alternative for strapped or topographically non-planar calixpyrrole analogs.^72–75^ The tendency of rearrangement is explained based on the availability of electron lone pairs at the carbon-bound atom, providing a blueprint for future ring-modification reactions of this macrocycle.

## Data availability

Crystallographic data have been deposited at the CCDC: 2342027 and 2342028. Further data supporting this study are available in the ESI.†

## Author contributions

L. M. S. and L. G. devised the project and designed the experiments. S. J. K. and L. M. S. performed the experimental work. L. M. S. and S. J. K. carried out the quantum chemical simulations. M. S. finalized the SCXRD data. S. J. K. wrote the initial manuscript. All authors contributed to the finalization of the manuscript, and all listed authors agreed to the submitted content.

## Conflicts of interest

There are no conflicts to declare.

## Supplementary Material

SC-015-D4SC02378A-s001

SC-015-D4SC02378A-s002
