# Supplementary material for: Nitrogen monoxide and calix[4]pyrrolato aluminate: structural constraint enabled NO dimerization
Source: Chem Sci. 2024 Jun 17;15(28):10803–9. doi: 10.1039/d4sc02378a (PMC11253113; doi:10.1039/d4sc02378a)
Supplement: SC-015-D4SC02378A-s001 [file SC-015-D4SC02378A-s001.pdf]

Electronic Supplementary Material (ESI) for Chemical Science.
This journal is © The Royal Society of Chemistry 2024

Supporting information for

Nitrogen Monoxide and Calix[4]pyrrolato Aluminate: Structural Constraint Enabled NO Dimerization

Senta J. Kohl†, Lukas M. Sigmund†, Manuel Schmitt and Lutz Greb

† The authors contributed equally.

Author Address: Anorganisch-Chemisches Institut, Ruprecht Karls-Universität Heidelberg, Heidelberg 69120, Germany.

Table of Contents

1 Materials and methods 2

2 Synthesis 3

2.1 *meso*-octamethylcalix[4]pyrrole 3

2.2 *meso*-octaethylcalix[4]pyrrole 4

2.3 $[PPh_4]$[^{Me}**1**] 4

2.4 $[PPh_4]$[^{Et}**1**] 5

2.5 $[PPh_4]$[^{Et}**1***-ONNO] 6

2.6 $[PPh_4]$[^{Me}**2***-PhNO] 7

2.7 $[PPh_4]$[^{Me}**1***-PhNO] 8

3 Reactivity tests with phosphanes 9

4 Decomposition of $[PPh_4]$[^{Et}1*-ONNO] 11

5 Determination of the Rearrangement Barrier 11

6 X-ray crystallography 13

7 NMR spectra 15

8 FT-ATR-IR spectra 22

9 UV-Vis absorption spectra 23

10 Quantum chemical calculations 24

11 Reaction mechanism of the NO dimerization at $[^{R}1]^{-}$ 25

12 Comparison of the reactions of $[^{Me}1]^{-}$ and [Al(pyrrolato)$_4$] with NO..........27

13 Further quantum chemical data..........28

14 xyz coordinates..........30

15 References..........65

1 Materials and methods

All manipulations, apart from the synthesis of the calix[4]pyrrole ligands, were carried out under a dry nitrogen or argon atmosphere employing standard Schlenk line techniques. Solvents were degassed prior to use with four freeze-pump-thaw cycles and were stored over activated molecular sieve (3 or 4 Å, respectively) under a dry argon or nitrogen atmosphere. Air sensitive compounds were handled and stored in a glove box filled with dry nitrogen gas (MBraun LABmaster dp, MB-20-G, Sylatech Y05G). All glassware, syringes, magnetic stirring bars, and needles were thoroughly dried and/or flushed with argon or nitrogen prior to use. Reactions on NMR scale were done in dry J. Young NMR tubes. All reagents and solvents used within this research project were purchased from commercial sources. Nitrogen monoxide gas was used from Air Liquide and was used without further purification. The group of Prof. Dr. Lutz Gade is acknowledged for providing access to NO gas.

Nuclear magnetic resonance (NMR) spectra were recorded with a Bruker Avance II 400 or Bruker Avance III 600 spectrometers. The ^{1}H and $^{13}C\{^{1}H\}$ NMR chemical shifts δ are given in parts per million (ppm) relative to tetramethylsilane as an external standard. ^{15}N NMR chemical shifts δ [ppm] are reported to the IUPAC nitromethane standard. The ^{1}H and ^{13}C NMR spectra are calibrated using the signal of $CHDCl_2$ (^{1}H: 5.32 ppm, ^{13}C: 53.84 ppm).[1] The ^{15}N NMR chemical shifts relative to the NH_3 system were extracted from $^{1}H,^{15}N$ HMBC NMR spectra without manual calibration. The ^{15}N NMR chemical shifts were converted to the nitromethane reference system by subtracting 380.23 ppm. Observed multiplicities are described by employing common abbreviations (s = singlet, br s = broad singlet, d = doublet, t = triplet, q = quartet, m = multiplet). Scalar spin-spin coupling constant [Hz] are described as $^{X}J_{AB}$ (X = number of chemical bonds between coupled nuclei; A, B = coupled nuclei). For peak assignment of novel compounds additional two-dimensional NMR spectra were recorded such as $^{1}H,^{1}H$ COSY, $^{1}H,^{13}C$ HSQC and $^{1}H,^{13}C$ HMBC and broadband ^{1}H decoupled ^{13}C-DEPT135. The type of carbon atom is described by the number of bound hydrogen atoms: CH_3, CH_2, CH, C_q. The protons of the aromatic pyrrole rings of the calix[4]pyrrolato ligands are denoted *β-H* and the directly attached carbon atoms *β-C*. The quaternary carbon atoms of the aromatic pyrrole rings in the ligand are described as *C_q-pyrrole*. The atoms of the ligands methyl and methylene groups are denoted *α-Me* and *α-methylene*.

The quaternary carbon atoms to which they are bound are denoted *α-C*. NMR spectra were processed and plotted with MestReNova v15.0.1-36756.

Electrospray ionization mass spectrometry (ESI-MS) was done with a Bruker ApexQe. Mass spectra were measured at the Institute of Organic Chemistry of Heidelberg University.

UV-Vis absorption spectra were recorded with an Agilent Cary 5000 device using a quartz cuvette equipped with a J. Young valve. Measurements were carried out under a dry nitrogen atmosphere at room temperature. Reported are the maximum wavelengths λ_{max} [nm] of the respective absorption bands.

Fourier-transform attenuated total reflection infrared spectra (FT-ATR-IR) were recorded on an Agilent Cary 630 instrument with solid material inside a glove box filled with dry nitrogen gas at room temperature. Intensities are denoted using common abbreviations (s = strong, m = medium, w = weak).

Elemental analysis (EA) to determinate the C-, H- and N-content was carried out by the microanalysis laboratory of Heidelberg University using a vario MICRO cube of Elementar Analysensysteme GmbH.

For **data handling and plotting** Origin 2024 was used.

2 Synthesis

The ligands *meso*-octamethylcalix[4]pyrrole and *meso*-octaethylcalix[4]pyrrole and the respective aluminates ([PPh_4][^{Me}**1**] and [PPh_4][^{Et}**1**]) were synthesized according to slightly modified literature procedures. The respective publications are cited, respectively. Their purity was verified by NMR spectroscopy prior to usage. Novel compounds were characterized relative to reported structures to the best of our knowledge.

2.1 *meso*-octamethylcalix[4]pyrrole

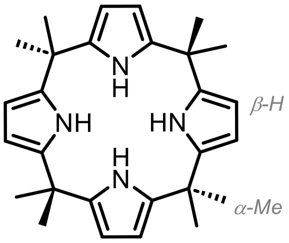

Procedure.[2, 3] In a 500 mL round-bottom flask, equipped with a reflux condenser, pyrrole (15 g, 15.51 mL, 223.58 mmol, 1.00 eq.) and acetone (13.00 g, 16.56 mL, 223.56 mmol, 1.00 eq.) were

dissolved in methanol (160 mL). Catalytic amounts of methanesulfonic acid (8 drops) were added to the solution. The reaction mixture was stirred for 4 h at 60 °C. A precipitate formed. Afterwards the solvent was removed under reduced pressure. The obtained brown solid was further purified by flash column chromatography on silica gel (DCM/PE, 2:1, R_f = 0.58) to yield *meso*-octamethylcalix[4]pyrrole as a light brown solid (15.31 g, 35.72 mmol, 64 % yield).

^{1}H NMR (600 MHz, CD_2Cl_2, 298 K), δ_{1H} [ppm] = **7.03** (br s, 4H, *N-H*), **5.88** (d, $^3J_{HH}$ = 2.8 Hz, 8H, *β-H*), **1.49** (s, 24H, *α-Me*).

2.2 *meso*-octaethylcalix[4]pyrrole

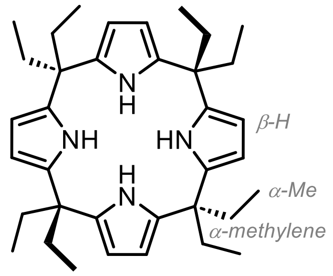

Procedure.[2, 3] In a 500 mL round-bottom flask equipped with a reflux condenser, pyrrole (20.00 g, 20.62 mL, 298.10 mmol, 1.00 eq.) and 3-pentanone (25.68 g, 31.51 mL, 298.10 mmol, 1.00 eq.) were dissolved in ethanol (100 mL). Catalytic amounts of trifluoromethanesulfonic acid (8 drops) were added to the solution. The reaction mixture was stirred for 4 h at 90 °C. Afterwards the reaction mixture was cooled down to room temperature and stored at -40 °C for 16 h. The crude product was separated by filtration and washed with ethanol (3 x 20 mL). The obtained colorless solid was dried under reduced pressure. Further purification was achieved by flash column chromatography on silica gel (DCM/PE, 40:60, R_f = 0.59) to yield *meso*-octaethylcalix[4]pyrrole as a colorless solid (10.81 g, 19.99 mmol, 27 % yield).

^{1}H NMR (200 MHz, $CDCl_3$, 295 K), δ_{1H} [ppm] = **6.97** (br s, 4H, N-H), **5.90** (d, $^3J_{HH}$ = 2.7 Hz, 8H, β-H), **1.77** (br s, 16H, *α-methylene*), **0.59** (t, $^3J_{HH}$ = 7.4 Hz, 24H, *α-Me*).

2.3 [PPh_4][^{Me}**1**]

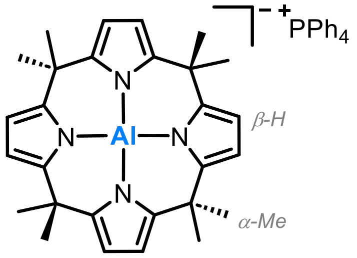

Procedure.[4] In a 500 mL Schlenk tube, *meso*-octamethylcalix[4]pyrrole (3.00 g, 7.00 mmol, 1.00 eq.) and lithium aluminum hydride (610.98 mg, 16.10 mmol 2.30 eq.) were dissolved in 1,2-dimethoxyethane (25 mL). An overpressure valve was added, and the reaction mixture was refluxed at 95 °C for 48 h. After the solvent was removed under reduced pressure, the remaining solid was dissolved in dichloromethane (40 mL). Tetraphenylphosphonium chloride (2.62 g, 7.00 mmol, 1.00 eq.) was added in one portion. The reaction mixture was stirred for 1 h at room temperature. Afterwards the formed precipitate was separated by filtration. Precipitation with *n*-pentane (60 mL) yielded the product as a pale-yellow solid (4,27 g, 5.40 mmol, 77 % yield). The ^{1}H NMR spectrum revealed the presence of approximately an equimolar amount of dichloromethane.

^{1}H NMR (400 MHz, CD_2Cl_2, 296 K), δ_{1H} [ppm] = **7.90-7.83** (m, 4H, *PPh_4^+*), **7.72-7.66** (m, 8H, *PPh_4^+*), **7.60-7.53** (m, 8H, *PPh_4^+*), **5.75** (s, 8H, *β-H*), **1.64** (s, 12H, *α-Me*), **1.47** (s, 12H, *α-Me*).

2.4 [PPh4][^{Et}**1**]

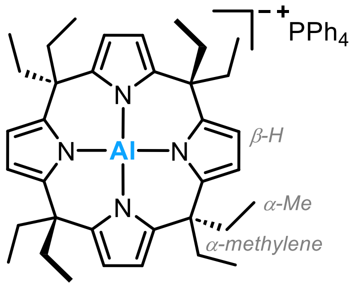

Procedure.[4, 5] In a Schlenk tube, *meso*-octaethylcalix[4]pyrrole (3.00 g, 5.55 mmol, 1.00 eq.) and lithium aluminum hydride (484.21 mg, 12.76 mmol, 2.30 eq.) were dissolved in 1,2-dimethoxy-ethane (20 mL). An overpressure valve was added and afterwards the reaction mixture was refluxed at 95 °C for 48 h. After the solvent was removed under reduced pressure, the reaction mixture was dissolved in dichloromethane (20 mL). Tetraphenylphosphonium chloride (2.08 g, 5.55 mmol, 1.00 eq.) was added to the solution. The reaction mixture was stirred for 2 h at room temperature. Afterwards the formed precipitate was separated by filtration. A pale-yellow solid was isolated from its dichloromethane solution by precipitation with *n*-pentane (30 mL). Drying under reduced pressure yielded the product as a pale-yellow solid (3.70 g, 4.10 mmol, 74 % yield).

^{1}H NMR (200 MHz, CD_2Cl_2, 300 K), δ_{1H} [ppm] = **7.93-7.81** (m, 4H, *PPh_4^+*), **7.75-7.49** (m, 16H, *PPh_4^+*), **5.73** (s, 8H, *β-H*), **2.03** (q, $^3J_{HH}$ = 7.3 Hz, 8H, *α-methylene*), **1.68** (q, $^3J_{HH}$ = 7.3 Hz, 8H, *α-methylene*), **1.07** (t, $^3J_{HH}$ = 7.2 Hz, 12H, *α-Me*), **0.53** (t, $^3J_{HH}$ = 7.3 Hz, 12H, *α-Me*).

2.5 [PPh_4][^{Et}**1***-ONNO]

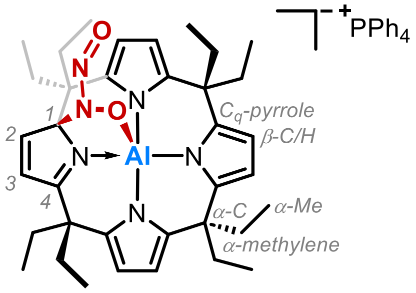

Procedure. In a Schlenk tube, [PPh_4][^{Et}**1**] (150.00 mg, 166.08 µmol, 1.00 eq.) was dissolved in DCM. The sample was pressurized multiple times with 1 bar of nitrogen monoxide gas at room temperature. The color of the reaction mixture changed within seconds from colorless to a dark red-brown. The solvent was removed under reduced pressure to yield the product as a brown solid (148.30 mg, 153.97 µmol, 93 %).

Single crystals suitable for X-ray diffraction were grown by vapor diffusion of diethyl ether into a THF solution at -40 °C.

^{1}H NMR (600 MHz, CD_2Cl_2, 295 K), δ_{1H} [ppm] = **7.96** (d, $^3J_{HH}$ = 5.2 Hz, 1H, *H2*), **7.85-7.82** (m, 4H, *PPh_4^+*), **7.69-7.65** (m, 8H, *PPh_4^+*), **7.58-7.54** (m, 8H, *PPh_4^+*), **6.66** (d, $^3J_{HH}$ = 5.2 Hz, 1H, *H3*), **5.91-5.86** (m, 2H, *β–H*), **5.85** (d, $^3J_{HH}$ = 3.1 Hz, 1H, *β–H*), **5.74** (d, $^3J_{HH}$ = 3.0 Hz, 1H, *β–H*), **5.72** (d, $^3J_{HH}$ = 3.0 Hz, 1H, *β–H*), **5.69** (d, $^3J_{HH}$ = 3.1 Hz, 1H, *β–H*), **2.26-2.14** (m, 2H, *α-methylene*), **2.13-1.80** (m, 10H, *α-methylene*), **1.64-1.43** (m, 4H, *α-methylene*), **1.08** (t, $^3J_{HH}$ = 7.3, 3H), **0.97-0.84** (m, 9H, *α-Me*), **0.64-0.57** (m, 6H, *α-Me*), **0.49-0.42** (m, 6H, *α-Me*).

^{13}C{^{1}H} NMR (151 MHz, CD_2Cl_2, 295 K), δ_{13C} [ppm]: **191.1** (C_q, *C4*, was identified by ^{1}H,^{13}C HMBC NMR spectroscopy), **156.1** (CH, *C2*), **149.6** (C_q, *C_q-pyrrole*), **144.0** (C_q, *C_q-pyrrole*, was identified by ^{1}H,^{13}C HMBC NMR spectroscopy) **142.9** (C_q, *C_q-pyrrole*), **142.7** (C_q, *C_q-pyrrole*, was identified by ^{1}H,^{13}C HMBC NMR spectroscopy), **139.8** (C_q, *C_q-pyrrole*), **136.1** (d, $^4J_{CP}$ = 3.1 Hz, CH, *PPh_4^+*), **135.3** (C_q, *C_q-pyrrole*), **134.7** (d, $^2J_{CP}$ = 10.3 Hz, CH, *PPh_4^+*), **130.9** (d, $^3J_{CP}$ = 12.9 Hz, CH, *PPh_4^+*), **127.6** (CH, *C3*), **117.7** (d, $^1J_{CP}$ = 89.5 Hz, C_q, *PPh_4^+*), **107.0** (CH, *β-C*), **106.1** (CH, *β-C*), **104.7** (CH, *β-C*), **104.0** (C_q, *C1*), **103.5** (CH, *β-C*), **103.3** (CH, *β-C*), **101.9** (CH, *β-C*), **50.1** (C_q, *α-C*), **47.4** (C_q, *α-C*), **46.2** (CH_2, *α-methylene*), **45.5** (C_q, *α-C*), **44.6** (C_q, *α-C*, was identified by ^{1}H,^{13}C HMBC NMR spectroscopy), **36.5** (CH_2, *α-methylene*, was identified by ^{1}H,^{13}C HMBC NMR spectroscopy), **35.4** (CH_2, *α-methylene*, was identified by ^{1}H,^{13}C HMBC and ^{13}C{^{1}H}-DEPT-135 NMR spectroscopy), **32.8** (CH_2, *α-methylene*), **30.9** (CH_2, *α-methylene*), **30.0** (CH_2, *α-methylene*), **28.0** (CH_2, *α-methylene*), **23.6** (CH_2, *α-methylene*, was identified by ^{1}H,^{13}C HMBC and ^{13}C

DEPT-135 NMR spectroscopy), **10.7** (CH_3, *α-Me*), **10.6** (CH_3, *α-Me*), **10.2** (CH_3, *α-Me*), **10.1** (CH_3, *α-Me*), **9.9** (CH_3, *α-Me*), **9.8** (CH_3, *α-Me*), **9.5** (CH_3, *α-Me*), **8.2** (CH_3, *α-Me*).

<u>**^{31}P{^{1}H} NMR** (243 MHz, CD_2Cl_2, 295 K),</u> δ_{31P} [ppm]: 23.15.

<u>**HR-MS** (ESI, negative ion mode):</u> *m/z* calculated for $[M\text{-}NO]^{\bullet -}$: **593.3679**, found: **593.3804**.

<u>**UV-Vis** (CH_2Cl_2, room temperature),</u> λ_{max} [nm] = **445**, **342**.

<u>**FT-ATR-IR** (solid-state, room temperature),</u> $\tilde{\nu}_{max}$ [cm^{-1}] = **3084** (w), **3054** (w), **2960** (s), **2922** (s), **2866** (s), **1617** (m), **1586** (m), **1436** (s), **1365** (s), **1317** (m), **1107** (s), **1076** (s), **996** (m), **884** (m), **860** (s), **750** (s), **720** (s), **688** (s).

2.6 [PPh_4][^{Me}**2***-PhNO]

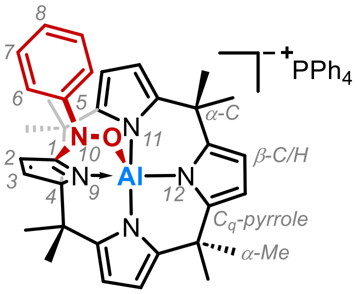

Procedure. In a nitrogen-filled glovebox, [PPh_4][^{Me}**1**] (100 mg, 119 µmol, 1 eq.) was dissolved in dichloromethane (3 mL). Nitrosobenzene (12.84 mg, 119.90 µmol, 1.00 eq.) was added in one portion. The reaction mixture was stirred for 1 h at room temperature. Afterwards the solvent was removed under reduced pressure. The product was obtained as a golden brown solid (64.2 mg, 114.91 µmol, 91 %).

Single crystals suitable for X-ray diffraction were grown by vapor diffusion of *n*-pentane into a DCM/ toluene solution.

<u>**^{1}H NMR** (600 MHz, CD_2Cl_2, 295 K),</u> δ_{1H} [ppm] = **7.83-7.80** (m, 4H, *PPh_4^+*), **7.66-7.63** (m, 8H, *PPh_4^+*), 7.58 (d, $^3J_{HH}$ = 5.2 Hz, 1H, *H3*), **7.54-7.49** (m, 8H, *PPh_4^+*), **7.38-7.36** (m, 2H, *H6*), **7.24-7.21** (m, 2H, *H7*), **6.93-6.90** (m, 1H, *H8*), **6.62** (d, $^3J_{HH}$ = 5.2 Hz, 1H, *H2*), **5.60** (s, 2H, *β-H*), **5.57** (d, $^3J_{HH}$ = 2.8 Hz, 2H, *β–H)*, **5.56** (d, $^3J_{HH}$ = 2.8 Hz, 2H, *β-H)*, **1.70** (s, 6H, *α-Me*), **1.57** (s, 6H, *α-Me*), **1.42** (s, 6H, *α-Me*), **1.21** (s, 6H, *α-Me*).

<u>**^{13}C{^{1}H} NMR** (151 MHz, CD_2Cl_2, 295 K),</u> δ_{13C} [ppm] = **161.2** (CH, *C3*), **157.9** (C_q, *C1*), **149.0** (C_q, *C_q-pyrrole*), **148.3** (C_q, *C_q-pyrrole*), **147.4** (C_q, *C_q-pyrrole*), **136.1** (d, $^4J_{CP}$ = 3.0 Hz, CH, *PPh_4^+*), **134.7** (d, $^2J_{CP}$ = 10.6 Hz, CH, *PPh_4^+*), **131.0** (d, $^3J_{CP}$ = 13.6 Hz, CH, *PPh_4^+*), **129.0** (CH, *C7*), **122.3** (CH, *C8*), **120.8** (CH, *C2*), **117.8** (d,

$^1J_{CP}$ = 90.6 Hz, C_q, *PPh$_4^+$*), **116.6** (CH, *C6*), **102.0** (CH, *β-C*), **100.0** (CH, *β-C*), **99.2** (CH, *β-C*), **81.3** (C_q, *C4*), **46.2** (C_q, *α-C*), **37.3** (C_q, *α-C*), **37.0** (CH_3, *α-Me*), **32.2** (CH_3, *α-Me*), **30.0** (CH_3, *α-Me*), **27.0** (CH_3, *α-Me*).

^{1}H,^{15}N HMBC NMR (600 MHz, 61 MHz, CD_2Cl_2, 295 K), δ_{15N} [ppm] (with respect to nitromethane reference) = **-169.2** (N-9), **-184.25** (N-11), **-191.79** (N-12), **-210.65** (N-10).

HR-MS (ESI, negative ion mode): *m/z* calculated for $C_{34}H_{37}AlN_5O$ $[M]^-$ **558.2813**, found: **558.2817**.

UV-Vis (CH_2Cl_2, room temperature), λ_{max} [nm] = **359**, **275**, **268**, **262**.

FT-ATR-IR (solid-state, room temperature), $\tilde{\nu}_{max}$ [cm^{-1}] = **3079** (w), **3069** (s), **2960** (m), **2858** (w), **1616** (m), **1590** (s), **1497** (s), **1435** (s), **1372** (m), **1159** (m), **1107** (s), **1168** (s), **995** (m), **754** (s), **720** (s), **687** (s).

Elemental analysis: calc. C 77.57, H 6.40, N 7.80, found: C 76.73, H 6.65, N 7.25.

2.7 [PPh$_4$][^{Me}**1***-PhNO]

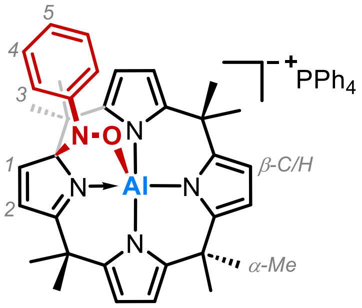

Procedure. In a J. Young NMR tube [PPh$_4$][^{Me}**1**] (15 mg, 17.98 µmol, 1.00 eq.) was dissolved in dichloromethane-*d$_2$* (0.25 mL). In a GC-vial, nitrosobenzene (1.93 mg, 17.98 µmol, 1.00 eq.) was dissolved in dichloromethane-*d$_2$* (0.15 mL). The latter solution was added to the former at -78 °C. The color of the reaction mixture turned within seconds to orange. Immediately after the addition, the sample was brought to the NMR spectrometer while cooling at -78 °C and analyzed by at least ^{1}H NMR spectroscopy.

^{1}H NMR (600 MHz, CD_2Cl_2, 225 K), δ_{1H} [ppm] = **7.86-7.81** (m, 4H, *PPh$_4^+$*), **7.67-7.63** (m, 8H, *PPh$_4^+$*), **7.53-7.48** (m, 8H), **7.06** (d, $^3J_{HH}$ = 5.3 Hz, 1H, *H1*), **6.86-6.81** (m, 2H, *H4*), **6.74-6.66** (m, 3H, *H3 & H5*), **6.17** (d, $^3J_{HH}$ = 5.3 Hz, 1H, *H2,* identified by ^{1}H,^{1}H COSY NMR spectroscopy), **5.93** (d, $^3J_{HH}$ = 2.7 Hz, 1H, *β-H*), **5.84** (d, $^3J_{HH}$ = 2.6 Hz, 1H, *β-H*), **5.77** (m, 2H, 2x*β-H*), **5.70** (d, $^3J_{HH}$ = 2.8 Hz, 1H, *β-H*), **5.67** (d, $^3J_{HH}$ = 2.7 Hz, 1H, *β-H*), **1.86** (s, 3H, *α-Me*), **1.56** (m, 15H, *α-Me*), **1.31** (s, 3H, *α-Me*), **0.92** (s, 3H, *α-Me*).

^{1}H,^{13}C HSQC NMR (600 MHz, 151 MHz, CD_2Cl_2, 240 K), δ_{13C} [ppm] = **157.5** (CH, *C1*), **135.4** (CH, *PPh$_4^+$*), **134.0** (CH, *PPh$_4^+$*), **130.2** (CH, *PPh$_4^+$*), **127.3** (CH, *C4*), **124.4** (CH, *C2*), **121.5** (CH, *C3/C5*), **102.4** (CH, *β-C*),

101.7 (CH, *β-C*), **100.7** (CH, *β-C*), **100.3** (CH, *β-C*), **98.3** (CH, *β-C*), **40.7** (CH_3, *α-Me*), **35.2** (CH_3, *α-Me*), **35.1** (CH_3, *α-Me*), **28.8** (CH_3, *α-Me*), **27.1** (CH_3, *α-Me*), **26.7** (CH_3, *α-Me*), **25.8** (CH_3, *α-Me*), **22.8** (CH_3, *α-Me*).

3 Reactivity tests with phosphanes

[PPh_4][^{Et}**1**] (1.0 eq.) was dissolved in dichloromethane-*d_2* and 2.75 bar of NO were added. Afterwards, the solution was degassed by four freeze-pump-thaw cycles. Equimolar amounts of P(*para*-F-C_6H_4)$_3$ (5 mg) were added. The reaction was monitored by ^{31}P{^{1}H} NMR spectroscopy (Figure S2). Furthermore, the oxidation of P(*para*-F-C_6H_4)$_3$ by NO was studied upon addition of 10 mol% of [PPh_4][^{Et}**1**]. Therefore, P(*para*-F-C_6H_4)$_3$ (5 mg) and [PPh_4][^{Et}**1**] (10 mol%) were dissolved in dichloromethane-*d_2*. Afterwards, NO (2.75 bar) was added. The reaction was also followed by ^{31}P{^{1}H} NMR spectroscopy (Figure S3). For direct comparison, the oxidation of P(*para*-F-C_6H_4)$_3$ (5 mg) by NO under the same reaction conditions without [PPh_4][^{Et}**1**] or preliminary formed [PPh_4][^{Et}**1***-ONNO] was studied at room temperature (Figure S1).

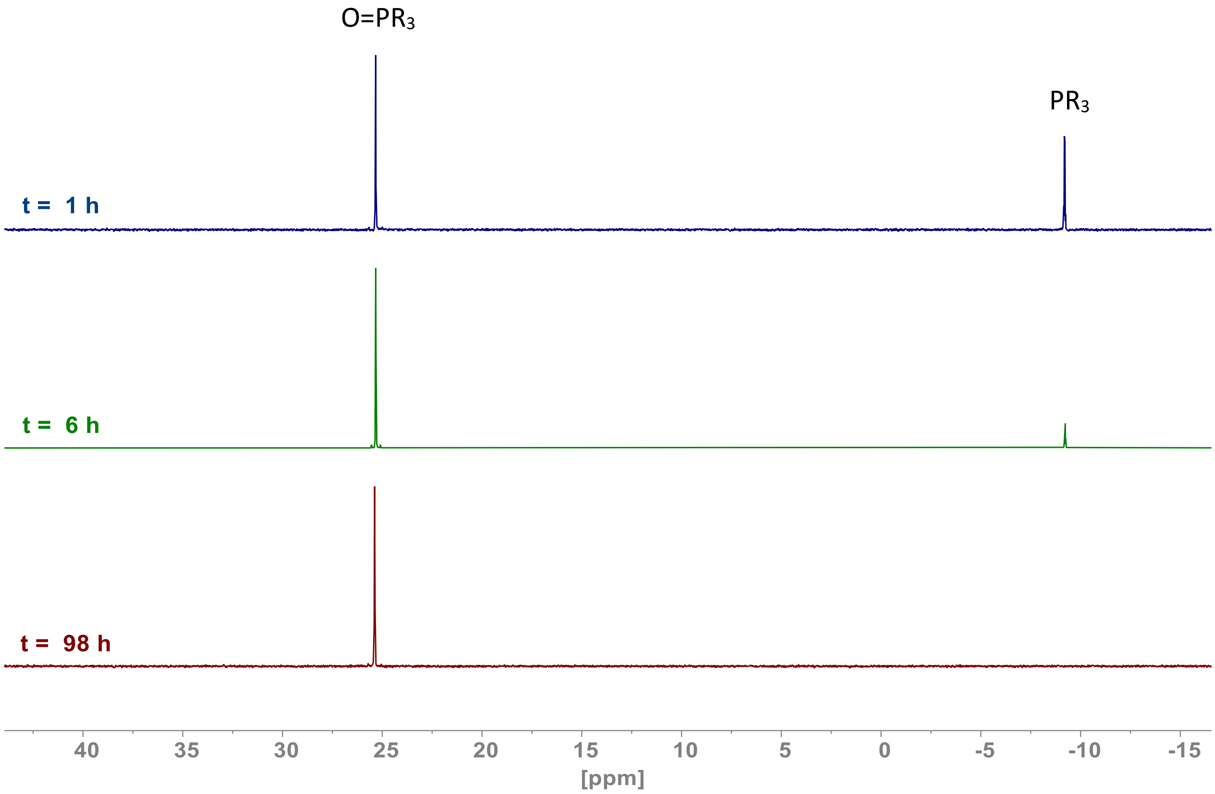

Figure S1. ^{31}P{^{1}H} NMR spectra (162 MHz, CD_2Cl_2, 295 K) of the of the studied oxidation reaction of P(*para*-F-C_6H_4)$_3$ with NO (R = *para*-F-C_6H_4).

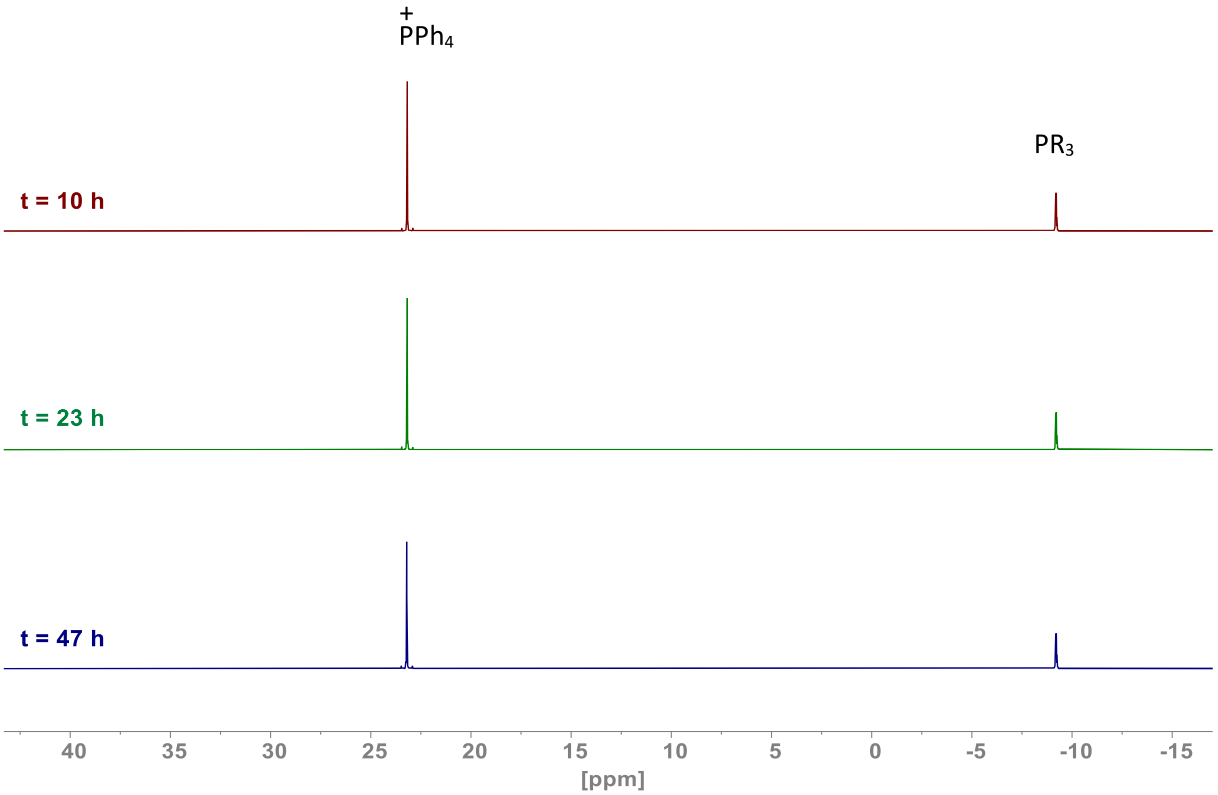

Figure S2. $^{31}P\{^{1}H\}$ NMR spectra (162 MHz, CD_2Cl_2, 295 K) of the studied oxidation reaction of P(*para*-F-C_6H_4)$_3$ in the presence of 1 eq. [PPh_4][^{Et}**1***-ONNO] (R = *para*-F-C_6H_4).

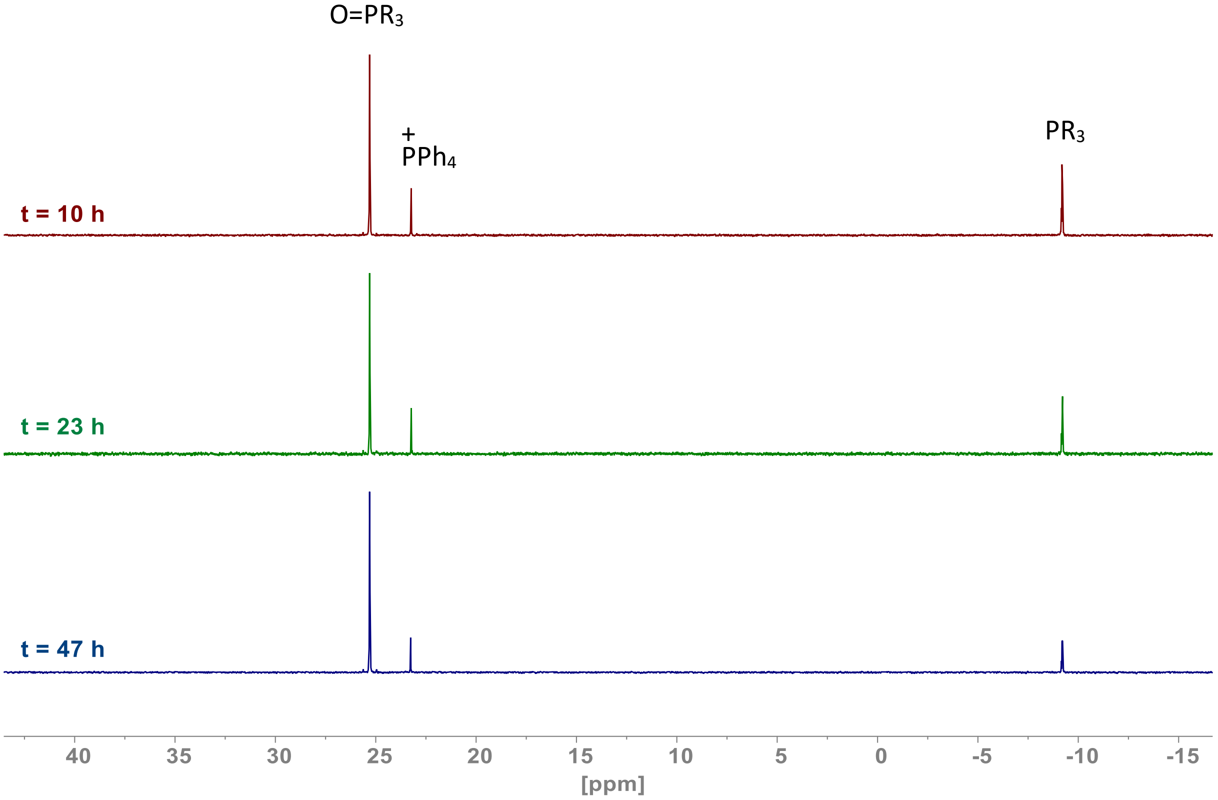

Figure S3. $^{31}P\{^{1}H\}$ NMR spectra (162 MHz, CD_2Cl_2, 295 K) of the studied oxidation reaction of P(*para*-F-C_6H_4)$_3$ with NO in the presence of 10 mol% of [PPh_4][^{Et}**1**] (R = *para*-F-C_6H_4).

4 Decomposition of [PPh_4][^{Et}1*-ONNO]

When dissolved in DCM, [PPh_4][^{Et}**1***-ONNO] decomposes over time or if heated at 60 °C for over 2 h. During this decomposition a new species is formed, which itself decomposes over time at room temperature or if heated at 60 °C. Interestingly, the ratio of the new species and [PPh_4][^{Et}**1***-ONNO] never exceeds 1:1 in the conducted experiments. We were not able to isolate the new species.

5 Determination of the Rearrangement Barrier

For the determination of the rearrangement barrier, [PPh_4][^{Me}**1**] (15 mg, 18.96 µmol,1.0 eq.) was dissolved in CD_2Cl_2 (0.25 µL) in a J. Young NMR tube. In a vial, nitrosobenzene (2.03 mg, 18.96 µmol, 1.0 eq.) was dissolved in CD_2Cl_2 (0.15 µL). The two solutions were combined at -78 °C. While cooling at -78 °C the J. Young NMR tube was brought to the NMR spectrometer, which was already at 260 K. ^{1}H NMR experiments were conducted. Assuming that the substrate was fully consumed and converted to [PPh_4][^{Me}**1***-PhNO] and that [PPh_4][^{Me}**1***-PhNO] is selectively converted to [PPh_4][^{Me}**2***-PhNO], a reaction barrier can be calculated from this data.

Calculation of the Rearrangement Barrier

The concentration of [PPh_4][^{Me}**1***] was determined using NMR integrals. Uncertainty was calculated assuming an integral error of 0.05 with *Gaussian* error propagation. By calibrating the spectra to the solvent signal, the signals at 6.2 ppm and 6.6 pm were used for the barrier determination. The resonance at 6.2 ppm represents one β-proton of the dearomatized pyrrole unit of [PPh_4][^{Me}**1***-PhNO]. The signal at 6.6 pm can be assigned to one β-proton of the dearomatized pyrrole moiety of [PPh_4][^{Me}**2***-PhNO]. The integral of the signal at 6.2 ppm referring to the 1,2-adduct [PPh_4][^{Me}**1***-PhNO] was set to 1. The respective integral values of the resonance at 6.3 ppm and the thereof derived concentrations of [PPh_4][^{Me}**2***-PhNO] are listed in Table S1 and the corresponding spectra are shown in Figure S4.

Table S1. Integral values used and the derived concentrations of [PPh_4][^{Me}**2***-PhNO] for the assessment of the rearrangement barrier. The signals were integrated employing an integration region file in order to integrate the same region each time (V_{tot} = 0.4 mL).

**t [s]**	960	1560	2160	3360	4560	5760	6960	8100	9360
**Integral value**	0.32	0.41	0.55	0.82	1.13	1.55	1.97	2.43	3.07
**c [mmol L^{-1}]**	11.49	13.78	16.82	21.36	25.15	28.81	31.44	33.58	35.75

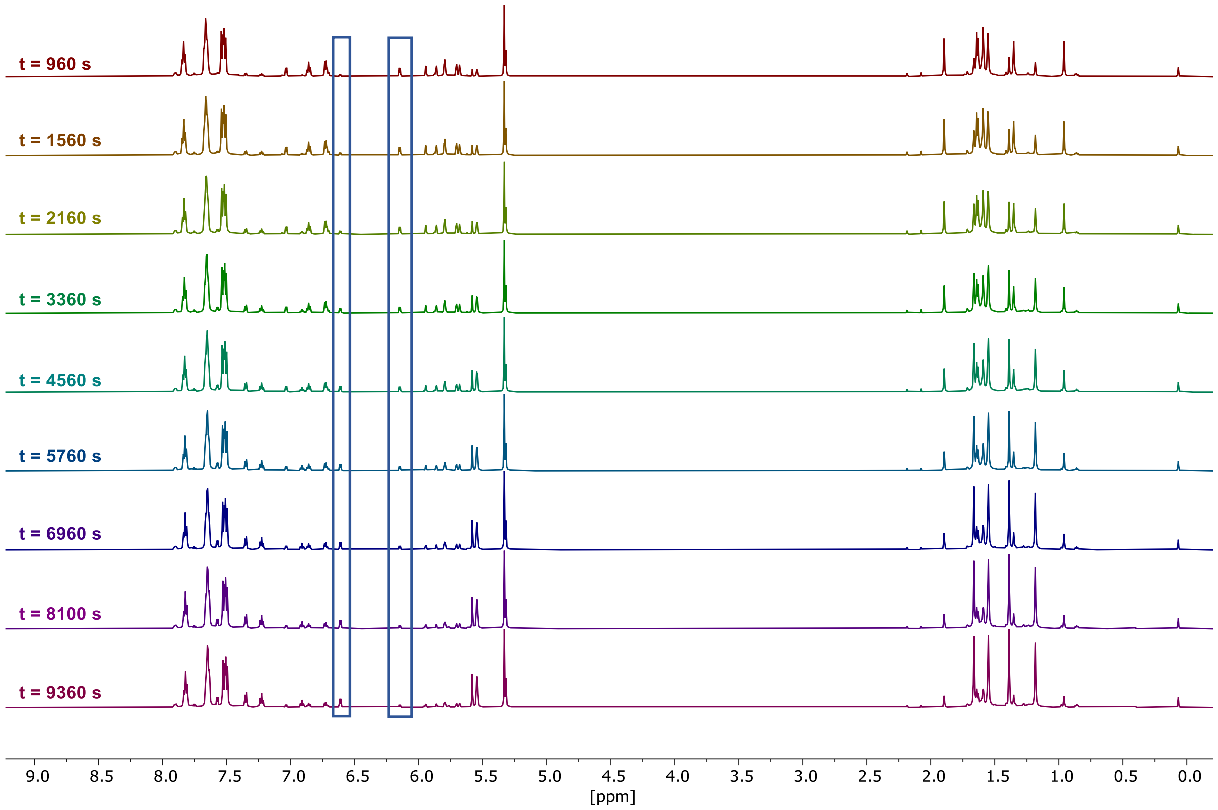

Figure S4. ^{1}H NMR (600 MHz, CD_2Cl_2, 260 K) spectra for the determination of the barrier of the rearrangement of $[PPh_4][^{Me}$**1***-PhNO] to $[PPh_4][^{Me}$**2***-PhNO]. The marked regions show the resonances used for the assessment of the rearrangement barrier.

Assuming that the rearrangement is a first order reaction, the chemical rate constant *k* can be derived by the graphical display of:

$$ln\left(\frac{[A]_0}{[A]_0 - x}\right) = kt. \qquad (1)$$

The resulting plot is shown in Figure S5.

The obtained rate constant *k* was used to estimate the Gibbs free activation energy at 260 K for the rearrangement barrier as followed:

$$\Delta_R G^{\ddagger} = -R \cdot T \cdot ln\left(\frac{k \cdot h}{\kappa \cdot k_B \cdot T}\right), \qquad \kappa = 1$$

$$\Delta\left(\Delta_R G^{\ddagger}\right) = \frac{RT}{k} \cdot \Delta k$$

$$k = (1.36 \pm 0.39) \cdot 10^{-4}\ s^{-1}$$

$$\Delta_R G^{\ddagger} = (82.6 \pm 0.6)\ kJ\ mol^{-1}$$

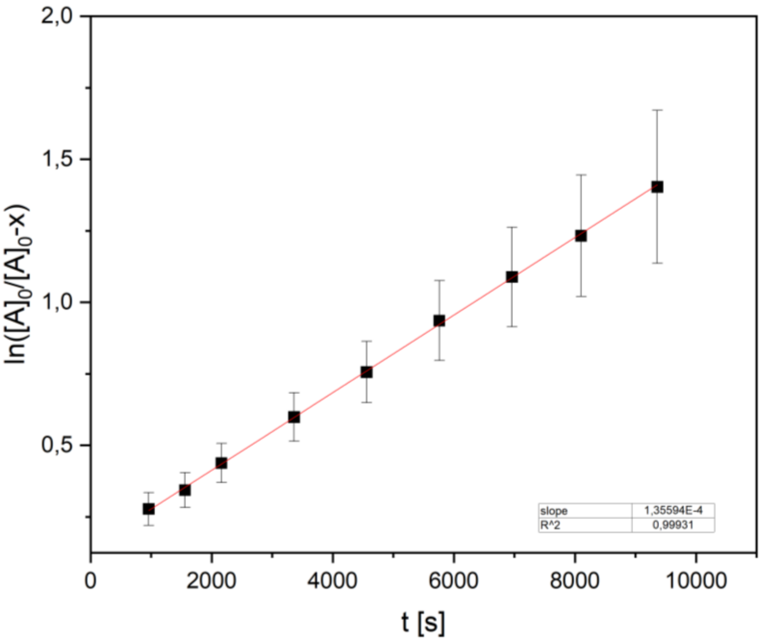

Figure S5. Graphical display of equation 1 used for the determination of the rate constant *k*.

Ultimately, a rearrangement barrier of 82.6 kJ mol^{-1} can be assessed. The assumed integral uncertainty of 0.05 propagates to an uncertainty of 0.6 kJ mol^{-1} of the final rearrangement barrier (not considering used approximations and possible systematic errors).

6 X-ray crystallography

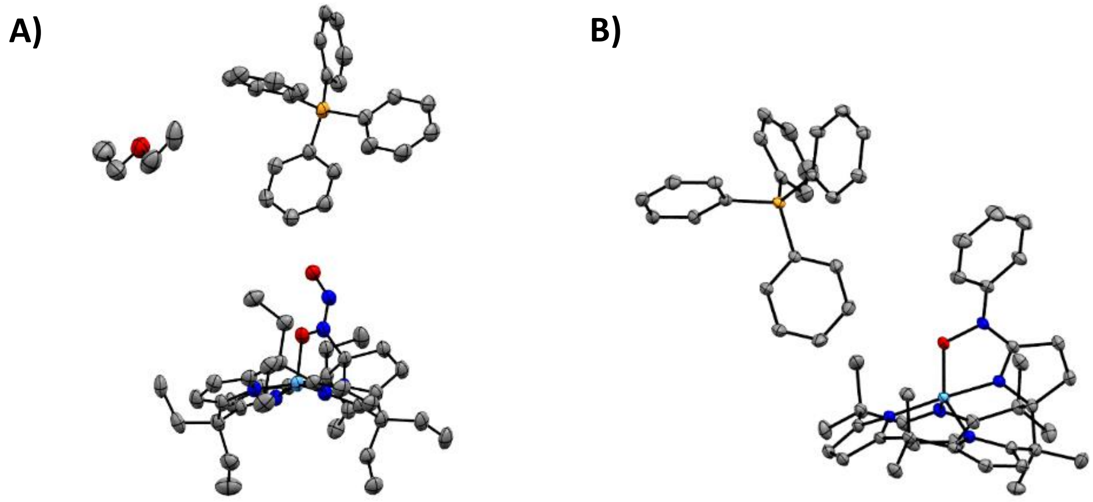

Figure S6. Asymmetric unit of A) containing [PPh_4][^{Et}**1***-ONNO] and one diethyl ether molecule and of B) containing [PPh_4][^{Et}**1***-ONNO]. Thermal ellipsoids are shown at the 50% probability level.

Each crystal was mounted on a MiTeGen micromount with perfluoroether oil. Data were collected from a shock-cooled single crystal at 100(2) K on a Bruker D8 VENTURE dual wavelength Mo/Cu four-circle diffractometer with a microfocus sealed X-ray tube using a mirror optics as monochromator and a Bruker PHOTON III detector. The diffractometer was equipped with an Oxford Cryostream 700 low

temperature device and used MoK_α radiation (λ = 0.71073 Å). All data were integrated with SAINT and a multi-scan absorption correction using SADABS was applied.[6, 7] The structure was solved by direct methods using SHELXT and refined by full-matrix least-squares methods against F^2 by SHELXL-2019/2.[8, 9] All non-hydrogen atoms were refined with anisotropic displacement parameters. All hydrogen atoms were refined isotropic on calculated positions using a riding model with their U_{iso} values constrained to 1.5 times the U_{eq} of their pivot atoms for terminal sp^3 carbon atoms and 1.2 times for all other carbon atoms. Crystallographic data for the structures reported here have been deposited with the Cambridge Crystallographic Data Centre.[10] CCDC 2342027 and 2342028 contain the supplementary crystallographic data for this paper. These data can be obtained free of charge from The Cambridge Crystallographic Data Centre via www.ccdc.cam.ac.uk/structures. This report and the CIF file were generated using FinalCif.[11]

Table S2. Crystal data and structure refinement for [PPh$_4$][^{Et}**1***-ONNO] and [PPh$_4$][^{Me}**2***-PhNO]

	[PPh$_4$][^{Et}**1***-ONNO]	[PPh$_4$][^{Me}**2***-PhNO]
CCDC number	2342027	2342028
Empirical formula	$C_{64}H_{78}AlN_6O_3P$	$C_{58}H_{57}AlN_5OP$
Formula weight	1037.27	898.03
Temperature [K]	100(2)	100(2)
Crystal system	monoclinic	triclinic
Space group (number)	$P2_1/n$ (14)	$P\overline{1}$ (2)
a [Å]	11.8687(17)	12.7974(8)
b [Å]	27.971(4)	13.9223(8)
c [Å]	21.374(3)	17.5632(10)
α [°]	90	72.594(2)
β [°]	95.254(5)	80.317(2)
γ [°]	90	63.381(2)
Volume [Å^3]	7066.1(18)	2667.3(3)
Z	4	2
ρ_{calc} [g cm^{-3}]	0.975	1.118
μ [mm^{-1}]	0.093	0.110
F (000)	2224	952
Crystal size [mm^3]	0.35×0.32×0.22	0.45×0.30×0.11
Crystal colour	red	yellow
Crystal shape	palte	block
Radiation	MoK_α (λ = 0.71073 Å)	MoK_α (λ = 0.71073 Å)

2θ range [°]	3.74 to 52.97 (0.80 Å)	4.23 to 67.62 (0.64 Å)
Index ranges	−14 ≤ h ≤ 14<br>−34 ≤ k ≤ 35<br>−26 ≤ l ≤ 26	−19 ≤ h ≤ 20<br>−21 ≤ k ≤ 20<br>−26 ≤ l ≤ 27
Reflections collected	198888	180426
Independent reflections	14534<br>R_{int} = 0.0923<br>R_{sigma} = 0.0421	20863<br>R_{int} = 0.0562<br>R_{sigma} = 0.0364
Completeness to θ = 25.242°	99.9 %	99.9 %
Data / Restraints / Parameters	14534/900/688	20863/1245/603
Absorption correction T_{min}/T_{max} (method)	0.6511/0.7454<br>(multi-scan)	0.711/0.7467<br>(multi-scan)
Goodness-of-fit on F^2	1.040	1.029
Final *R* indexes [$I \geq 2\sigma(I)$]	R_1 = 0.0699<br>wR_2 = 0.1782	R_1 = 0.0424<br>wR_2 = 0.1122
Final *R* indexes [all data]	R_1 = 0.0953<br>wR_2 = 0.1949	R_1 = 0.0560<br>wR_2 = 0.1225
Largest peak/hole [e Å^{-3}]	0.40/−0.34	0.47/−0.41

7 NMR spectra

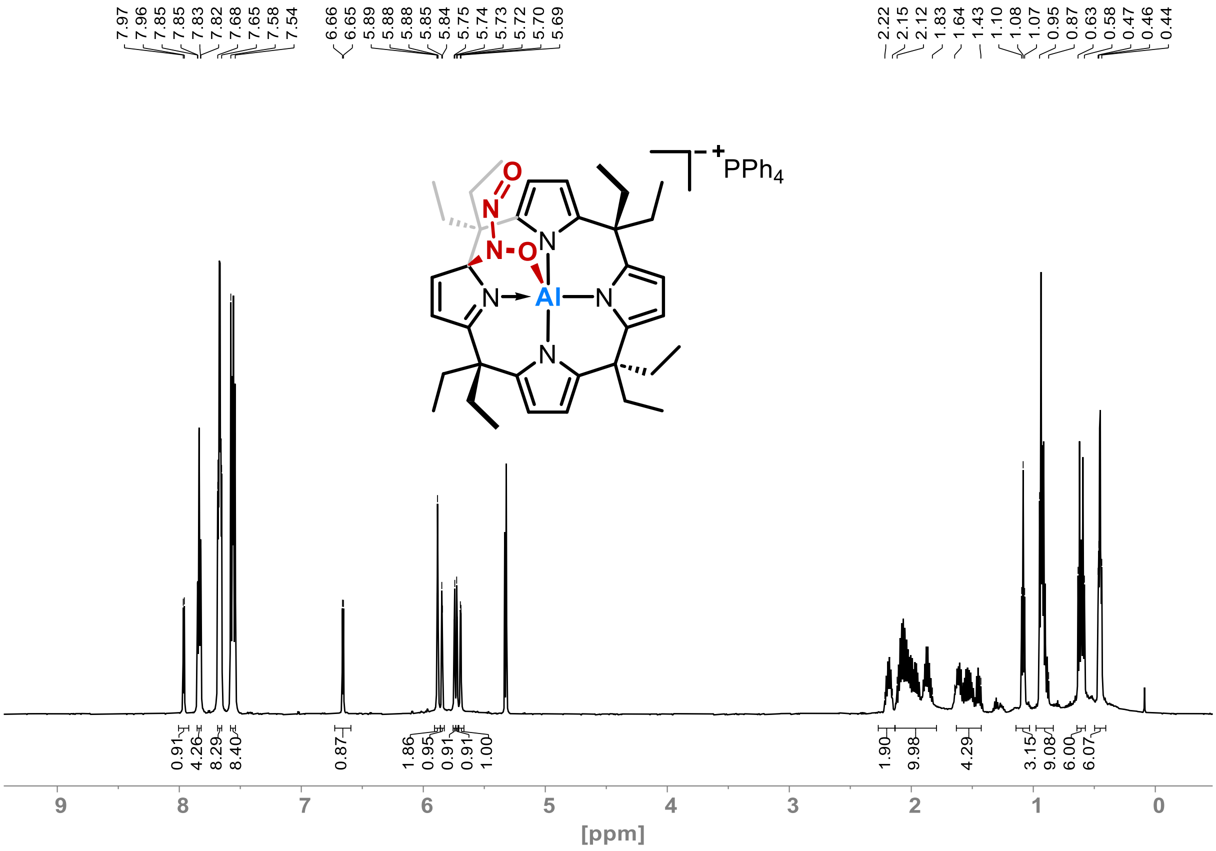

Figure S7. ^{1}H NMR spectrum (600 MHz, CD_2Cl_2, 295 K) of [PPh_4][^{Et}**1***-ONNO].

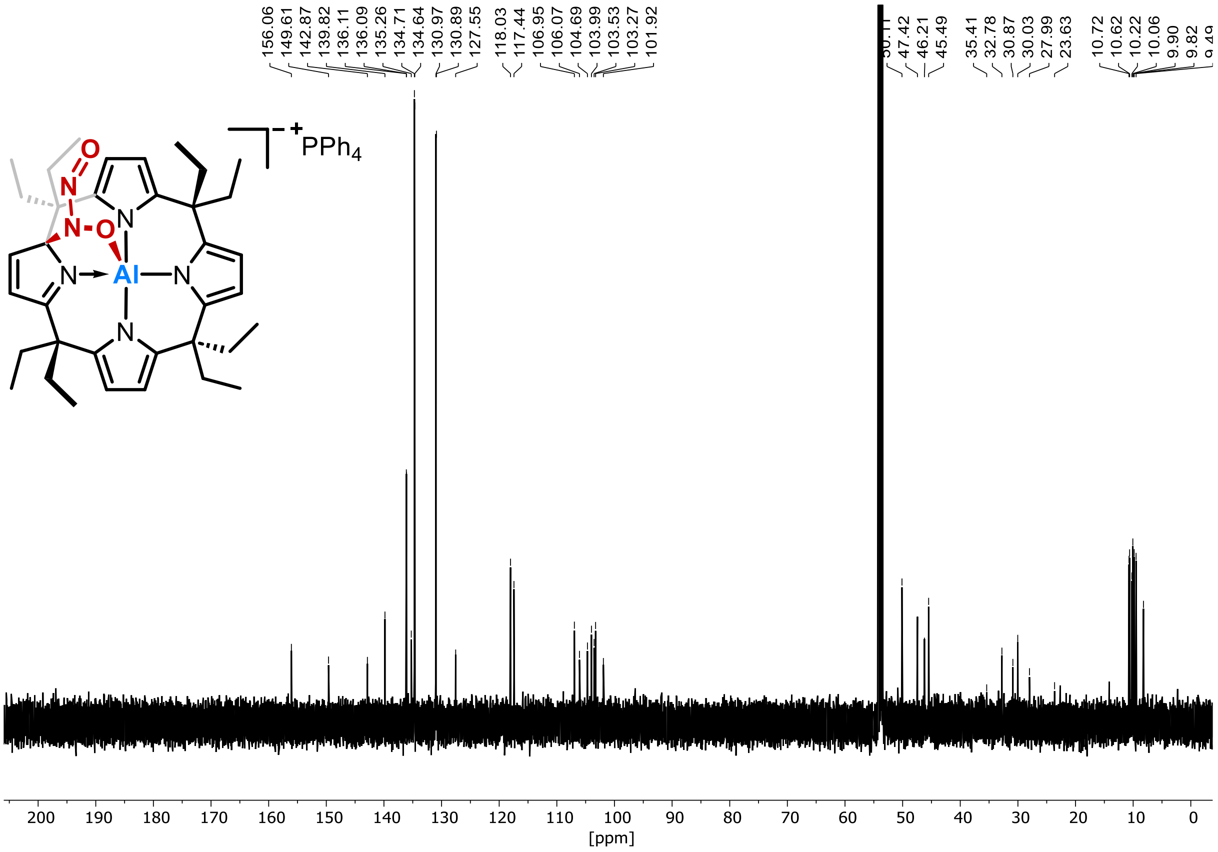

Figure S8. $^{13}C\{^{1}H\}$ NMR spectrum (151 MHz, CD_2Cl_2, 295 K) of [PPh_4][^{Et}**1***-ONNO].

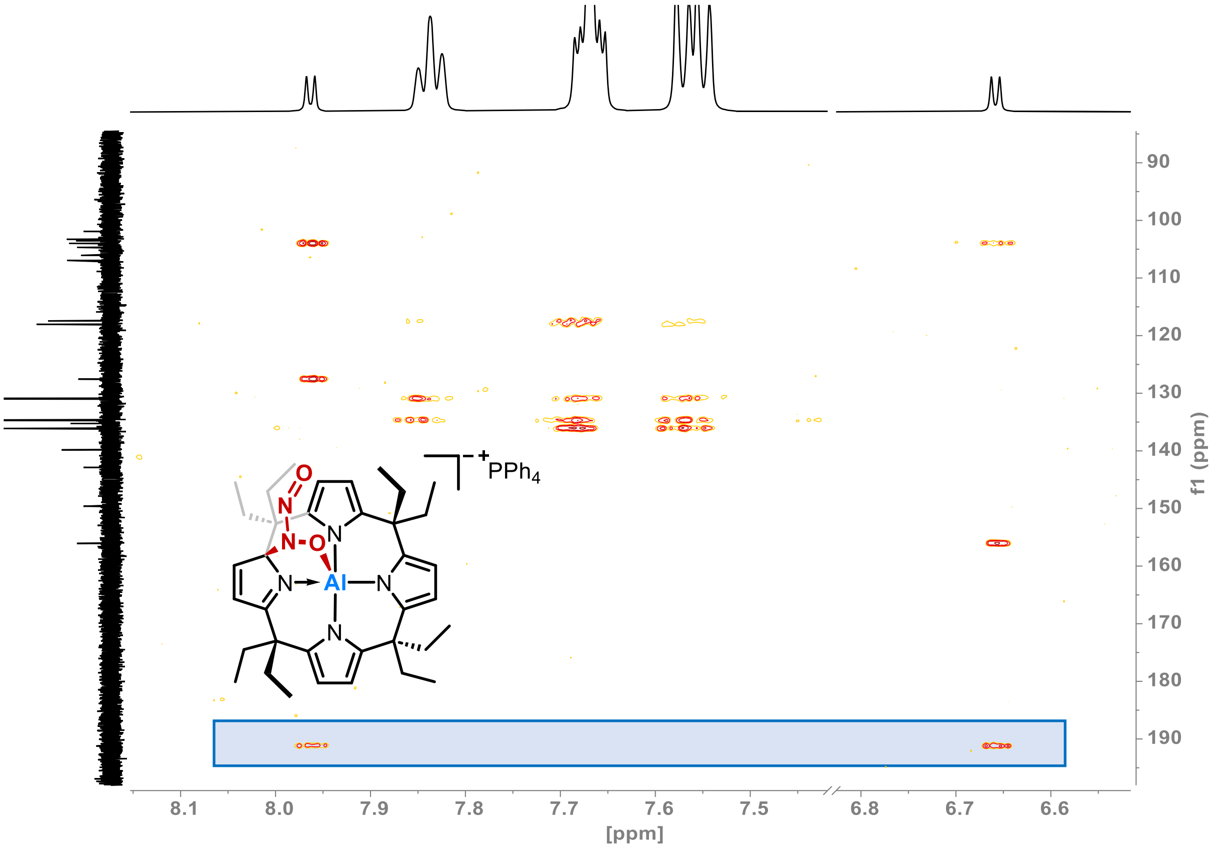

Figure S 9. Excerpt of the ^{1}H,^{13}C HMBC NMR spectrum (600 MHz, 151 MHz, CD_2Cl_2, 295 K) of [PPh_4][^{Et}**1***-ONNO]. The marked region shows a signal not found in the $^{13}C\{^{1}H\}$ NMR experiment.

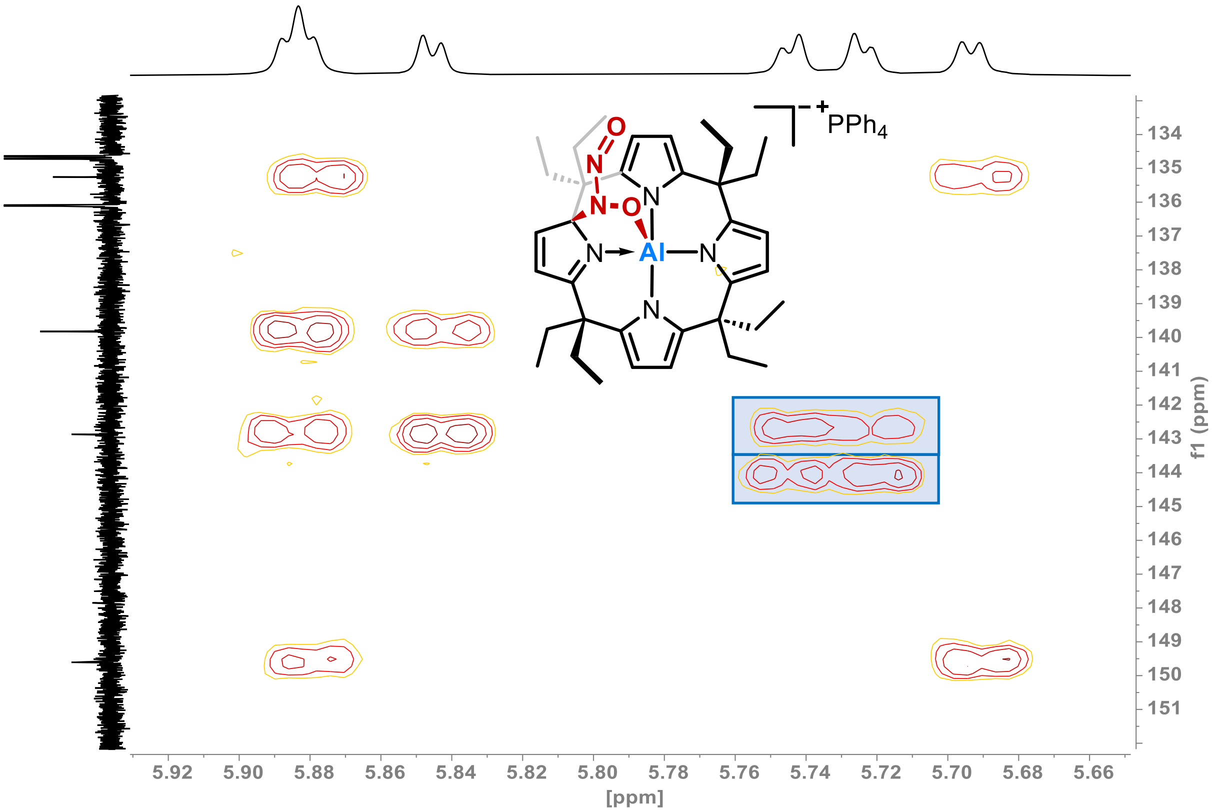

Figure S10. Excerpt of the $^{1}H,^{13}C$ HMBC NMR spectrum (600 MHz, 151 MHz, CD_2Cl_2, 295 K) of $[PPh_4][^{Et}$**1***-ONNO]. The marked regions show signals not found in the $^{13}C\{^{1}H\}$ NMR experiment.

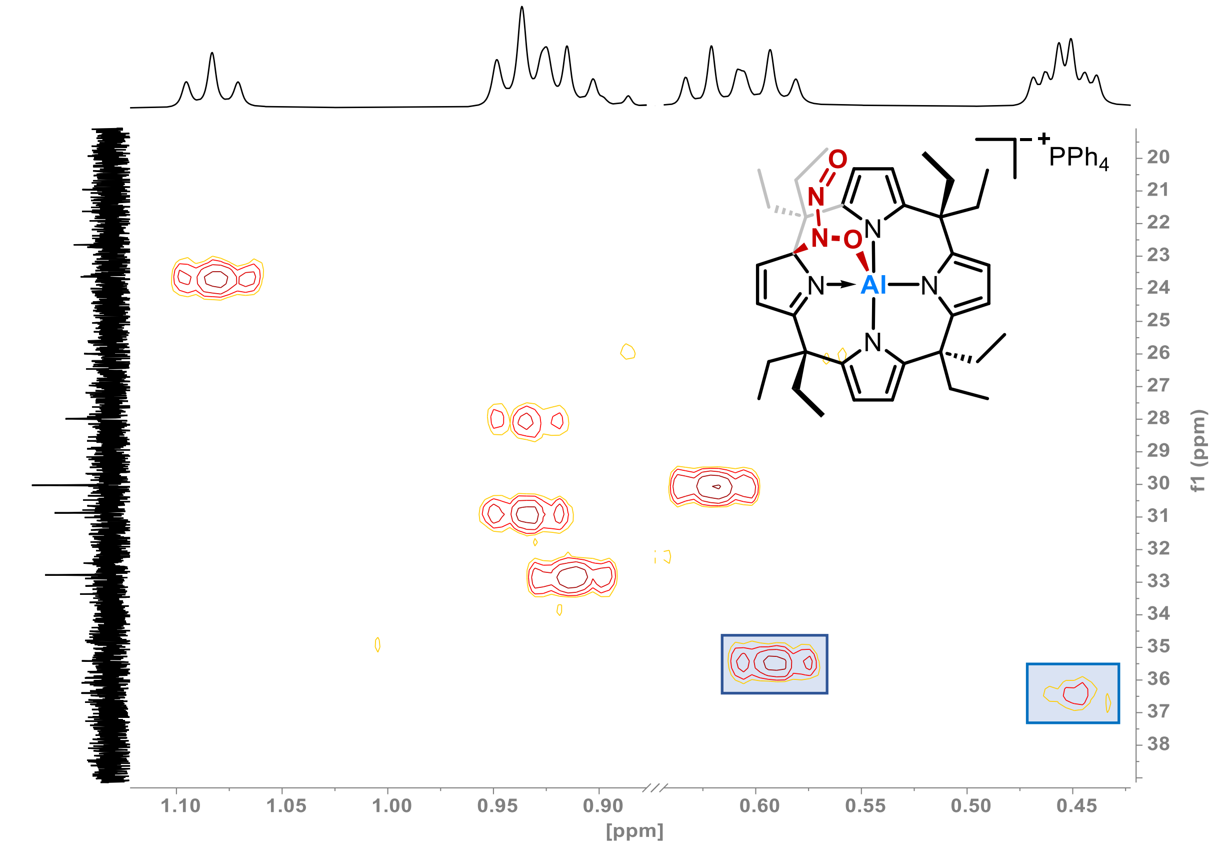

Figure S11. Excerpt of the $^{1}H,^{13}C$ HMBC NMR spectrum (600 MHz, 151 MHz, CD_2Cl_2, 295 K) of $[PPh_4][^{Et}$**1***-ONNO]. The marked regions show signals of methylene groups not found in the $^{13}C\{^{1}H\}$ NMR experiment.

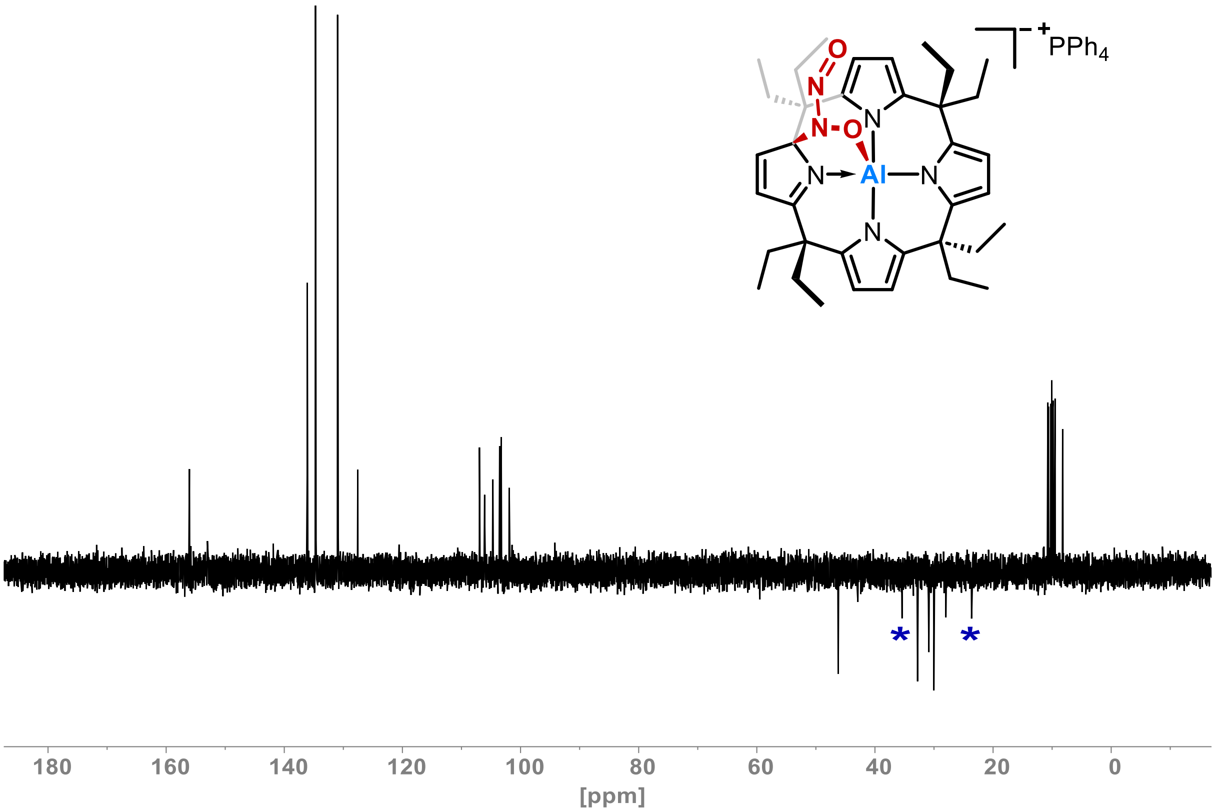

Figure S12. $^{13}C\{^1H\}$-DEPT135 NMR spectrum (151 MHz, CD_2Cl_2, 295 K) of $[PPh_4][^{Et}\mathbf{1}^*\text{-ONNO}]$. The marked signals show signals of methylene groups not found in the $^{13}C\{^1H\}$ NMR experiment.

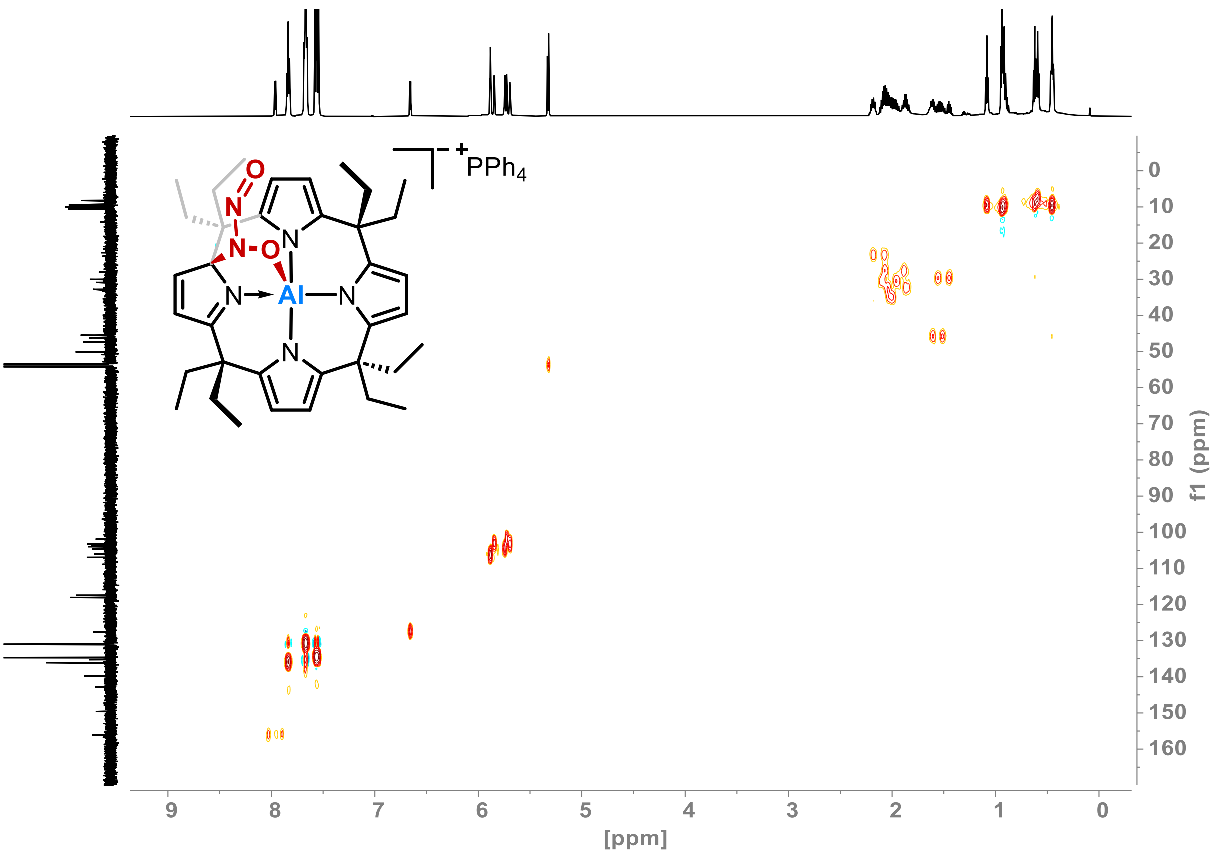

Figure S13. $^1H,^{13}C$ HSQC NMR spectrum (600 MHz, 151 MHz, CD_2Cl_2, 295 K) of $[PPh_4][^{Et}\mathbf{1}^*\text{-ONNO}]$.

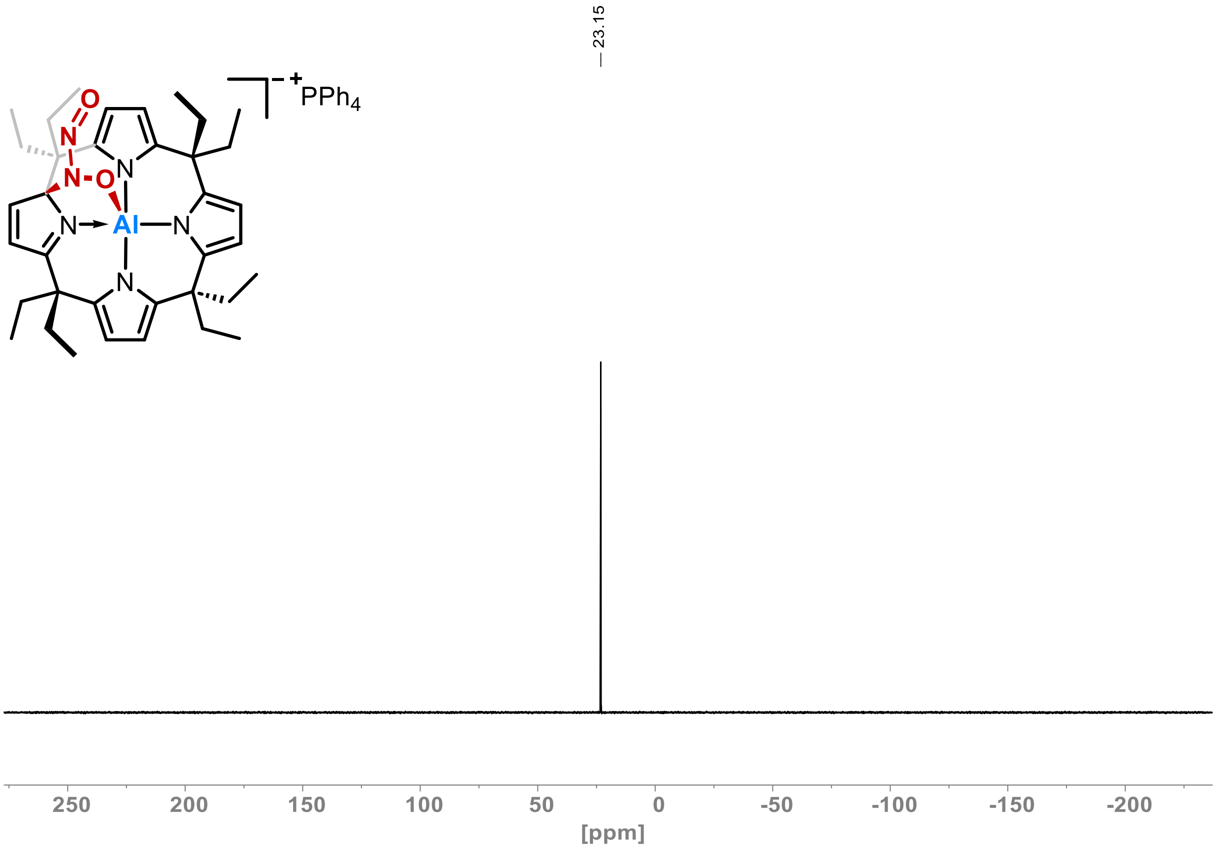

Figure S14. $^{31}P\{^{1}H\}$ NMR spectrum (243 MHz, CD_2Cl_2, 295 K) of $[PPh_4][^{Et}$**1***-ONNO].

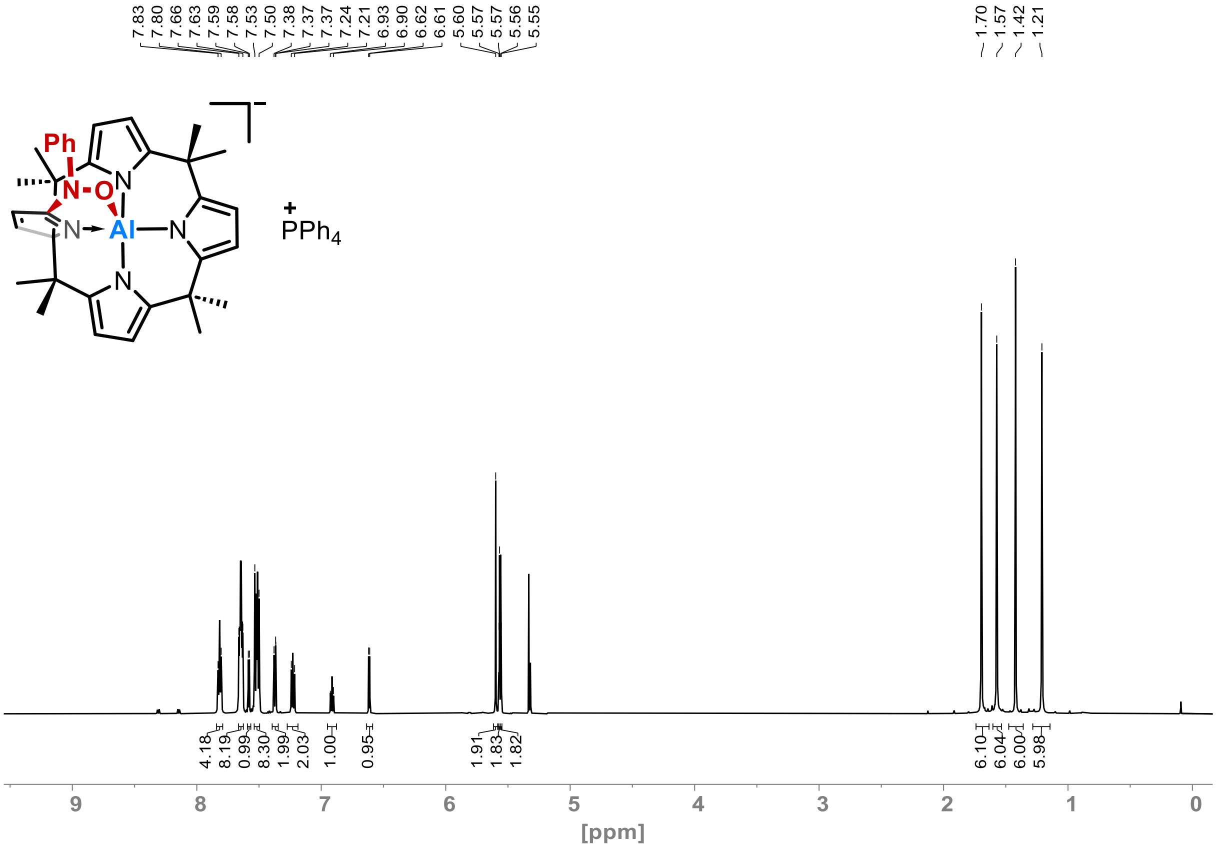

Figure S15. ^{1}H NMR spectrum (600 MHz, CD_2Cl_2, 295 K) of $[PPh_4][^{Me}$**2***-PhNO].

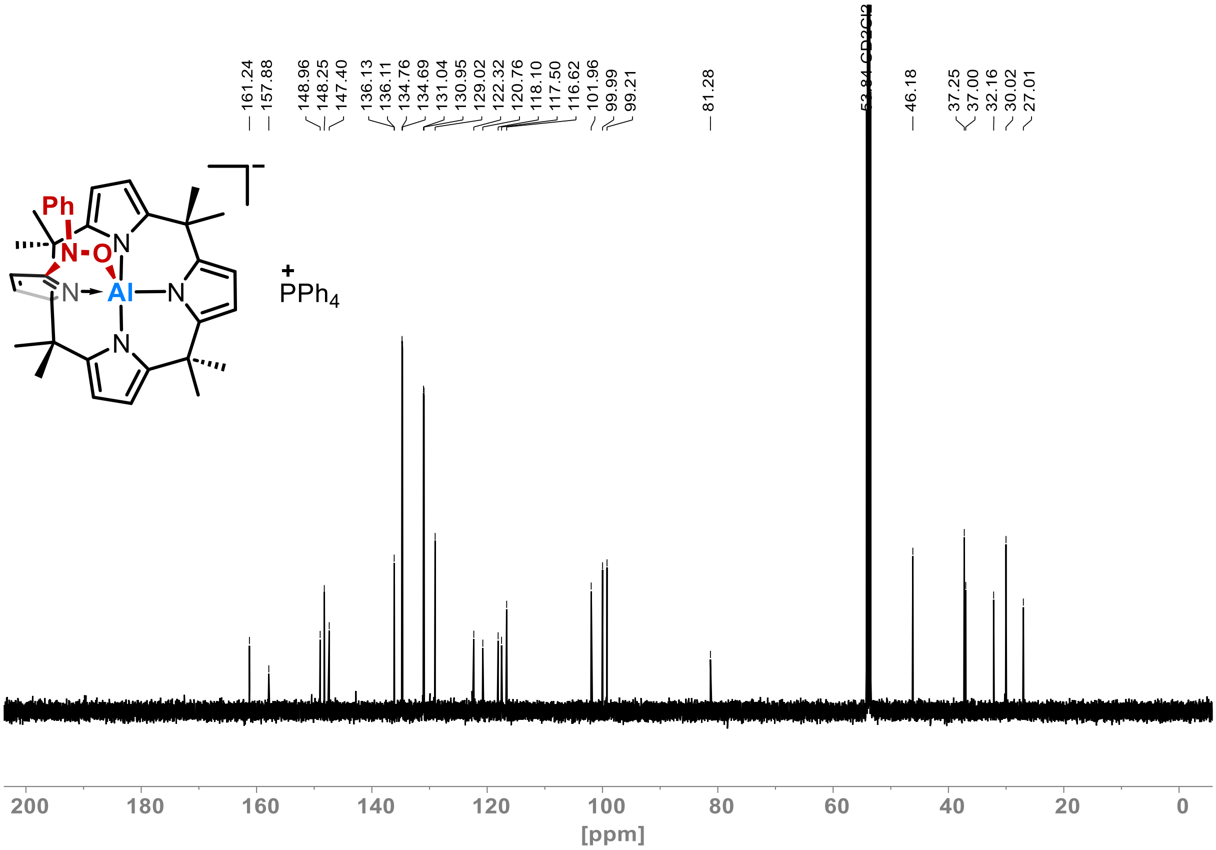

Figure S16. $^{13}C\{^{1}H\}$ NMR spectrum (151 MHz, CD_2Cl_2, 295 K) of $[PPh_4][^{Me}\mathbf{2^*}\text{-PhNO}]$.

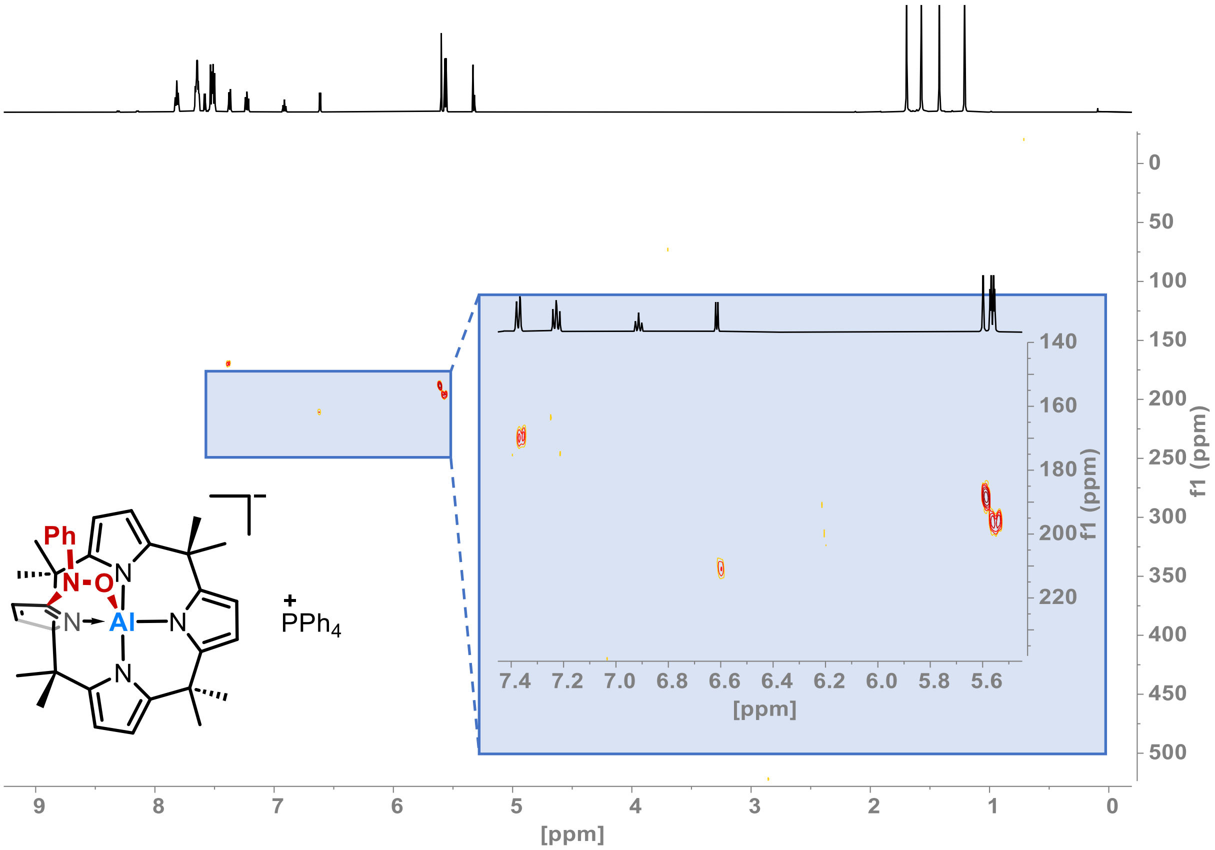

Figure S17. $^{1}H,^{15}N$ HMBC NMR spectrum (600 MHz, 61 MHz, CD_2Cl_2, 295 K) of $[PPh_4][^{Me}\mathbf{2^*}\text{-PhNO}]$. The ^{15}N chemical shifts are plotted with respect to ammonia reference.

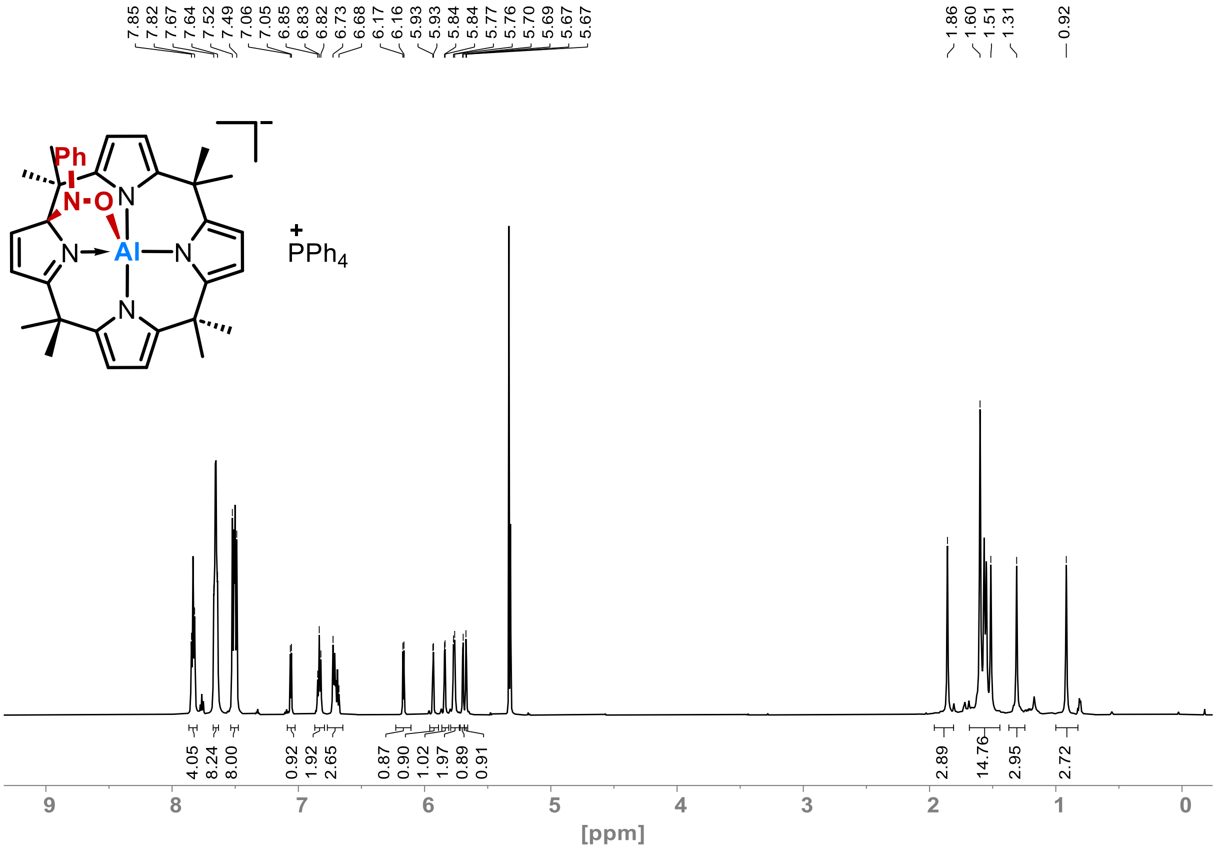

Figure S18. ^{1}H NMR spectrum (600 MHz, CD_2Cl_2, 225 K) of [PPh_4][^{Me}**1***-PhNO].

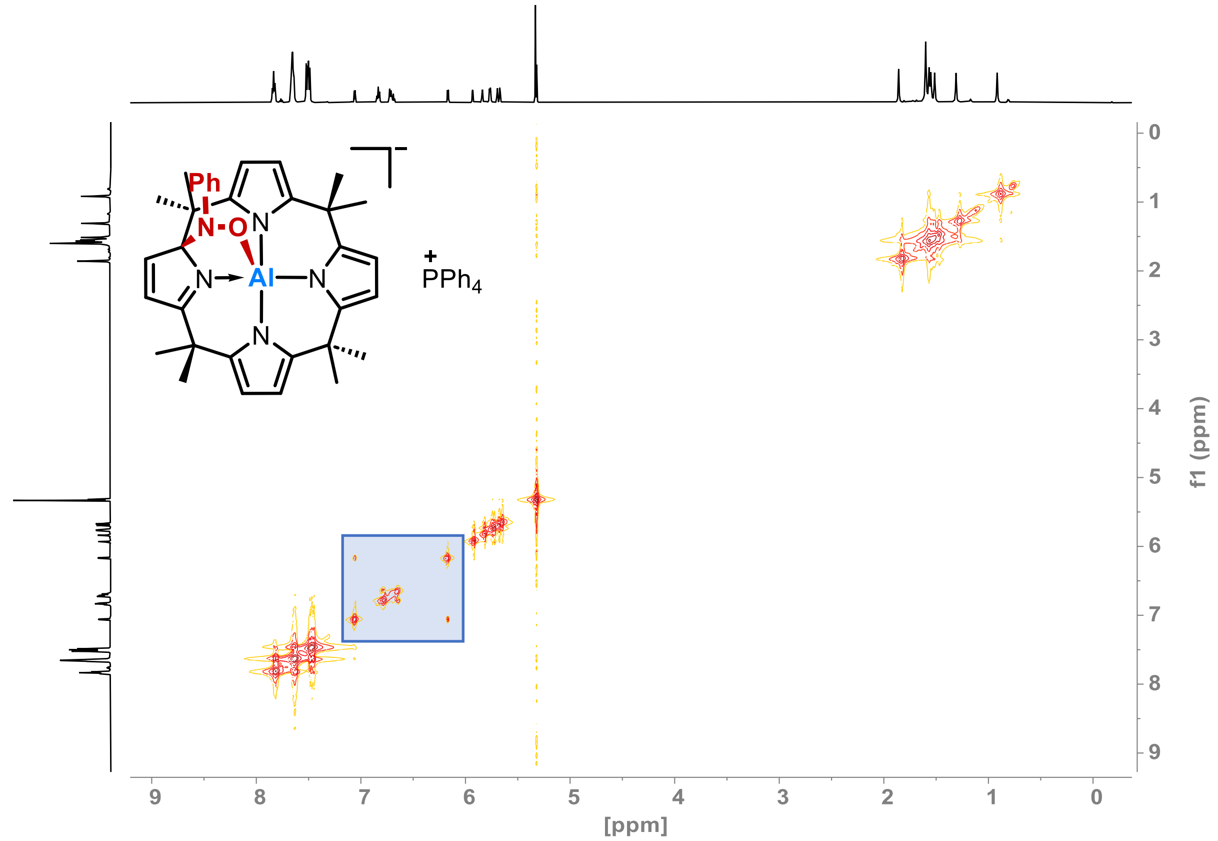

Figure S19. ^{1}H,^{1}H COSY NMR spectrum (600 MHz, 600 MHz, CD_2Cl_2, 205 K) of [PPh_4][^{Me}**1***-PhNO].

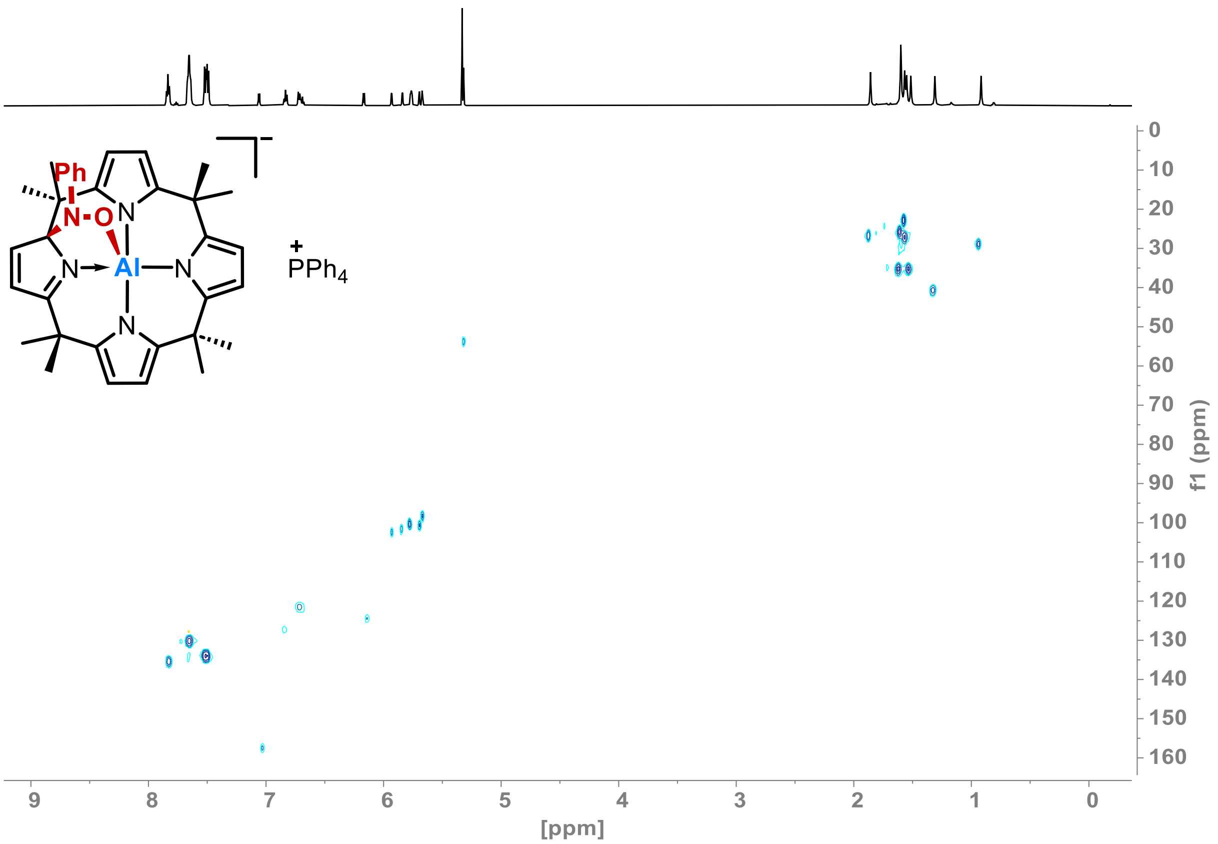

Figure S20. $^{1}H,^{13}C$ HSQC NMR spectrum (600 MHz, 151 MHz, CD_2Cl_2, 240 K) of [PPh_4][^{Me}**1***-PhNO].

8 FT-ATR-IR spectra

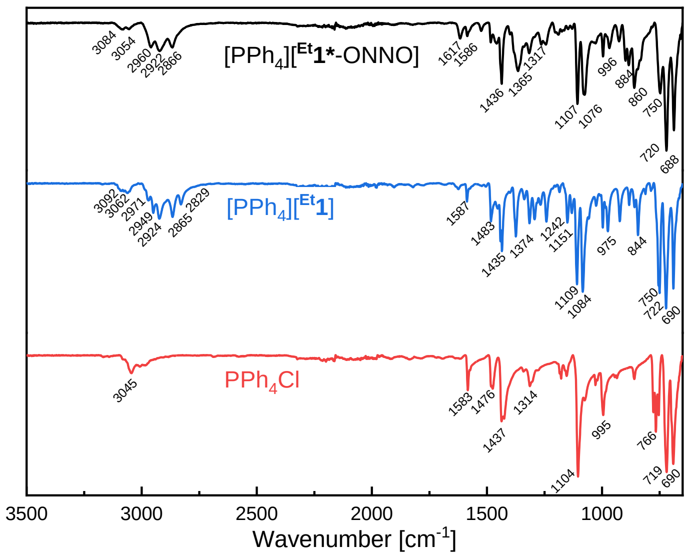

Figure S21. Solid-state FT-ATR-IR spectrum of [PPh_4][^{Et}**1***-ONNO], [PPh_4][^{Et}**1**] and PPh_4Cl. The spectrum was recorded at room temperature.

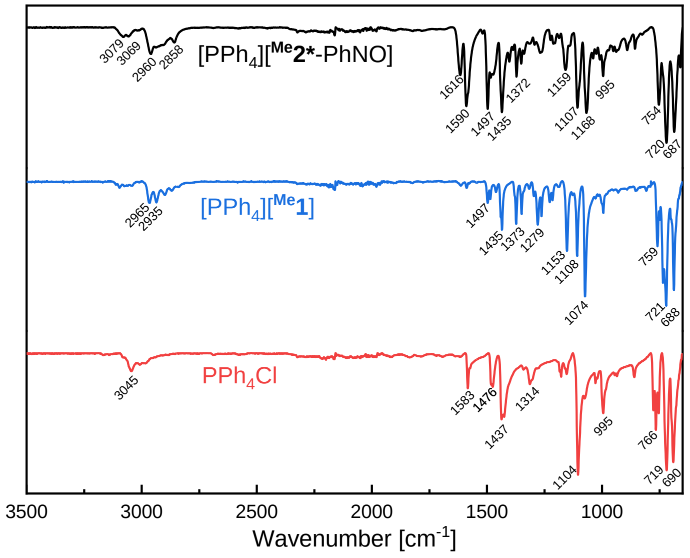

Figure S22. Solid-state FT-ATR-IR spectrum of $[PPh_4][^{Me}\mathbf{2}^{*}\text{-PhNO}]$, $[PPh_4][^{Me}\mathbf{1}]$ and PPh_4Cl. The spectrum was recorded at room temperature.

9 UV-Vis absorption spectra

$[PPh_4][^{Et}\mathbf{1}^{*}\text{-ONNO}]$ and $[PPh_4][^{Me}\mathbf{2}^{*}\text{-PhNO}]$ were analyzed by UV-Vis absorption spectroscopy (Figure S23).

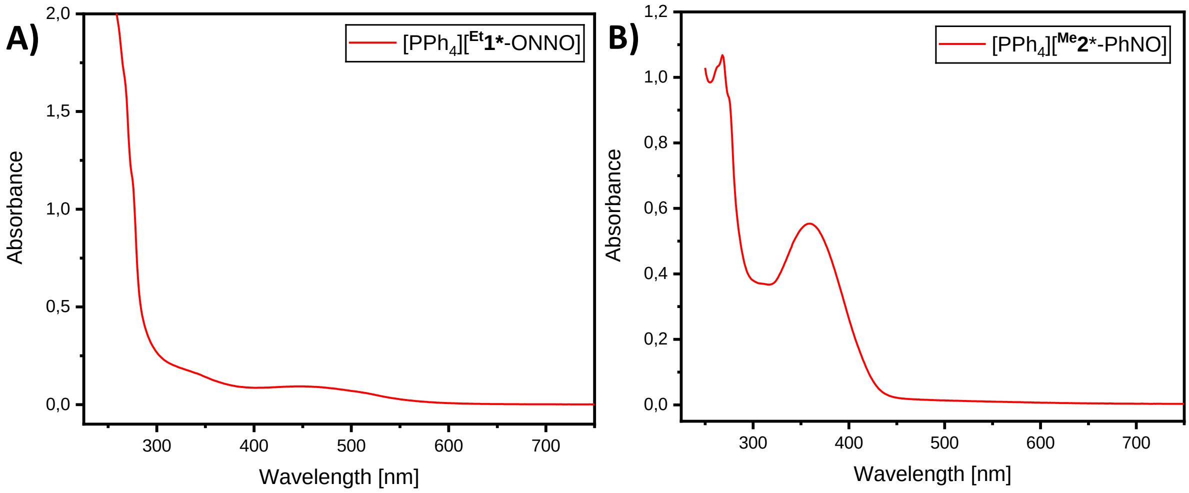

Figure S23. UV-Vis spectra of A) $[PPh_4][^{Et}\mathbf{1}^{*}\text{-ONNO}]$ and B) $[PPh_4][^{Me}\mathbf{2}^{*}\text{-PhNO}]$. Both spectra were obtained at room temperature with dichloromethane as solvent. The analyte concentration was in case of A) approximately $0.5 \cdot 10^{-3}$ mol L^{-1}, and in case of B) approximately $0.25 \cdot 10^{-3}$ mol L^{-1}.

10 Quantum chemical calculations

The quantum chemical calculations were carried out with Orca 5.0.4[12] using the computational resources of the bwForClusters JUSTUS2 at Ulm University within the Baden-Württemberg High Performance Computing program (bwHPC). Ball and stick representations were rendered with Chemcraft 1.8[13]. xyz Coordinates and computed energies are given in Chapter S12.

General remarks

Conformational spaces were initially explored with the conformer-rotamer ensemble sampling tool (CREST, version 2.11.2)[14] for the xtb program package (version 6.4.1)[15] using the following command line input. The lowest-energy conformer (according to the GFN2-xTB level of theory) was then reoptimized as described below.

```
crest [file_name].xyz --chrg [charge] --noreftopo > [file_name].out
```

Structure optimizations on the Kohn-Sham DFT level were done with the PBEh-3c composite method.[16] All equilibrium structures were confirmed to possess only positive Hessian matrix eigenvalues by analytic frequency calculations. The following Orca keyword line was used.

```
! PBEh-3c Opt Freq
```

Transition structures were optimized toward a single negative Hessian matrix eigenvalue with the `OptTS` keyword. It was ensured that the correct first-order saddle point on the potential energy surface was located by animation of the imaginary frequency in Chemcraft and by following the intrinsic reaction coordinate (IRC) by invoking the `IRC` keyword. Starting structures for transition structure optimizations were generated with the help of relaxed surface scans or with NEB calculations.

Final single point energies were calculated with the DSD-PBEB95 functional[17, 18], including the D3(BJ) correction[19, 20]. The def2-QZVPP set of basis functions was used.[21] The specific keyword line was:

```
! RI-DSD-PBEB95 D3BJ def2-QZVPP RIJCOSX AutoAux DefGrid3 VeryTightSCF
```

Enthalpies and Gibbs free energies at 298.15 K and 1 atm were calculated with the electronic energies from the double hybrid functional calculations combined with the corrections to enthalpies and Gibbs free energies[22], respectively, from the frequency calculations (see above).

Solvent influences (for dichloromethane) were included implicitly at T = 298.15 K with the conductor like screening model for real solvents (COSMO-RS)[23-25] as it is implemented[26] in the Amsterdam Modeling suite (AMS 2021.102)[27]. The AMS-preset parameters were used (BP86/TZP), except for the maximum number of iterations, which were set to zero (The structures as obtained from the PBEh-3c optimizations were used.).

Natural population analyses were done on the PBE0/def2-TZVPP level of theory interfacing Orca 5.0.4 with the NBO6 program.[28] The molecular structures optimized as described above (PBEh-3c) were used.

11 Reaction mechanism of the NO dimerization at [^{R}**1**]$^-$

To gain further insight of the NO Dimerization at [^{R}**1**]$^-$ quantum chemical calculations were conducted concerning a possible reaction mechanism (Figure S24). The addition of one nitrogen monoxide molecule features an energetic barrier of 82 kJ mol^{-1} leading to the by 5 kJ mol^{-1} endergonic 1,2-adduct [^{Me}**1***-NO]$^{\bullet-}$.

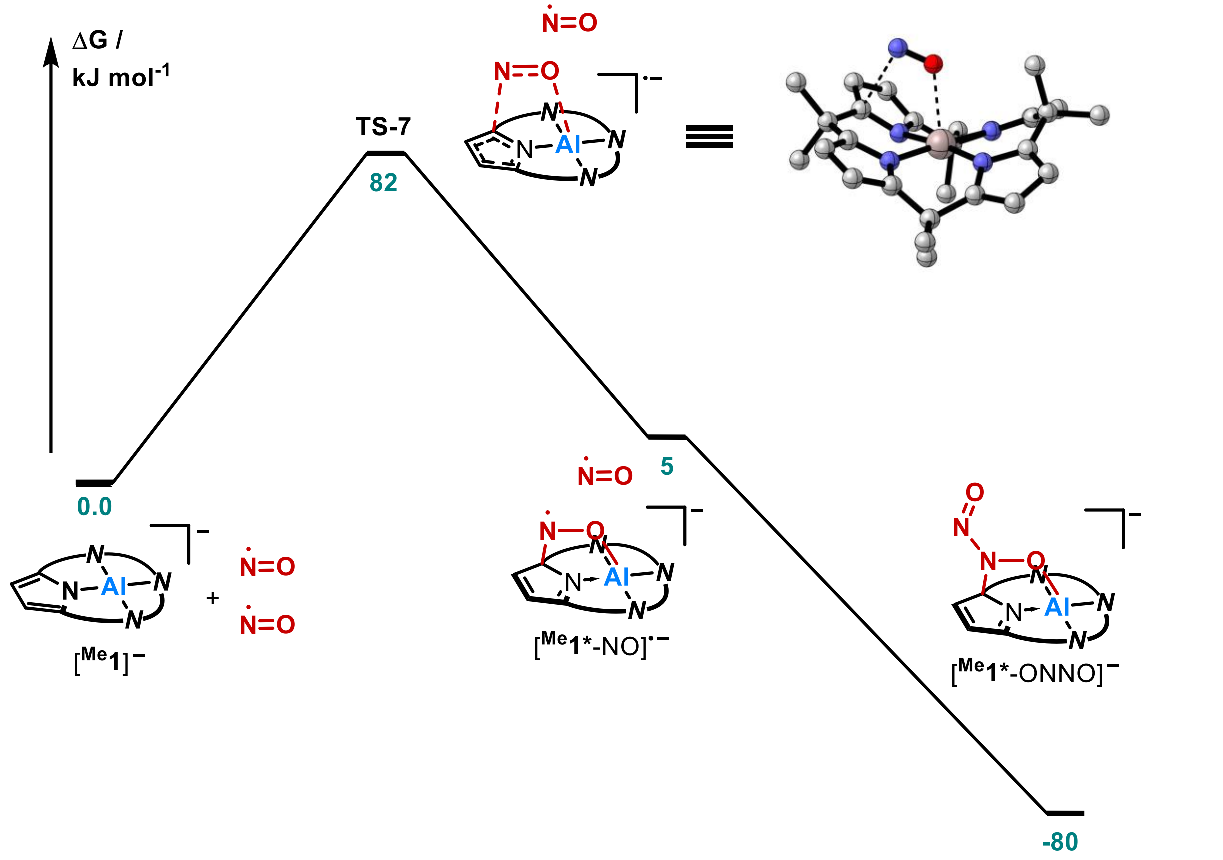

Figure S24. Computed Gibbs free reaction energies (kJ mol^{-1}) for the reaction of [^{Me}**1**]$^-$ with NO at the RI-DSD-PBEB95-D3(BJ)/def2-QZVPP, COSMO-RS(CH_2Cl_2)//PBEh-3c level of theory.

Other possible intermediates other than the 1,2-adduct [^{Me}**1***-NO]$^{\bullet-}$ were considered like the 1,1-adduct **[**^{Me}**1***-ON]$^{\bullet-}$ (Figure S25), but were higher in energy.

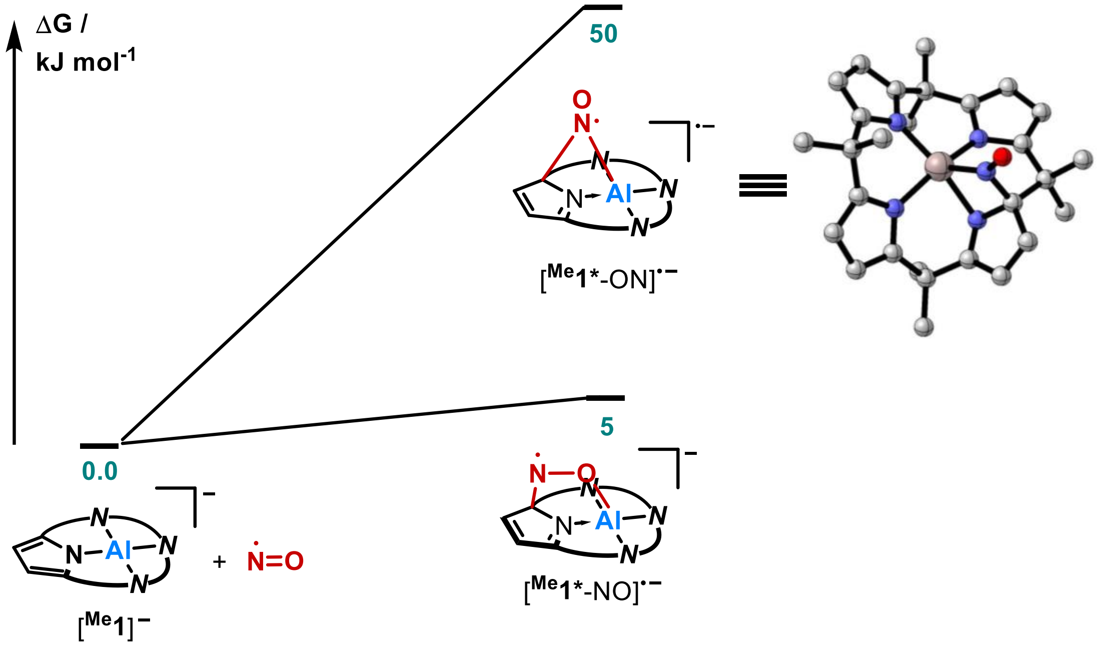

Figure S25. Computed Gibbs free reaction energies (kJ mol^{-1}) for the reaction of [Me**1**]$^-$ with NO yielding [Me**1***-NO]$^{\bullet-}$ or the 1,1-dduct [Me**1***-ON]$^{\bullet-}$ at the RI-DSD-PBEB95-D3(BJ)/def2-QZVPP, COSMO-RS(CH_2Cl_2)//PBEh-3c level of theory.

To calculate an energetic barrier for the addition of a second nitrogen monoxide molecule, we started from the optimized structure of [Me1*-ONNO]$^-$. The resulting scan displays that elongation of the N-N bond of the *N*-nitrosohydroxylaminato group leads to a constant incline in energy (Figure S26). This lacking of a saddle-point suggests that the addition of a second NO molecule does not feature an energetic barrier on the electronic potential energy surface.

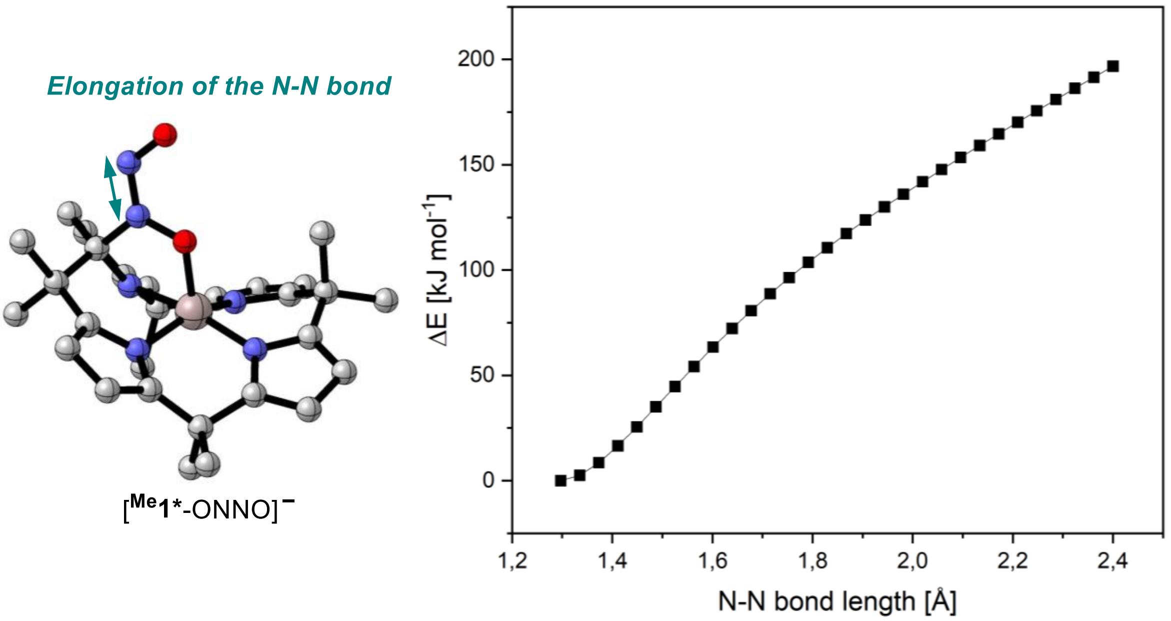

Figure S26. Potential energy surface scan elongating the N-N bond of [Me**1***-ONNO]$^-$ at the PBEh-3c level of theory.

12 Comparison of the reactions of [^{Me}**1**]$^{-}$ and [Al(pyrrolato)$_4$] with NO

To elucidate whether the structural constrain enables cooperative binding of NO by **[^{R}1]**$^{-}$, the thermodynamics of the reaction of the isolobal aluminum complex without structural constraint [Al(pyrrolato)$_4$]$^{-}$ with NO were calculated (Figure S27). Comparing both reactions displays that whilst the reaction turns from endergonic to exergonic for **[^{R}1]**$^{-}$ upon NO dimerization, in the case of [Al(pyrrolato)$_4$]$^{-}$, the reaction remains endergonic by 22 kJ mol^{-1}. These results highlight that the structural constraint imposed by the ligand scaffold depicts the key aspect for the observed NO dimerization.

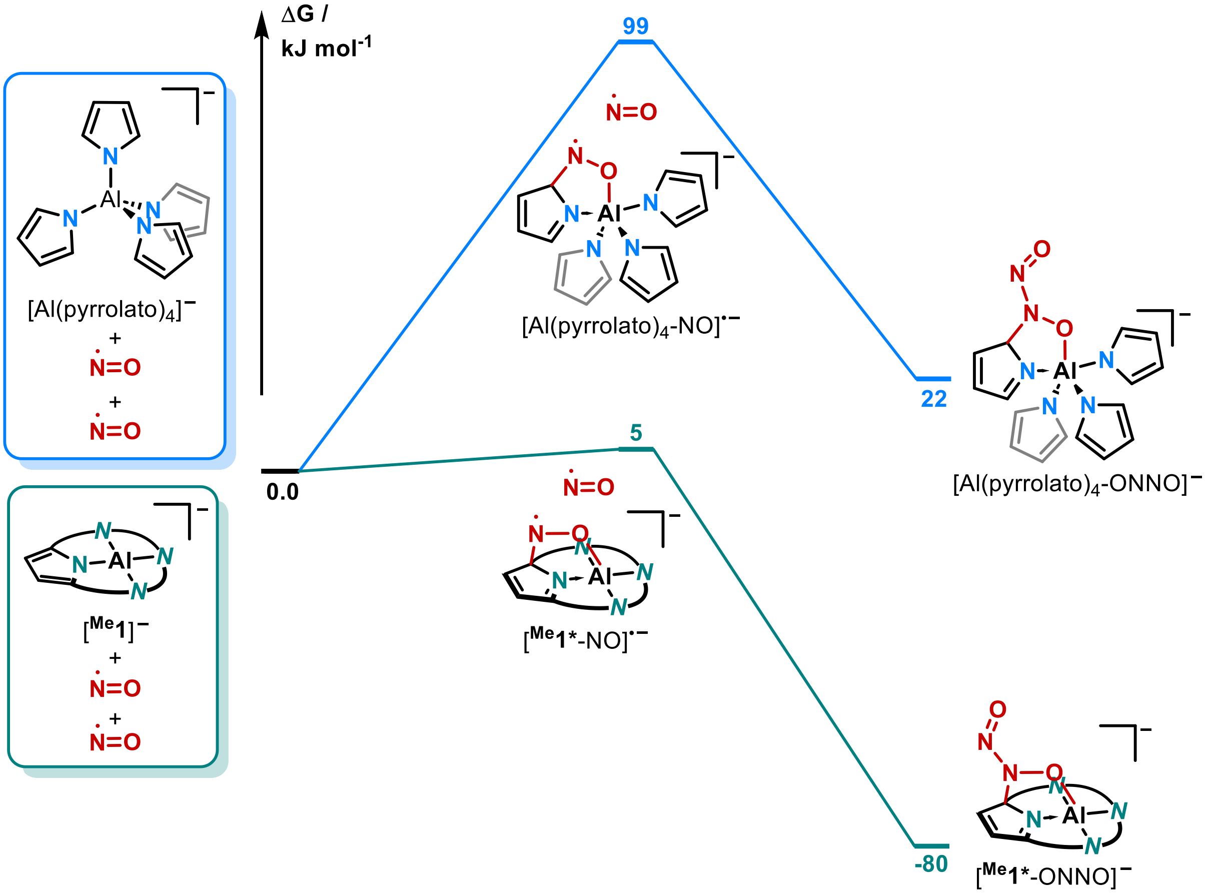

Figure S27. Computed Gibbs free reaction energies (kJ mol^{-1}) for the reactions of [Al(pyrrolato)$_4$] and [^{Me}**1**]$^{-}$ with NO at the RI-DSD-PBEB95-D3(BJ)/def2-QZVPP, COSMO-RS(CH_2Cl_2)//PBEh-3c level of theory.

13 Further quantum chemical data

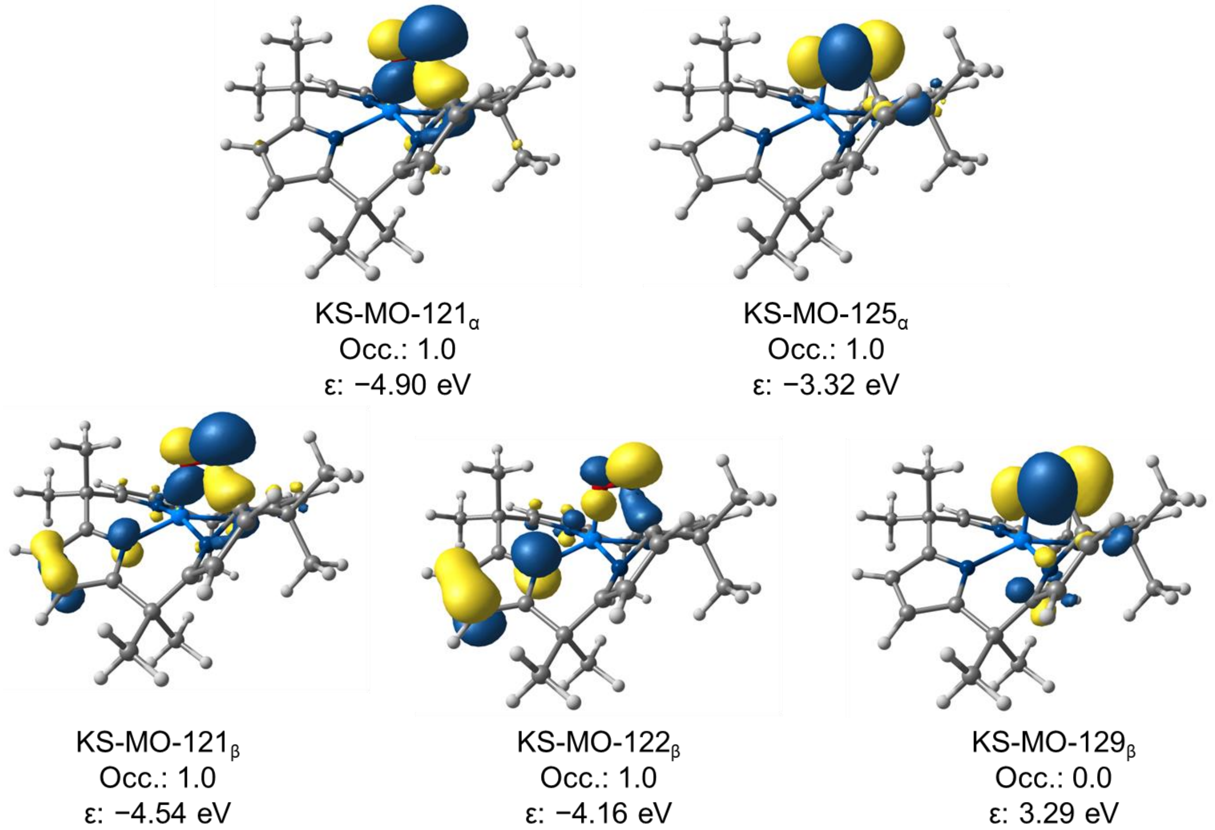

Figure S28. Isodensity surface plots of Kohn-Sham molecular orbitals of $[^{Me}\mathbf{1^*}\text{-NO}]^{\bullet-}$ with occupation numbers and orbital energies. The orbital indices for the set of occupied α orbitals go from 0 to 128, for the occupied β orbitals from 0 to 127. The data was obtained at the PBEh-3c level of theory.

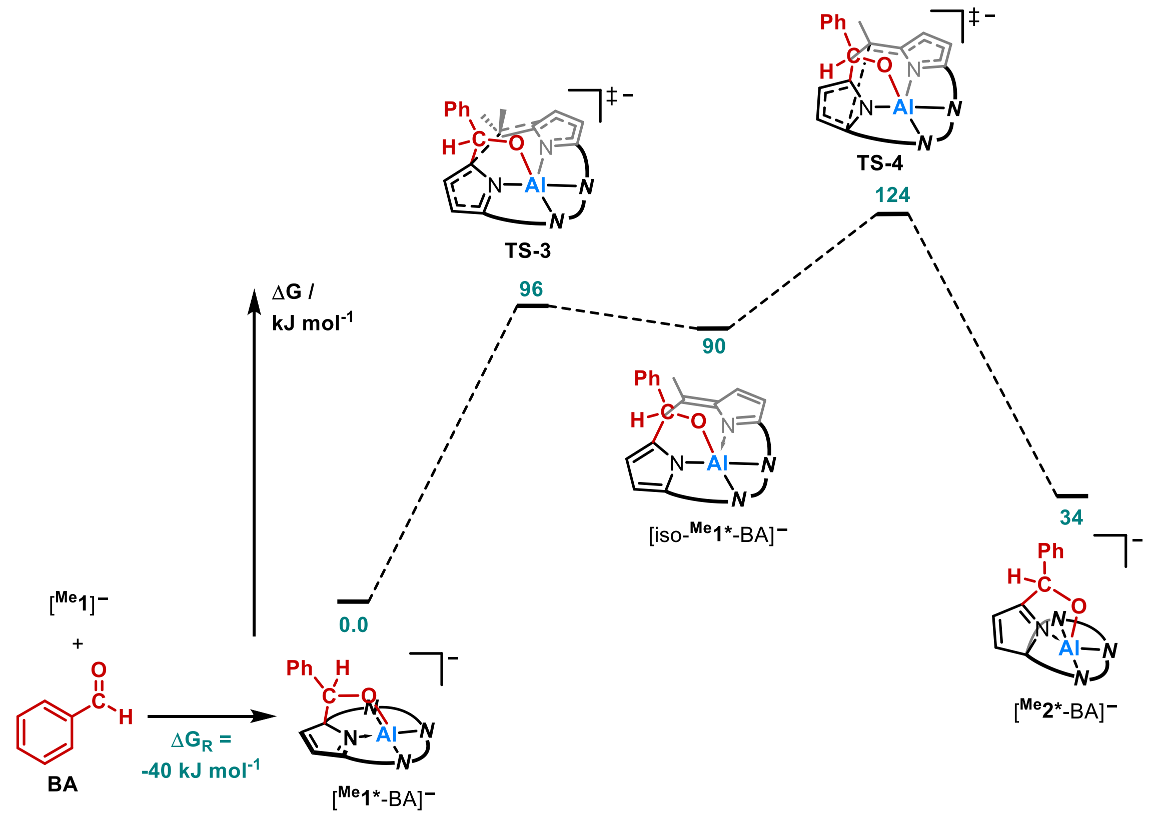

Figure S29. Rearrangement reaction as it was observed with nitrosobenzene as substrate simulated for [^{Me}**1***-BA]$^{-}$ at the RI-DSD-PBEB95-D3(BJ)/def2-QZVPP, COSMO-RS(CH_2Cl_2)//PBEh-3c computational level.

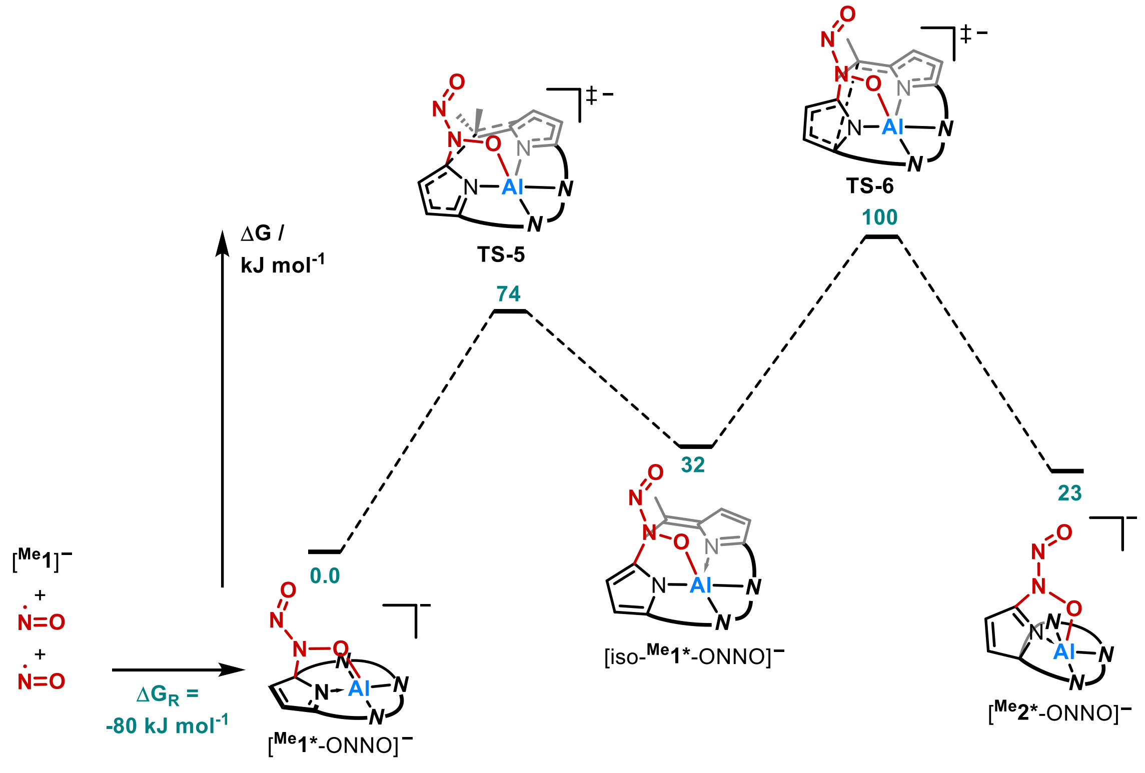

Figure S30. Rearrangement reaction as it was observed with nitrosobenzene as substrate simulated for [^{Me}**1***-ONNO]$^{-}$ at the RI-DSD-PBEB95-D3(BJ)/def2-QZVPP, COSMO-RS(CH_2Cl_2)//PBEh-3c computational level.

14 xyz coordinates

[^{Me}**1**]$^{-}$

xyz, charge: -1, multiplicity: 1

N	1.485293	1.187164	-0.004444
C	2.720965	0.936777	-0.545186
C	1.516372	2.447497	0.534800
C	3.528747	2.029366	-0.357010
H	4.551704	2.139517	-0.682331
C	2.759688	2.993979	0.342347
H	3.092568	3.967519	0.667468
N	1.187065	-1.485284	0.003748
C	0.936790	-2.720796	0.544914
C	2.447470	-1.516381	-0.535330
C	2.029382	-3.528601	0.356842
C	2.993970	-2.759655	-0.342675
H	3.967542	-3.092549	-0.667682
N	-1.485432	-1.187041	-0.003948
C	-2.721173	-0.936770	-0.544592
C	-1.516394	-2.447323	0.535422
C	-3.528879	-2.029384	-0.356224
H	-4.551868	-2.139613	-0.681418
C	-2.759698	-2.993889	0.343149
H	-3.092493	-3.967407	0.668420
N	-1.187190	1.485402	0.003705
C	-0.936826	2.720986	0.544663
C	-2.447714	1.516398	-0.535094
C	-2.029485	3.528736	0.356743
H	-2.139652	4.551642	0.682218
C	-2.994207	2.759679	-0.342468
H	-3.967858	3.092512	-0.667302
H	1.416690	4.693545	2.088394
C	0.504147	4.485983	1.527958
C	0.268001	2.302051	2.680844
C	0.337763	2.986339	1.298581
H	1.190028	2.467209	3.241656
H	-0.331593	4.881091	2.107232
H	0.131709	1.221297	2.599232
H	2.467398	-1.190062	-3.242229
C	2.302194	-0.268036	-2.681425
C	4.486039	-0.504190	-1.528365
C	2.986375	-0.337799	-1.299115
H	4.881193	0.331543	-2.107618
H	1.221438	-0.131747	-2.599899
H	4.693645	-1.416745	-2.088765
H	-1.189616	-2.466531	3.242225
C	-0.267673	-2.301498	2.681237
C	-0.503997	-4.485633	1.528771
C	-0.337663	-2.986027	1.299109
H	-1.416438	-4.693104	2.089406
H	-0.131360	-1.220764	2.599414
H	0.331853	-4.880632	2.107963
C	-2.986729	0.337711	-1.298634
C	-2.302774	0.267797	-2.681050
C	-4.486433	0.504048	-1.527659
H	-1.222000	0.131551	-2.599686
H	-4.694146	1.416527	-2.088143
H	-2.688238	-0.575107	-3.257880
H	-5.027559	0.551071	-0.582679

H	-2.468091	1.189754	-3.241932
H	-4.881670	-0.331764	-2.106741
H	-0.574824	2.687395	3.257870
H	0.551081	5.027345	0.583108
H	5.027317	-0.551099	-0.583466
H	2.687588	0.574788	-3.258417
H	-0.551083	-5.027149	0.584018
H	0.575232	-2.686761	3.258201
H	2.139593	-4.551469	0.682422
Al	-0.000065	0.000057	-0.000523

Total correction:	1547.9441 kJ/mol
Final single point energy:	-4059379.2198 kJ/mol
Final Gibbs free energy:	-4058041.14 kJ/mol
Gibbs free energy solvent correction:	-203.547 kJ/mol

[^{Me}**1***-BA]$^{-}$

xyz, charge: -1, multiplicity: 1

N	0.913735	-0.795847	0.933710
C	1.711669	-0.368496	1.835842
C	1.557013	-1.756070	0.083488
C	3.003526	-1.041380	1.734511
H	3.861195	-0.861838	2.363681
C	2.922743	-1.862866	0.679083
H	3.710283	-2.499457	0.305059
N	-0.090350	1.663566	0.824924
C	-0.368823	2.894421	0.294767
C	0.761916	1.860761	1.880165
C	0.307670	3.864759	1.003751
C	1.030376	3.203028	2.019733
H	1.658524	3.665577	2.765827
N	-2.304537	0.829099	-0.521122
C	-3.554785	0.274998	-0.565554
C	-2.419624	2.143555	-0.894218
C	-4.457750	1.219430	-0.993551
H	-5.521911	1.087093	-1.115298
C	-3.731323	2.413745	-1.204740
H	-4.135288	3.358184	-1.534997
N	-1.446851	-1.796850	0.099337
C	-0.696252	-2.904481	-0.227216
C	-2.737287	-2.069559	-0.259392
C	-1.511161	-3.863615	-0.783006
H	-1.216792	-4.844042	-1.126518
C	-2.819310	-3.332490	-0.798047
H	-3.705770	-3.821832	-1.169935
H	0.364513	-2.938171	2.307884
C	0.790866	-3.644598	1.596621
C	1.411752	-4.140321	-0.749825
C	0.753120	-3.095981	0.156882
H	2.464668	-4.280967	-0.497054
H	1.801745	-3.900556	1.923241
H	1.341213	-3.879731	-1.804593
H	0.454715	-0.655107	4.252261
C	0.117778	0.184020	3.637576
C	2.400482	1.188879	3.650991
C	1.255368	0.728495	2.753373
H	3.237135	1.575010	3.068136
H	-0.237900	0.976536	4.295989

H	2.760256	0.367889	4.274536
H	-1.028565	3.062139	-3.092302
C	-0.408529	2.865713	-2.214952
C	-1.745098	4.524339	-0.962296
C	-1.237497	3.080026	-0.930329
H	-0.912577	5.225116	-1.040916
H	-0.026035	1.851737	-2.302460
H	-2.318295	4.768590	-0.068117
C	-3.844924	-1.088575	0.008446
C	-4.022828	-0.946478	1.535757
C	-5.157167	-1.623000	-0.564740
H	-4.250349	-1.914068	1.990040
H	-5.399946	-2.593417	-0.128841
H	-3.117794	-0.561687	2.006848
H	-5.099081	-1.733866	-1.647430
H	-4.832762	-0.250046	1.760827
H	-5.981212	-0.946756	-0.335322
H	0.925728	-5.107226	-0.623379
H	0.181194	-4.546830	1.639253
H	2.064105	1.981757	4.318170
H	-0.727921	-0.158932	3.042746
H	-2.381101	4.685361	-1.833461
H	0.445306	3.547920	-2.233288
H	0.288357	4.928097	0.823546
Al	-0.661438	-0.012624	-0.043160
C	1.503766	-1.055347	-1.356264
O	0.369146	-0.310021	-1.481827
C	2.764971	-0.236091	-1.566160
C	2.851492	1.086689	-1.143338
C	3.874430	-0.813973	-2.175439
C	4.033093	1.796332	-1.299700
C	5.054435	-0.104103	-2.340810
C	5.139204	1.206859	-1.895427
H	1.997608	1.573285	-0.691561
H	3.810221	-1.836464	-2.534272
H	4.081297	2.821919	-0.955995
H	5.903200	-0.570689	-2.826000
H	6.056580	1.768176	-2.022516
H	1.524627	-1.870382	-2.096359

Total correction: 1872.8719 kJ/mol
Final single point energy: -4965740.715 kJ/mol
Final Gibbs free energy: -4964106.9765 kJ/mol
Gibbs free energy solvent correction: -223.4949 kJ/mol

[^{Me}**2***-BA]$^{-}$

xyz, charge: -1, multiplicity: 1

N	0.233556	-1.452523	-0.389837
C	-0.199080	-2.513307	0.450762
C	1.290565	-1.716713	-1.035825
C	0.878839	-3.540353	0.197960
H	0.912954	-4.520301	0.651325
C	1.761938	-3.053783	-0.688100
H	2.645510	-3.537259	-1.076224
N	0.330371	0.406110	1.353767
C	1.024432	1.464862	1.848178
C	0.669413	-0.713477	2.066515
C	1.842923	1.022285	2.871811

C	1.609717	-0.369441	3.013842
H	2.048302	-1.017996	3.757994
N	-1.047457	2.148296	-0.221072
C	-2.313226	2.557893	-0.523273
C	-0.508076	3.046993	0.662051
C	-2.574950	3.742668	0.124402
H	-3.491339	4.311926	0.089846
C	-1.419927	4.054442	0.882420
H	-1.293060	4.908888	1.529224
N	-2.136668	-0.447618	-0.696170
C	-2.520079	-1.722324	-0.319394
C	-3.275515	0.286744	-0.883939
C	-3.893463	-1.772179	-0.256902
H	-4.494489	-2.628405	0.010408
C	-4.377094	-0.493793	-0.621565
H	-5.411462	-0.192939	-0.680570
H	-1.543951	-4.940163	0.583202
C	-2.224659	-4.086453	0.556046
C	-1.363711	-3.438352	-1.640711
C	-1.585666	-2.918189	-0.200967
H	-0.740520	-4.334394	-1.676400
H	-3.125428	-4.416890	0.038921
H	-0.928858	-2.684829	-2.294669
H	-2.035483	-0.741911	1.912951
C	-1.567468	-1.519340	2.507067
C	0.335345	-3.042206	2.906832
C	-0.192887	-1.960734	1.962709
H	1.382451	-3.280708	2.717124
H	-2.281671	-2.333718	2.618563
H	-0.249948	-3.961063	2.827271
H	2.949609	3.016966	0.642727
C	1.952868	3.142516	0.212634
C	1.057321	3.875151	2.401945
C	0.866332	2.861905	1.269704
H	0.325338	3.726556	3.195355
H	1.860240	4.168620	-0.150803
H	0.959953	4.894733	2.025313
C	-3.207519	1.710876	-1.395489
C	-4.611705	2.309949	-1.418273
C	-2.673982	1.700330	-2.841784
H	-5.269155	1.730669	-2.068659
H	-1.659810	1.306571	-2.905833
H	-5.049174	2.333712	-0.420062
H	-2.653961	2.716972	-3.239974
H	-4.581442	3.331012	-1.800646
H	-3.311268	1.084943	-3.481338
H	-2.338152	-3.690121	-2.058965
H	-2.503935	-3.846165	1.576934
H	0.257865	-2.698033	3.938607
H	-1.393585	-1.100698	3.499954
H	2.054777	3.784011	2.834331
H	1.874986	2.478453	-0.645437
H	2.513164	1.622560	3.468245
Al	-0.401711	0.383744	-0.435170
C	1.745898	-0.574074	-1.914300
O	0.895648	0.488890	-1.718411
C	3.200218	-0.272372	-1.597301
C	3.587403	0.043802	-0.297051
C	4.167729	-0.324989	-2.591604
C	4.916665	0.303279	-0.008099

C	5.501924	-0.067540	-2.303700
C	5.879453	0.247014	-1.007988
H	2.856765	0.094060	0.501331
H	3.872824	-0.561983	-3.607805
H	5.195295	0.549683	1.008371
H	6.242536	-0.107802	-3.092822
H	6.917869	0.449913	-0.777367
H	1.712140	-0.933947	-2.960571

Total correction:	1873.4747 kJ/mol
Final single point energy:	-4965713.0713 kJ/mol
Final Gibbs free energy:	-4964075.6343 kJ/mol
Gibbs free energy solvent correction:	-220.351 kJ/mol

[iso-^{Me}**1***-BA]$^{-}$

xyz, charge: -1, multiplicity: 1

N	-0.264100	-1.621855	-0.641935
C	-0.918047	-2.728765	-0.244106
C	1.004526	-1.930813	-0.973930
C	-0.024529	-3.793642	-0.272587
H	-0.241895	-4.829805	-0.052892
C	1.210318	-3.282423	-0.747310
H	2.107181	-3.851594	-0.942924
N	-2.446082	-0.132596	-0.513896
C	-3.423023	0.827139	-0.492680
C	-3.030822	-1.323396	-0.114761
C	-4.602610	0.284133	-0.046714
C	-4.348676	-1.085255	0.201502
H	-5.071190	-1.818772	0.527136
N	-0.823294	2.142463	-0.137415
C	0.026163	2.954888	0.561458
C	-1.982239	2.835100	-0.329465
C	-0.582628	4.168153	0.792659
H	-0.168728	5.012708	1.321997
C	-1.869325	4.092930	0.217107
H	-2.624031	4.863670	0.229833
N	0.463691	0.277560	1.420313
C	0.909772	-0.907651	2.017059
C	1.491671	1.093743	1.367093
C	2.333625	-0.807324	2.202319
H	2.972759	-1.561923	2.632332
C	2.687722	0.448026	1.843574
H	3.672732	0.885499	1.875777
H	0.437153	-3.042460	4.305763
C	0.586267	-3.060029	3.222145
C	-1.358334	-1.495762	2.842244
C	0.077335	-1.804558	2.596432
H	-1.960546	-2.399255	2.885984
H	1.635245	-3.251160	3.013078
H	-1.801242	-0.817183	2.119607
H	-2.342133	-2.411556	-2.476509
C	-2.776461	-3.116275	-1.768336
C	-3.054429	-3.740588	0.611712
C	-2.423208	-2.701529	-0.315185
H	-2.942512	-3.482930	1.662293
H	-2.382746	-4.110152	-1.993277
H	-2.594528	-4.717842	0.453094
H	-5.247963	2.646385	-1.375542

C	-4.394033	3.081354	-0.853921
C	-2.861454	2.114469	-2.544613
C	-3.154184	2.208650	-1.033291
H	-1.969597	1.523298	-2.752711
H	-4.228024	4.077187	-1.267994
H	-2.697082	3.111642	-2.958914
C	1.413876	2.542235	0.967674
C	2.426203	2.868426	-0.143654
C	1.821965	3.324983	2.232852
H	2.459094	3.950265	-0.281437
H	2.834781	3.071801	2.550412
H	3.433932	2.521139	0.095423
H	1.139293	3.127501	3.058811
H	2.121212	2.407357	-1.076795
H	1.805007	4.395390	2.028220
H	-1.435245	-1.014420	3.823586
H	0.015886	-3.903060	2.830927
H	-4.121086	-3.852365	0.412971
H	-3.858951	-3.119417	-1.912276
H	-3.697753	1.649824	-3.071628
H	-4.654004	3.190203	0.199244
H	-5.544105	0.796515	0.077451
Al	-0.544075	0.258854	-0.441952
C	1.659547	-0.788478	-1.714513
O	0.919836	0.372909	-1.501987
C	3.144344	-0.571119	-1.468927
C	3.783824	0.457915	-2.158773
C	3.913581	-1.370508	-0.634520
C	5.140037	0.692076	-2.007008
C	5.276967	-1.148058	-0.484071
C	5.897228	-0.113297	-1.164867
H	3.199188	1.090961	-2.815042
H	3.435491	-2.167265	-0.080945
H	5.609655	1.504723	-2.547725
H	5.854491	-1.786040	0.174387
H	6.959052	0.063986	-1.045960
H	1.601592	-1.057982	-2.786895

Total correction:	1869.2665 kJ/mol
Final single point energy:	-4965649.0472 kJ/mol
Final Gibbs free energy:	-4964028.8936 kJ/mol
Gibbs free energy solvent correction:	-211.7224 kJ/mol

[^{Me}**1***-NO]$^{\bullet-}$

xyz, charge: -1, multiplicity: 2

N	1.552824	0.906286	0.072079
C	1.810242	2.094195	0.465235
C	2.467968	0.493509	-0.955122
C	2.979760	2.626573	-0.234759
H	3.397298	3.612371	-0.100512
C	3.363622	1.684767	-1.104818
H	4.170610	1.751404	-1.818926
N	-1.000340	1.625072	0.182179
C	-2.271555	1.937417	-0.216551
C	-0.494383	2.728318	0.818655
C	-2.572945	3.224999	0.171233
C	-1.434380	3.732153	0.832340
H	-1.328760	4.714534	1.267276

N	-1.821450	-0.959402	-0.128164
C	-2.076027	-2.233148	0.307787
C	-3.010314	-0.416135	-0.543997
C	-3.410784	-2.512036	0.138395
H	-3.917576	-3.427881	0.400566
C	-4.007125	-1.352548	-0.409124
H	-5.049861	-1.227251	-0.656482
N	0.882590	-1.672027	0.240015
C	2.174760	-1.890692	-0.185762
C	0.333132	-2.896976	0.499985
C	2.431546	-3.241903	-0.186287
H	3.356182	-3.724236	-0.465778
C	1.257268	-3.885977	0.257898
H	1.115696	-4.948901	0.375256
H	4.796581	0.215958	0.603462
C	4.016559	-0.530247	0.776312
C	4.136801	-1.265413	-1.601663
C	3.188508	-0.808802	-0.488658
H	4.914353	-0.520340	-1.787006
H	4.499245	-1.453905	1.096049
H	3.597628	-1.444906	-2.529226
H	1.919615	1.946034	3.193348
C	0.915447	1.965238	2.761122
C	1.326365	4.192572	1.716100
C	0.884703	2.757572	1.440123
H	0.658560	4.660190	2.438578
H	0.237516	2.430972	3.476444
H	1.310472	4.797806	0.809508
H	-1.663082	0.786809	-2.698056
C	-2.695593	1.085353	-2.533572
C	-4.579164	1.445757	-0.974580
C	-3.116209	1.000451	-1.050466
H	-4.949065	1.425279	0.050121
H	-2.807381	2.109220	-2.897960
H	-5.207668	0.793297	-1.581567
C	-1.051406	-3.033225	1.070142
C	-1.030765	-2.532105	2.530963
C	-1.458236	-4.506193	1.087022
H	-2.015014	-2.649297	2.989033
H	-1.491845	-4.916872	0.078163
H	-0.297715	-3.090319	3.118093
H	-2.443513	-4.629247	1.537723
H	-0.766580	-1.475390	2.585152
H	-0.756361	-5.095619	1.678901
H	4.642581	-2.185983	-1.312942
H	3.392046	-0.191046	1.602706
H	2.333352	4.217635	2.137852
H	0.594666	0.933628	2.621193
H	-4.697653	2.457283	-1.365919
H	-3.329885	0.430296	-3.134781
H	-3.495902	3.753323	-0.007463
Al	-0.140017	-0.084969	-0.241806
N	1.728826	0.274410	-2.218677
O	0.455854	0.039475	-1.989508

Total correction:	1574.8597 kJ/mol
Final single point energy:	-4400158.0297 kJ/mol
Final Gibbs free energy:	-4398801.0342 kJ/mol
Gibbs free energy solvent correction:	-221.516 kJ/mol

[^{Me}**1***-ONNO]$^{-}$

xyz, charge: -1, multiplicity: 1

N	1.460832	0.755296	0.497417
C	1.750884	1.893252	0.998686
C	2.503150	0.306389	-0.386709
C	3.064905	2.342026	0.534272
H	3.542324	3.270137	0.807192
C	3.514487	1.419075	-0.322701
H	4.444918	1.431182	-0.866422
N	-1.020200	1.650784	0.288865
C	-2.194529	2.076786	-0.271448
C	-0.524483	2.693334	1.029244
C	-2.441742	3.377077	0.112401
C	-1.372935	3.772069	0.944548
H	-1.254218	4.731161	1.425713
N	-1.994400	-0.849909	-0.205529
C	-2.408257	-2.093272	0.193631
C	-3.076791	-0.200608	-0.741624
C	-3.736032	-2.250956	-0.122003
H	-4.349884	-3.115100	0.079859
C	-4.162518	-1.042356	-0.721091
H	-5.156917	-0.822460	-1.076816
N	0.575064	-1.756989	0.536692
C	1.882076	-2.071361	0.232880
C	-0.099245	-2.938510	0.674553
C	2.024640	-3.438383	0.191065
H	2.929700	-3.988602	-0.017484
C	0.760064	-3.993736	0.479785
H	0.516567	-5.043339	0.526509
H	2.985182	-0.515828	2.223309
C	3.683161	-0.904828	1.482165
C	4.065741	-1.590118	-0.883539
C	3.016818	-1.076446	0.105951
H	4.504357	-2.517447	-0.516358
H	4.556747	-0.248544	1.450377
H	4.884441	-0.877859	-1.003691
H	-0.258579	2.212075	3.737495
C	0.498121	1.729754	3.118781
C	1.232597	3.961211	2.286186
C	0.736763	2.583540	1.858553
H	2.159481	3.885284	2.859302
H	0.138295	0.732238	2.870371
H	0.495495	4.442662	2.927781
H	-2.974310	0.693302	-3.336699
C	-2.378648	1.291224	-2.643777
C	-4.414167	1.792034	-1.336886
C	-2.996464	1.224118	-1.230978
H	-5.008345	1.201984	-2.035678
H	-1.357219	0.920915	-2.682923
H	-4.392481	2.813073	-1.719878
C	-1.550490	-2.973541	1.067117
C	-1.678076	-2.483437	2.526513
C	-2.063679	-4.411711	1.010684
H	-1.353336	-1.447267	2.629756
H	-2.009432	-4.809016	-0.002484
H	-2.717472	-2.533981	2.856572
H	-3.101248	-4.464547	1.341541
H	-1.064635	-3.093700	3.193708

H	-1.480414	-5.057320	1.669253
H	3.637645	-1.792175	-1.863060
H	4.011081	-1.882127	1.834045
H	1.403789	4.615202	1.430651
H	1.411807	1.620578	3.709105
H	-4.921007	1.794661	-0.371839
H	-2.368237	2.324764	-2.997522
H	-3.284232	3.985861	-0.174377
Al	-0.255863	-0.113317	-0.081411
N	1.849204	0.266983	-1.687356
O	0.557660	-0.068393	-1.733954
N	2.533624	0.665715	-2.714616
O	1.960459	0.686230	-3.782714

Total correction: 1607.9748 kJ/mol
Final single point energy: -4741044.4603 kJ/mol
Final Gibbs free energy: -4739662.1133 kJ/mol
Gibbs free energy solvent correction: -228.5341 kJ/mol

[^{Me}**2***-ONNO]$^-$

xyz, charge: -1, multiplicity: 1

N	-1.530323	0.294352	-0.518008
C	-2.507728	-0.074319	0.439523
C	-1.959275	1.140000	-1.361191
C	-3.697701	0.724482	-0.048482
H	-4.680175	0.699502	0.399643
C	-3.355933	1.464890	-1.115465
H	-3.964246	2.144823	-1.688890
N	-0.102726	-1.825637	-0.238760
C	0.838392	-2.815604	-0.167887
C	-1.288035	-2.328131	0.271639
C	0.277867	-3.939629	0.390975
C	-1.073384	-3.625876	0.673956
H	-1.800784	-4.291426	1.114114
N	2.217293	-0.161317	-0.233103
C	2.993227	0.762238	0.423248
C	2.871885	-1.361691	-0.212763
C	4.165083	0.155192	0.810249
H	4.980971	0.614526	1.346297
C	4.088676	-1.200089	0.404722
H	4.832067	-1.962817	0.578509
N	0.238297	1.224909	1.046009
C	-0.928333	1.520713	1.702903
C	1.141833	2.209344	1.295078
C	-0.767731	2.714420	2.370162
H	-1.487433	3.198149	3.014386
C	0.552760	3.158906	2.106458
H	1.009638	4.063251	2.478625
H	-1.983018	-1.419036	3.079393
C	-1.311712	-0.626411	2.755633
C	-3.167893	0.996907	2.684689
C	-1.988252	0.447448	1.879080
H	-3.960867	0.252055	2.782858
H	-0.952850	-0.117690	3.651530
H	-3.590736	1.898255	2.239704
H	-4.628936	-1.741911	1.072793
C	-3.668634	-2.255845	1.155671
C	-3.194928	-1.887844	-1.217745

C	-2.644746	-1.631311	0.203859
H	-2.540731	-1.499876	-1.996192
H	-3.843482	-3.297351	0.885412
H	-4.195560	-1.474242	-1.365324
H	3.086386	-2.401060	-2.741691
C	2.097867	-2.512563	-2.291642
C	3.092935	-3.821889	-0.435968
C	2.220392	-2.610775	-0.757569
H	4.087014	-3.700937	-0.868550
H	1.500885	-1.656555	-2.606000
H	2.658559	-4.731170	-0.854330
C	2.531498	2.183019	0.679341
C	3.504828	2.855856	1.651758
C	2.551479	2.998357	-0.629302
H	3.213187	3.891567	1.830244
H	3.561403	3.012624	-1.045329
H	3.528600	2.337995	2.610136
H	2.241473	4.028659	-0.439425
H	4.514924	2.868974	1.238984
H	1.889117	2.585722	-1.386550
H	-2.838031	1.252284	3.692003
H	-0.448753	-1.089246	2.288386
H	-3.255434	-2.965573	-1.367673
H	-3.367102	-2.238188	2.197883
H	3.204333	-3.957526	0.639942
H	1.626127	-3.410349	-2.697290
H	0.768062	-4.882297	0.578033
Al	0.366064	0.026885	-0.439878
N	-1.004846	1.529346	-2.256126
O	0.200065	0.983045	-2.085994
N	-1.339650	2.384623	-3.199851
O	-0.469855	2.695667	-3.969105

Total correction:	1608.6137 kJ/mol
Final single point energy:	-4741039.5727 kJ/mol
Final Gibbs free energy:	-4739653.4024 kJ/mol
Gibbs free energy solvent correction:	-214.1973 kJ/mol

[iso-^{Me}**1***-ONNO]$^{-}$

xyz, charge: -1, multiplicity: 1

N	1.340468	0.860250	-0.851748
C	2.613099	0.913110	-0.411394
C	0.960271	2.085035	-1.232076
C	3.045884	2.230898	-0.502067
H	4.034932	2.603332	-0.275534
C	1.974991	3.000009	-1.031336
H	1.958621	4.054742	-1.249262
N	1.112693	-1.702739	-0.311184
C	0.745061	-3.003412	-0.082081
C	2.441175	-1.570613	0.060840
C	1.798681	-3.689191	0.468978
C	2.875051	-2.773747	0.565342
H	3.868532	-2.996196	0.925407
N	-1.677996	-1.324934	-0.126009
C	-2.833879	-0.845713	0.427530
C	-1.730706	-2.687859	-0.090192
C	-3.636999	-1.905769	0.781991
H	-4.611191	-1.855365	1.244081

C	-2.933681	-3.084425	0.445762
H	-3.266809	-4.097725	0.606855
N	-0.699136	0.897676	1.156648
C	0.133472	1.926992	1.617020
C	-1.883806	1.423296	0.928060
C	-0.596582	3.162920	1.520309
H	-0.226151	4.139923	1.786442
C	-1.856408	2.848098	1.133885
H	-2.687634	3.520450	0.994289
H	3.182494	2.609325	2.554699
C	2.138658	2.813142	2.792619
C	1.650865	0.333070	2.771211
C	1.286402	1.695040	2.289074
H	1.319486	0.241228	3.811961
H	2.065431	2.894617	3.881510
H	2.726849	0.180144	2.770418
H	3.051975	-0.793549	-2.442591
C	3.876634	-0.699196	-1.736896
C	4.551338	-0.297169	0.613366
C	3.340856	-0.400355	-0.313718
H	4.266146	-0.141121	1.652035
H	4.527990	0.106772	-2.082480
H	5.188338	0.535931	0.309961
H	-1.704420	-5.377641	-0.523982
C	-0.768164	-4.972777	-0.137323
C	-0.558752	-3.505236	-2.128397
C	-0.581032	-3.525035	-0.585074
H	-0.443038	-2.493383	-2.516735
H	-0.784082	-5.053603	0.949652
H	-1.491970	-3.907288	-2.528205
C	-3.120962	0.622049	0.621126
C	-3.899149	1.208695	-0.568904
C	-3.995445	0.790436	1.883863
H	-3.336358	1.142360	-1.493405
H	-4.227276	1.840853	2.067145
H	-4.829834	0.651139	-0.680693
H	-3.501884	0.384097	2.766264
H	-4.153181	2.259218	-0.410206
H	-4.942987	0.267701	1.753465
H	1.188110	-0.475755	2.214838
H	1.886162	3.773290	2.350781
H	5.160793	-1.200165	0.567534
H	4.436433	-1.636727	-1.744244
H	0.273787	-4.100996	-2.508840
H	0.040275	-5.599257	-0.516707
H	1.814568	-4.729690	0.753991
Al	-0.145540	-0.263518	-0.476012
N	-0.344055	2.082354	-1.728452
O	-0.951143	0.879233	-1.713324
N	-0.869637	3.166134	-2.189675
O	-2.000868	3.093764	-2.640234

Total correction: 1604.4086 kJ/mol
Final single point energy: -4741011.043 kJ/mol
Final Gibbs free energy: -4739640.6427 kJ/mol
Gibbs free energy solvent correction: -217.7677 kJ/mol

$[^{Me}\mathbf{1*}\text{-PhNO}]^{-}$

xyz, charge: -1, multiplicity: 1

N	0.886631	0.782750	-0.959119
C	1.651667	0.361830	-1.887332
C	1.574777	1.699735	-0.084239
C	2.959086	1.019555	-1.810890
H	3.788270	0.856652	-2.481266
C	2.935369	1.797996	-0.725108
H	3.743146	2.413017	-0.360483
N	-0.127625	-1.653742	-0.819990
C	-0.394158	-2.878646	-0.271270
C	0.695913	-1.859508	-1.895430
C	0.263803	-3.855666	-0.989398
C	0.960185	-3.203238	-2.029855
H	1.568434	-3.673009	-2.787829
N	-2.293030	-0.793327	0.578987
C	-3.539282	-0.228623	0.629749
C	-2.414476	-2.105799	0.956554
C	-4.443727	-1.165121	1.072186
H	-5.506075	-1.025558	1.201297
C	-3.724444	-2.364229	1.282286
H	-4.132496	-3.304198	1.620183
N	-1.424832	1.805059	-0.068268
C	-0.661789	2.910125	0.227441
C	-2.718290	2.109359	0.251827
C	-1.474991	3.909535	0.709576
H	-1.170005	4.898861	1.016569
C	-2.791425	3.399852	0.721373
H	-3.677076	3.920378	1.050346
H	0.554515	3.127989	-2.236917
C	0.958815	3.746161	-1.434524
C	1.473762	3.948360	0.998082
C	0.818195	3.061005	-0.062470
H	1.340723	3.531163	1.993179
H	1.993395	3.998982	-1.679907
H	1.036601	4.946178	0.979744
H	0.324631	0.645794	-4.273967
C	0.003201	-0.186937	-3.642527
C	2.281433	-1.201805	-3.720687
C	1.165154	-0.732772	-2.791711
H	3.133435	-1.587767	-3.160568
H	-0.376218	-0.982137	-4.284240
H	2.624527	-0.386511	-4.360788
H	-0.001569	-1.818686	2.323913
C	-0.385795	-2.832871	2.242880
C	-1.754951	-4.491003	1.024987
C	-1.238424	-3.050410	0.974480
H	-2.370877	-4.643534	1.911970
H	0.468678	-3.514611	2.250085
H	-0.925724	-5.197305	1.086399
C	-3.832665	1.124512	0.029487
C	-4.042050	0.942080	-1.489810
C	-5.131751	1.677804	0.615678
H	-4.276362	1.897989	-1.964716
H	-5.380917	2.637838	0.160958
H	-3.147111	0.542110	-1.968137
H	-5.050324	1.815713	1.693633
H	-4.857208	0.241580	-1.680800
H	-5.962917	0.999807	0.419541
H	2.544632	4.069750	0.812000
H	0.380571	4.669332	-1.422259

H	1.920568	-1.997684	-4.370923
H	-0.822510	0.164874	-3.025192
H	-2.351476	-4.734302	0.146017
H	-0.990412	-3.026024	3.131424
H	0.247815	-4.917550	-0.800639
Al	-0.658644	0.027953	0.056502
N	1.605031	1.045356	1.241459
O	0.425947	0.352114	1.476657
C	2.737602	0.210969	1.440632
C	3.880045	0.807345	1.971689
C	2.761855	-1.150612	1.145456
C	5.043857	0.077554	2.152027
C	3.927167	-1.876833	1.337435
C	5.075107	-1.271872	1.830578
H	3.834710	1.852304	2.253929
H	1.882944	-1.651623	0.765840
H	5.921092	0.559451	2.566375
H	3.931311	-2.930785	1.089467
H	5.978718	-1.849565	1.978674

Total correction: 1838.5084 kJ/mol
Final single point energy: -5007666.2274 kJ/mol
Final Gibbs free energy: -5006065.8922 kJ/mol
Gibbs free energy solvent correction: -226.0718 kJ/mol

[^{Me}**2***-PhNO]$^{-}$

xyz, charge: -1, multiplicity: 1

N	-0.661719	1.226244	0.056591
C	-0.258442	2.429005	0.688219
C	-1.933351	1.098219	-0.044764
C	-1.601530	3.070032	0.955827
H	-1.746522	4.035425	1.418688
C	-2.592201	2.266543	0.541605
H	-3.652263	2.440773	0.629845
N	0.467535	-0.413880	1.670061
C	0.359117	-1.567298	2.379475
C	0.156610	0.637017	2.490038
C	-0.038719	-1.264610	3.667157
C	-0.162133	0.146148	3.737656
H	-0.411494	0.721982	4.616965
N	1.454187	-1.869964	-0.416107
C	2.550691	-1.959284	-1.227915
C	1.582556	-2.806418	0.579889
C	3.353750	-2.986054	-0.790887
H	4.291455	-3.299200	-1.223308
C	2.734578	-3.526161	0.362815
H	3.115147	-4.330867	0.972694
N	1.558623	0.876436	-1.191354
C	1.677268	2.239361	-0.985517
C	2.667258	0.455469	-1.869153
C	2.873475	2.658889	-1.521025
H	3.260312	3.667219	-1.518759
C	3.503588	1.525125	-2.089857
H	4.454260	1.508888	-2.600151
H	1.834417	4.451223	0.812030
C	1.105446	4.510187	0.010721
C	-0.350277	3.456624	-1.647688
C	0.573456	3.148704	-0.447098

H	0.254815	3.908870	-2.433530
H	0.281043	5.141652	0.350827
H	-1.149713	4.158691	-1.396143
H	2.468409	3.047923	1.790517
C	2.045385	2.065094	1.986105
C	0.111145	3.041257	3.163886
C	0.503120	2.051142	2.063663
H	0.702990	2.850757	4.059571
H	2.413865	1.741052	2.960959
H	-0.939944	2.956522	3.443026
H	-1.439149	-3.615986	2.061943
C	-0.737449	-3.506824	1.231533
C	1.155822	-3.877902	2.781546
C	0.598408	-2.918326	1.727023
H	2.100226	-3.517725	3.188970
H	-0.575465	-4.488865	0.780688
H	1.317646	-4.870593	2.357125
C	2.770634	-0.976866	-2.355474
C	4.150022	-1.204153	-2.970381
C	1.719621	-1.202087	-3.462218
H	4.313380	-0.521005	-3.805039
H	1.877445	-0.502171	-4.285992
H	4.940769	-1.044841	-2.237256
H	0.701367	-1.060876	-3.100320
H	4.236488	-2.222543	-3.351758
H	1.792015	-2.220851	-3.849042
H	-0.794858	2.558569	-2.071541
H	1.578019	5.030265	-0.823065
H	0.307325	4.071209	2.857640
H	2.451159	1.378363	1.250174
H	0.449831	-3.987214	3.605576
H	-1.200973	-2.867616	0.484594
H	-0.214955	-1.962816	4.471612
Al	0.386601	-0.326481	-0.244571
N	-2.325164	-0.053629	-0.616404
O	-1.294910	-0.897569	-0.901718
C	-3.608111	-0.487055	-0.896275
C	-4.646539	0.417598	-1.134445
C	-3.872655	-1.856789	-0.975998
C	-5.930275	-0.041729	-1.377168
C	-5.158319	-2.296867	-1.233437
C	-6.201390	-1.400618	-1.424386
H	-4.450742	1.480013	-1.172544
H	-3.070716	-2.564358	-0.829693
H	-6.720003	0.676444	-1.558384
H	-5.345356	-3.362080	-1.281442
H	-7.203845	-1.755331	-1.623209

Total correction:	1840.5653 kJ/mol
Final single point energy:	-5007688.5135 kJ/mol
Final Gibbs free energy:	-5006085.4727 kJ/mol
Gibbs free energy solvent correction:	-220.6616 kJ/mol

[iso-^{Me}**1***-PhNO]$^{-}$

xyz, charge: -1, multiplicity: 1

N	0.297102	-1.656105	-0.083343
C	-0.065260	-2.932298	-0.357502
C	1.448415	-1.666227	0.618934

C	0.928979	-3.774108	0.096356
H	0.937609	-4.852566	0.044425
C	1.916169	-2.964875	0.733075
H	2.772223	-3.318384	1.283596
N	-2.201843	-1.114396	0.397277
C	-3.245387	-0.738781	1.195117
C	-2.423905	-2.412707	0.005836
C	-4.146013	-1.773046	1.293652
C	-3.621148	-2.841759	0.526876
H	-4.069869	-3.813362	0.387069
N	-1.767984	1.554938	0.106170
C	-1.687425	2.741007	-0.568162
C	-2.771685	1.689812	1.034256
C	-2.592835	3.638834	-0.057639
H	-2.771860	4.645349	-0.403651
C	-3.286390	2.964085	0.969323
H	-4.085371	3.366255	1.571764
N	0.507822	0.849697	-1.679776
C	1.884686	0.559582	-1.634779
C	0.418584	2.164342	-1.666374
C	2.604495	1.786901	-1.419745
H	3.671937	1.885411	-1.307258
C	1.697241	2.789217	-1.494979
H	1.884549	3.848184	-1.430163
H	4.359119	-0.821088	-2.988236
C	3.930111	-0.820501	-1.982233
C	1.703508	-1.690269	-2.715851
C	2.454134	-0.599061	-2.051352
H	1.731650	-2.604755	-2.116501
H	4.115502	-1.808852	-1.558452
H	2.188940	-1.919752	-3.669930
H	-2.648733	-2.859955	-2.670176
C	-1.623388	-2.688981	-2.336984
C	-1.764384	-4.665168	-0.847756
C	-1.457155	-3.169756	-0.883606
H	-1.711821	-5.050986	0.169721
H	-1.429743	-1.620154	-2.442965
H	-1.049020	-5.215093	-1.463016
H	-2.542439	-0.373462	3.795287
C	-2.232057	0.444377	3.141906
C	-4.587141	0.874634	2.513773
C	-3.201121	0.567632	1.946179
H	-5.327353	0.990894	1.722118
H	-2.226094	1.371539	3.719620
H	-4.570554	1.789952	3.107280
C	-0.891874	2.868440	-1.835797
C	-1.663064	2.218586	-2.999749
C	-0.663375	4.342441	-2.174616
H	-2.611058	2.738314	-3.142163
H	-1.615524	4.825527	-2.391678
H	-1.093417	2.270079	-3.931353
H	-0.031490	4.448860	-3.058231
H	-1.884472	1.172412	-2.798170
H	-0.203640	4.883873	-1.347932
H	0.669818	-1.431185	-2.904036
H	4.460741	-0.099862	-1.367748
H	-2.761562	-4.869266	-1.240542
H	-0.947308	-3.220697	-3.011241
H	-4.911479	0.069330	3.173523
H	-1.206823	0.248508	2.832431

H	-5.060620	-1.782939	1.866343
Al	-0.652400	-0.017789	0.032535
N	1.761204	-0.421871	1.161894
O	0.711234	0.461463	1.142630
C	2.982832	0.082486	1.462751
C	4.150281	-0.704571	1.402834
C	3.124121	1.432570	1.844884
C	5.385983	-0.157704	1.697823
C	4.370648	1.949784	2.139118
C	5.522190	1.173094	2.070002
H	4.093059	-1.738449	1.099510
H	2.249266	2.061588	1.891986
H	6.261397	-0.793437	1.631529
H	4.441658	2.992674	2.425418
H	6.492268	1.590028	2.305973

Total correction: 1836.6379 kJ/mol
Final single point energy: -5007626.8535 kJ/mol
Final Gibbs free energy: -5006036.7611 kJ/mol
Gibbs free energy solvent correction: -213.2466 kJ/mol

BA (benzaldehyde)

xyz, charge: 0, multiplicity: 1

C	-0.488230	-0.224405	0.000077
C	-0.018964	1.087675	0.000073
C	0.413747	-1.282470	0.000026
C	1.341790	1.331846	0.000023
C	1.777968	-1.037548	-0.000029
C	2.239989	0.269959	-0.000028
H	-0.728382	1.904920	0.000114
H	0.044637	-2.301911	0.000023
H	1.709354	2.349280	0.000025
H	2.478518	-1.861680	-0.000071
H	3.304482	0.464398	-0.000073
C	-1.935762	-0.508722	0.000151
O	-2.797671	0.329130	0.000147
H	-2.188409	-1.588208	0.000193

Total correction: 314.1762 kJ/mol
Final single point energy: -906238.6251 kJ/mol
Final Gibbs free energy: -906020.3713 kJ/mol
Gibbs free energy solvent correction: -25.5103 kJ/mol

[^{Et}**1**]$^{-}$

xyz, charge: -1, multiplicity: 1

N	1.984608	0.105071	-0.069530
C	2.808602	-0.826669	-0.652284
C	2.742124	1.215025	0.207720
C	4.071226	-0.300678	-0.766918
H	4.934279	-0.787209	-1.195295
C	4.031595	0.999927	-0.208130
H	4.861441	1.684451	-0.128760
N	0.185699	-1.880374	0.174301
C	-0.689090	-2.672018	0.876890
C	1.203723	-2.681446	-0.279191

C	-0.223824	-3.963481	0.878000
C	0.974215	-3.973014	0.124872
H	1.581328	-4.836518	-0.099040
N	-1.819958	-0.096122	0.076314
C	-2.709929	0.770452	-0.508903
C	-2.543090	-1.026640	0.780443
C	-3.987732	0.396230	-0.173786
H	-4.903606	0.862680	-0.502645
C	-3.880731	-0.740000	0.661916
H	-4.697826	-1.274627	1.121527
N	-0.044959	1.892358	-0.286204
C	0.924773	2.814741	0.017167
C	-1.039937	2.545596	-0.971081
C	0.564279	4.037008	-0.489869
H	1.114280	4.960927	-0.405857
C	-0.684237	3.860983	-1.135564
H	-1.252367	4.622160	-1.648209
C	3.096275	3.582055	0.951757
C	1.612884	2.026490	2.284721
C	2.103452	2.410841	0.863342
H	2.469278	1.684456	2.871959
H	2.539218	4.496710	1.166425
H	0.957235	1.150482	2.227404
C	1.673082	-1.844872	-2.589512
C	3.422320	-3.147760	-1.342045
C	2.274775	-2.133528	-1.181212
H	0.959846	-1.017489	-2.501919
H	4.110535	-2.755717	-2.097520
H	-0.871324	-2.250529	3.520186
C	-1.331048	-1.453220	2.928013
C	-2.866545	-3.198617	1.977578
C	-1.868085	-2.085324	1.609179
H	-3.675233	-2.761676	2.568727
H	-2.358747	-3.894598	2.652497
C	-2.248715	1.813112	-1.490495
C	-1.835211	1.116382	-2.822380
C	-3.385922	2.806298	-1.792225
H	-4.230664	2.247958	-2.202491
H	-1.483447	1.891316	-3.510151
H	-3.054511	3.467812	-2.599453
H	-0.960842	0.483699	-2.631319
H	3.535130	3.727257	-0.037919
H	3.019081	-4.070356	-1.767513
H	2.476875	-1.458555	-3.223470
H	-0.508941	-0.769944	2.685038
H	-0.679903	-4.813298	1.362163
Al	0.078642	0.008193	-0.019245
C	0.863802	3.126647	3.021028
H	-0.017446	3.441882	2.462439
H	0.534712	2.777388	4.001060
H	1.484521	4.009954	3.181096
C	4.197560	3.454235	1.999113
H	4.940070	4.242459	1.860874
H	3.807450	3.560614	3.012604
H	4.716870	2.498139	1.950181
C	-3.881773	3.650436	-0.622674
H	-4.241743	3.030976	0.196774
H	-3.096341	4.288311	-0.223421
H	-4.706101	4.290744	-0.944399
C	-2.897889	0.258267	-3.492731

H	-3.287379	-0.496716	-2.809850
H	-3.741060	0.850282	-3.851250
H	-2.476063	-0.257675	-4.357120
C	-3.479605	-3.975999	0.817887
H	-4.034317	-3.321096	0.149210
H	-2.722116	-4.480416	0.221344
H	-4.169679	-4.733349	1.196188
C	-2.342760	-0.688076	3.769755
H	-3.138570	-1.329815	4.150188
H	-1.849535	-0.240473	4.634440
H	-2.808359	0.112743	3.196008
C	0.973676	-3.010286	-3.272933
H	1.660655	-3.827864	-3.495918
H	0.172577	-3.409655	-2.651532
H	0.536555	-2.687516	-4.219450
C	4.210829	-3.491512	-0.083148
H	4.693801	-2.613604	0.340724
H	3.573443	-3.914312	0.691224
H	4.987366	-4.224207	-0.314464

Total correction:	2192.0566 kJ/mol
Final single point energy:	-4883983.3322 kJ/mol
Final Gibbs free energy:	-4882059.1857 kJ/mol
Gibbs free energy solvent correction:	-203.0612 kJ/mol

[^{Et}**1***-NO] $^{\bullet-}$

xyz, charge: -1, multiplicity: 2

N	-1.673721	-0.678595	-0.294215
C	-2.091839	-1.873876	-0.112127
C	-2.399709	-0.036293	-1.357872
C	-3.207696	-2.171372	-1.015320
H	-3.727153	-3.114015	-1.087801
C	-3.396805	-1.078854	-1.763357
H	-4.108679	-0.952955	-2.565557
N	0.775679	-1.745413	-0.059400
C	2.009503	-2.177022	-0.463808
C	0.046331	-2.860204	0.268632
C	2.054561	-3.554875	-0.412574
C	0.801126	-3.991212	0.059708
H	0.503522	-5.013108	0.237276
N	1.904614	0.746263	0.024110
C	2.249648	1.933530	0.616049
C	3.062558	0.097163	-0.326866
C	3.621212	2.052568	0.611273
H	4.200327	2.866194	1.020747
C	4.136827	0.889460	0.005331
H	5.179733	0.662328	-0.154107
N	-0.700923	1.743832	0.225826
C	-2.028287	2.051368	0.035569
C	-0.144365	2.761415	0.946403
C	-2.314127	3.238495	0.672034
H	-3.266476	3.749067	0.685701
C	-1.115810	3.690407	1.256025
H	-0.980849	4.601547	1.818792
C	-4.307271	1.051141	-0.039600
C	-3.424428	2.175248	-2.065729
C	-3.004981	1.302902	-0.844888

H	-5.049345	0.570186	-0.688024
H	-4.214830	2.848885	-1.720122
H	-2.337240	-1.907985	2.589694
C	-1.316824	-2.085338	2.235670
C	-2.000976	-4.151180	0.923256
C	-1.350660	-2.764014	0.842096
H	-1.322822	-4.822035	1.452797
H	2.936232	-2.126909	-2.959116
C	2.869424	-1.121823	-2.530087
C	4.463589	-1.916954	-0.763094
C	3.086407	-1.255491	-0.993452
H	5.236036	-1.278708	-1.197507
H	4.494511	-2.838942	-1.351464
C	1.302809	2.840972	1.362887
C	1.402857	2.507646	2.877664
C	1.776183	4.302854	1.186309
H	2.440462	2.652389	3.194084
H	0.794729	3.232833	3.428787
H	2.792519	4.395849	1.578974
H	1.162465	4.947514	1.821553
H	-3.899087	1.533662	-2.817228
H	-4.720441	2.038865	0.178500
H	-2.080511	-4.560741	-0.087139
H	-0.871542	-1.095114	2.134893
H	1.849220	-0.786763	-2.716572
H	2.889267	-4.183515	-0.681106
Al	0.163623	0.104955	-0.346436
N	-1.492779	0.138931	-2.517883
O	-0.235225	0.217901	-2.142535
C	-2.354557	3.026418	-2.734642
H	-1.568162	2.424830	-3.179348
H	-2.809555	3.626290	-3.526607
H	-1.895851	3.710643	-2.021499
C	-4.207531	0.295970	1.278529
H	-5.111529	0.459996	1.868267
H	-4.109188	-0.779844	1.146070
H	-3.360345	0.649059	1.865654
C	0.957742	1.102561	3.247832
H	1.063688	0.932876	4.321184
H	-0.092524	0.947362	2.994868
H	1.550528	0.344778	2.734117
C	1.736427	4.814958	-0.244957
H	2.103272	5.842266	-0.299829
H	2.356800	4.200438	-0.896681
H	0.719828	4.798970	-0.636986
C	3.824253	-0.185684	-3.256785
H	3.558535	-0.129339	-4.313964
H	3.775993	0.822593	-2.846672
H	4.862714	-0.517358	-3.201195
C	4.838194	-2.224792	0.681708
H	4.141714	-2.927486	1.137037
H	5.837160	-2.664551	0.728635
H	4.839615	-1.321566	1.289841
C	-0.534699	-2.868594	3.277475
H	-1.024079	-3.806983	3.546187
H	0.466994	-3.103620	2.918241
H	-0.434359	-2.281009	4.190561
C	-3.358468	-4.192604	1.617402
H	-3.820044	-5.172902	1.491773
H	-3.267652	-4.014149	2.689045

H	-4.058153	-3.450577	1.228706

Total correction: 2218.3057 kJ/mol
Final single point energy: -5224768.3638 kJ/mol
Final Gibbs free energy: -5222824.8944 kJ/mol
Gibbs free energy solvent correction: -211.2558 kJ/mol

[^{Et}**1***-ONNO]$^{-}$

xyz, charge: -1, multiplicity: 1

N	-1.609564	-0.676683	0.073553
C	-2.011892	-1.858094	0.354687
C	-2.457372	-0.076593	-0.924985
C	-3.235517	-2.176761	-0.389145
H	-3.777735	-3.107966	-0.342914
C	-3.513362	-1.124055	-1.164412
H	-4.335264	-1.015234	-1.852785
N	0.846377	-1.722472	0.085800
C	2.031546	-2.165611	-0.435990
C	0.154112	-2.827862	0.513584
C	2.077693	-3.542581	-0.362494
C	0.880729	-3.965210	0.247882
H	0.603722	-4.982526	0.477293
N	1.989514	0.760404	0.024621
C	2.388744	1.957968	0.557433
C	3.101266	0.109548	-0.448256
C	3.750085	2.082332	0.395161
H	4.366661	2.904949	0.723414
C	4.200644	0.912264	-0.249997
H	5.220116	0.687899	-0.523775
N	-0.583947	1.752153	0.465620
C	-1.920963	2.057333	0.375388
C	0.034897	2.788386	1.102667
C	-2.151083	3.263594	0.999495
H	-3.096523	3.781358	1.074721
C	-0.907176	3.729134	1.464942
H	-0.725650	4.656583	1.985812
H	-5.016187	0.631060	-0.104240
C	-4.218334	1.107032	0.477729
C	-3.434081	2.144811	-1.643597
C	-2.967609	1.303731	-0.420496
H	-4.589904	2.110665	0.694183
H	-4.182534	2.853030	-1.276269
H	-0.546793	-0.993918	2.412365
C	-0.966675	-1.981816	2.602182
C	-1.795031	-4.094449	1.454061
C	-1.164155	-2.711829	1.248949
H	-1.938307	-1.797052	3.070942
H	-1.055724	-4.745722	1.922149
H	1.655580	-0.853783	-2.709935
C	2.694229	-1.166218	-2.600204
C	4.447175	-1.917029	-0.968068
C	3.053965	-1.257947	-1.086767
H	5.175580	-1.287545	-1.483567
H	2.739566	-2.182670	-3.004274
H	4.428088	-2.851723	-1.536835
C	1.515143	2.870802	1.384894
C	1.756626	2.551486	2.886536
C	1.972272	4.330052	1.154499

H	3.019872	4.419848	1.455390
H	2.823883	2.673908	3.094621
H	1.422409	4.982320	1.839031
H	1.225885	3.299614	3.485156
H	-3.967314	1.495040	-2.347066
H	-1.990982	-4.539680	0.475070
H	2.878568	-4.180322	-0.702747
Al	0.232322	0.122698	-0.177218
N	-1.592697	-0.032294	-2.097197
O	-0.309148	0.283230	-1.924130
N	-2.116051	-0.303383	-3.251998
O	-1.376515	-0.247194	-4.211411
C	-2.374845	2.948323	-2.387841
H	-1.576619	2.347004	-2.813840
H	-2.846930	3.491255	-3.209049
H	-1.912701	3.679099	-1.725436
C	-4.042519	0.386527	1.807634
H	-4.902138	0.583410	2.450677
H	-3.971416	-0.694374	1.700912
H	-3.151829	0.740095	2.326187
C	1.315165	1.163993	3.319916
H	1.827733	0.384162	2.755274
H	1.530976	1.001138	4.377624
H	0.239911	1.037267	3.183049
C	1.808480	4.835212	-0.270490
H	0.762056	4.817619	-0.574659
H	2.167608	5.862717	-0.360634
H	2.370606	4.219902	-0.972259
C	3.563802	-0.239070	-3.436455
H	3.525990	0.783114	-3.061209
H	4.610900	-0.547129	-3.457852
H	3.206709	-0.228263	-4.467461
C	4.947753	-2.191719	0.444514
H	4.284305	-2.870694	0.977928
H	5.940979	-2.645521	0.414486
H	5.015691	-1.272441	1.023970
C	-0.051329	-2.721393	3.563566
H	0.133351	-2.112197	4.448795
H	-0.480035	-3.666894	3.900966
H	0.910046	-2.936875	3.097915
C	-3.060266	-4.113601	2.304888
H	-2.846518	-3.900919	3.352815
H	-3.805911	-3.385429	1.980155
H	-3.529689	-5.097490	2.266795

Total correction: 2251.8986 kJ/mol
Final single point energy: -5565660.1754 kJ/mol
Final Gibbs free energy: -5563689.3612 kJ/mol
Gibbs free energy solvent correction: -218.11 kJ/mol

NO

xyz, charge: 0, multiplicity: 2

N	0.000000	0.000000	0.029884
O	0.000000	0.000000	1.170116

Total correction: 18.9548 kJ/mol
Final single point energy: -340744.7 kJ/mol
Final Gibbs free energy: -340784.3894 kJ/mol

Gibbs free energy solvent correction: 1.6229 kJ/mol

PhNO

xyz, charge: 0, multiplicity: 1

C	0.497090	-0.210843	0.000051
C	0.028282	1.100991	0.000142
C	-0.387005	-1.280525	-0.000060
C	-1.334011	1.328816	0.000122
C	-1.753052	-1.047155	-0.000097
C	-2.223463	0.257065	-0.000002
H	0.729318	1.924759	0.000237
H	0.013978	-2.285857	-0.000118
H	-1.712631	2.342152	0.000206
H	-2.446455	-1.876976	-0.000193
H	-3.289217	0.444198	-0.000018
N	1.878047	-0.573583	0.000097
O	2.651399	0.340189	0.000063

Total correction: 280.7783 kJ/mol
Final single point energy: -948149.4367 kJ/mol
Final Gibbs free energy: -947963.8882 kJ/mol
Gibbs free energy solvent correction: -23.5683 kJ/mol

TS-3

xyz, charge: -1, multiplicity: 1

N	0.432222	-0.345972	-0.660182
C	0.492286	-0.908970	-1.858269
C	1.631651	0.223267	-0.353434
C	1.767373	-0.674578	-2.407276
H	2.125391	-1.011409	-3.368651
C	2.494086	0.028479	-1.441178
H	3.536324	0.307105	-1.487460
N	-1.165869	-2.231856	0.132766
C	-1.547894	-3.306119	0.885582
C	-1.053722	-2.661422	-1.167716
C	-1.687103	-4.412301	0.078349
C	-1.374347	-3.996815	-1.236782
H	-1.386049	-4.612503	-2.123512
N	-2.151188	-0.731328	2.182417
C	-2.997867	0.173835	2.757427
C	-2.401946	-1.945846	2.771636
C	-3.761491	-0.440639	3.721188
H	-4.527467	0.014608	4.330447
C	-3.377861	-1.798640	3.729718
H	-3.783759	-2.574739	4.359485
N	-1.246031	1.590458	0.655613
C	-0.130457	2.390425	0.430909
C	-1.862773	2.110031	1.723146
C	-0.028311	3.349854	1.459294
H	0.724719	4.119230	1.540539
C	-1.148920	3.205932	2.246158
H	-1.429579	3.807354	3.094979
H	-0.982117	1.669264	-1.958421
C	0.002610	2.111758	-2.052486
C	1.870283	3.235412	-0.794802

C	0.629712	2.390926	-0.736162
H	1.602574	4.291598	-0.899074
H	0.627443	1.484389	-2.691401
H	2.474246	2.960655	-1.656910
H	-1.647763	-0.151970	-3.474452
C	-1.883321	-0.838180	-2.656313
C	-0.307534	-2.538101	-3.551056
C	-0.681607	-1.734625	-2.308361
H	0.520438	-3.217638	-3.349180
H	-2.730890	-1.455903	-2.958136
H	-0.017096	-1.872991	-4.367061
H	0.272022	-4.129814	2.735811
C	-0.276679	-3.237715	3.046634
C	-2.443383	-4.423058	2.902829
C	-1.671174	-3.207583	2.385955
H	-3.443833	-4.473010	2.473422
H	-0.379717	-3.253397	4.133975
H	-2.534716	-4.390133	3.989102
C	-3.170980	1.564921	2.213085
C	-4.159313	1.528931	1.030166
C	-3.750084	2.479158	3.293070
H	-4.289902	2.521775	0.591351
H	-3.870952	3.499103	2.922239
H	-3.814357	0.856650	0.246069
H	-3.117822	2.500464	4.180588
H	-5.128950	1.168393	1.375966
H	-4.734513	2.124656	3.597249
H	2.483355	3.134413	0.099250
H	-0.107831	3.074294	-2.564336
H	-1.154998	-3.130494	-3.897242
H	-2.204592	-0.242423	-1.800858
H	-1.914468	-5.343340	2.651521
H	0.323009	-2.365476	2.796203
H	-1.969312	-5.407322	0.385926
Al	-0.755772	-0.467167	0.871369
C	1.859233	0.321630	1.151434
O	0.726493	-0.106857	1.815630
C	3.074807	-0.517495	1.498246
C	4.280523	0.077689	1.839465
C	3.008141	-1.907754	1.443228
C	5.405900	-0.689780	2.117781
C	4.123801	-2.676181	1.725223
C	5.329983	-2.071339	2.063141
H	4.340902	1.159378	1.895568
H	2.076915	-2.386733	1.167055
H	6.337289	-0.205822	2.385788
H	4.054458	-3.755793	1.678424
H	6.200851	-2.675927	2.284069
H	2.095808	1.363352	1.438915

Total correction:	1866.0153 kJ/mol
Final single point energy:	-4965638.4208 kJ/mol
Final Gibbs free energy:	-4964013.5243 kJ/mol
Gibbs free energy solvent correction:	-221.2356 kJ/mol

TS-4

xyz, charge: -1, multiplicity: 1

N	-0.755832	1.284132	0.426763

C	-0.478018	2.397784	1.179062
C	-2.020818	1.239729	0.098006
C	-1.704778	3.104775	1.290853
H	-1.864678	4.015013	1.849942
C	-2.675105	2.387511	0.604781
H	-3.720315	2.634751	0.495829
N	0.555944	-0.316586	1.933479
C	0.567304	-1.458718	2.679819
C	0.405788	0.746662	2.789802
C	0.411789	-1.134545	4.011216
C	0.313601	0.276267	4.080183
H	0.216185	0.867740	4.978395
N	1.425612	-1.836698	-0.154924
C	2.426205	-2.027856	-1.062752
C	1.583839	-2.772509	0.832737
C	3.205603	-3.094829	-0.681330
H	4.074748	-3.476811	-1.194229
C	2.662687	-3.573039	0.533330
H	3.039029	-4.393546	1.124006
N	1.373021	0.804746	-1.256172
C	1.555993	2.164299	-1.080979
C	2.500434	0.321197	-1.809271
C	2.866134	2.498840	-1.449752
H	3.294768	3.489425	-1.438674
C	3.448839	1.340937	-1.950566
H	4.434857	1.246479	-2.376307
H	1.622238	4.616089	0.175659
C	0.864850	4.530930	-0.598476
C	-0.682831	2.975854	-1.801229
C	0.510878	3.106297	-0.918506
H	-0.506612	3.643657	-2.651875
H	-0.021083	5.073714	-0.275133
H	-1.607610	3.307169	-1.327697
H	2.234255	3.423104	1.653128
C	2.024828	2.400545	1.973202
C	0.206121	3.147927	3.455326
C	0.541797	2.186008	2.313476
H	0.919273	3.031698	4.272056
H	2.608874	2.208340	2.874643
H	-0.793519	2.969328	3.850061
H	-1.377552	-3.388498	2.491980
C	-0.714036	-3.347629	1.624907
C	1.243887	-3.810985	3.063323
C	0.677225	-2.826673	2.037927
H	2.228227	-3.502059	3.414826
H	-0.628210	-4.352853	1.205613
H	1.325825	-4.811034	2.634100
C	2.585601	-1.117860	-2.251373
C	3.929203	-1.373444	-2.930822
C	1.469524	-1.382145	-3.282172
H	4.057486	-0.717714	-3.793699
H	1.560411	-0.710325	-4.140219
H	4.761626	-1.214295	-2.245119
H	0.477721	-1.251487	-2.852850
H	3.979665	-2.402539	-3.286943
H	1.542238	-2.410862	-3.639569
H	-0.808174	1.973026	-2.197718
H	1.244284	5.028833	-1.497103
H	0.262807	4.184516	3.115036
H	2.396035	1.722747	1.210196

H	0.580209	-3.882728	3.925737
H	-1.186362	-2.720548	0.872735
H	0.379249	-1.819851	4.844502
Al	0.270711	-0.307032	0.004773
C	-2.402667	-0.023540	-0.637599
O	-1.286539	-0.835163	-0.735616
C	-3.531937	-0.713627	0.107073
C	-3.392544	-1.010465	1.461785
C	-4.701480	-1.082673	-0.540899
C	-4.404942	-1.662616	2.144063
C	-5.722612	-1.733383	0.141395
C	-5.575784	-2.024715	1.487454
H	-2.484736	-0.729822	1.984613
H	-4.813165	-0.865816	-1.597454
H	-4.278561	-1.890840	3.194804
H	-6.627550	-2.016942	-0.381681
H	-6.366021	-2.534630	2.024432
H	-2.787036	0.237900	-1.641133

Total correction:	1866.4183 kJ/mol
Final single point energy:	-4965615.7779 kJ/mol
Final Gibbs free energy:	-4963987.94 kJ/mol
Gibbs free energy solvent correction:	-218.7656 kJ/mol

TS-5

xyz, charge: -1, multiplicity: 1

N	-1.280727	1.136819	-0.026095
C	-1.533041	2.330670	-0.518878
C	-2.278581	0.774130	0.823696
C	-2.765227	2.792228	0.008093
H	-3.249460	3.728189	-0.224610
C	-3.219371	1.825415	0.893831
H	-4.093876	1.854989	1.522953
N	1.254236	1.613661	-0.123586
C	2.505700	1.825123	0.386847
C	0.848797	2.790271	-0.709246
C	2.905837	3.112697	0.113628
C	1.845728	3.728481	-0.590557
H	1.823238	4.742976	-0.958588
N	1.890009	-1.036049	0.073787
C	2.128999	-2.287767	-0.421932
C	3.064394	-0.581347	0.619552
C	3.431255	-2.644940	-0.171049
H	3.915521	-3.566636	-0.454877
C	4.028647	-1.552721	0.497179
H	5.051110	-1.490032	0.835134
N	-0.804144	-1.479603	-0.915182
C	-2.135392	-1.617069	-0.534667
C	-0.275429	-2.722106	-0.863292
C	-2.381557	-2.941962	-0.167486
H	-3.325034	-3.343654	0.167739
C	-1.217435	-3.651211	-0.418713
H	-1.064654	-4.709934	-0.288990
H	-2.216211	0.208253	-2.446421
C	-3.169701	0.189048	-1.930634
C	-4.486587	-0.902717	-0.084989
C	-3.133542	-0.622226	-0.681135
H	-4.419737	-1.162185	0.969927

H	-3.521849	1.210065	-1.769703
H	-4.962834	-1.734380	-0.612660
H	-1.392872	2.358780	-3.314506
C	-0.446134	2.257956	-2.777336
C	-0.810126	4.429667	-1.619150
C	-0.489632	2.953332	-1.404041
H	-0.814911	4.978287	-0.677144
H	0.344362	2.702955	-3.383028
H	-1.787042	4.547206	-2.093050
H	3.096287	0.066289	3.290890
C	2.586700	0.796948	2.659201
C	4.672782	1.143092	1.376739
C	3.192294	0.786386	1.239393
H	5.175064	1.154700	0.409342
H	1.527185	0.548432	2.668016
H	5.182778	0.426339	2.021518
C	1.134260	-2.995986	-1.300413
C	1.296829	-2.494906	-2.750655
C	1.403869	-4.500754	-1.281711
H	0.575208	-2.975422	-3.416472
H	0.695590	-5.032980	-1.919477
H	1.149574	-1.417738	-2.817120
H	1.337502	-4.902980	-0.271200
H	2.303739	-2.714501	-3.108553
H	2.404260	-4.710173	-1.659719
H	-5.133298	-0.033242	-0.187120
H	-3.895124	-0.288342	-2.598145
H	-0.072852	4.892770	-2.274938
H	-0.222262	1.194813	-2.683971
H	4.788186	2.125702	1.836069
H	2.699344	1.786847	3.106769
H	3.838458	3.574452	0.397854
Al	0.258970	-0.050165	0.099494
N	-1.815744	-0.127283	1.793859
O	-0.520485	-0.474234	1.724551
N	-2.647650	-0.575165	2.678442
O	-2.214205	-1.345463	3.511545

Total correction: 1601.7311 kJ/mol
Final single point energy: -4740967.8097 kJ/mol
Final Gibbs free energy: -4739590.6595 kJ/mol
Gibbs free energy solvent correction: -226.1722 kJ/mol

TS-6

xyz, charge: -1, multiplicity: 1

N	-0.696332	1.239503	0.321037
C	-0.471457	2.323591	1.133530
C	-1.932238	1.219469	-0.086247
C	-1.716857	3.009334	1.201390
H	-1.921996	3.893509	1.786170
C	-2.642642	2.331847	0.421386
H	-3.673170	2.583241	0.233401
N	0.541558	-0.417100	1.797833
C	0.548389	-1.596342	2.484846
C	0.363086	0.599575	2.706305
C	0.377774	-1.342637	3.827932
C	0.263906	0.061285	3.968091
H	0.145791	0.603673	4.894149

N	1.458026	-1.837849	-0.351710
C	2.497247	-1.984392	-1.227644
C	1.564347	-2.833748	0.585632
C	3.242148	-3.085587	-0.880659
H	4.128027	-3.448434	-1.378153
C	2.642051	-3.630278	0.278195
H	2.984963	-4.489988	0.832003
N	1.490014	0.873476	-1.278772
C	1.673209	2.218962	-1.005512
C	2.641467	0.412996	-1.805907
C	3.001023	2.560122	-1.288931
H	3.436429	3.542952	-1.191761
C	3.600814	1.429452	-1.833556
H	4.605745	1.353043	-2.216135
H	1.700980	4.605428	0.381931
C	0.975788	4.564541	-0.425219
C	-0.513307	3.094338	-1.794338
C	0.624242	3.160650	-0.830421
H	-0.251760	3.775562	-2.611180
H	0.081778	5.100514	-0.112208
H	-1.457665	3.448160	-1.381326
H	2.182405	3.367542	1.807493
C	1.979884	2.328047	2.068881
C	0.065664	2.959102	3.483758
C	0.485494	2.065433	2.314935
H	0.740614	2.821295	4.328941
H	2.515263	2.106175	2.993082
H	-0.946436	2.735918	3.820569
H	-1.442540	-3.452370	2.169730
C	-0.773048	-3.375054	1.310263
C	1.146183	-3.987141	2.743289
C	0.630186	-2.927466	1.767533
H	2.132376	-3.729571	3.128893
H	-0.715016	-4.352086	0.825246
H	1.205673	-4.962241	2.257076
C	2.733973	-0.995664	-2.339018
C	4.113063	-1.223165	-2.955304
C	1.679655	-1.169171	-3.451202
H	4.293941	-0.513760	-3.764621
H	1.840880	-0.447735	-4.256230
H	4.906707	-1.115390	-2.215683
H	0.664574	-1.039792	-3.079997
H	4.177398	-2.226544	-3.376117
H	1.753344	-2.175001	-3.867612
H	-0.645241	2.111491	-2.235627
H	1.394665	5.103088	-1.281728
H	0.111978	4.013040	3.199589
H	2.407690	1.691214	1.300165
H	0.464099	-4.087423	3.587992
H	-1.226679	-2.688612	0.597819
H	0.332759	-2.069514	4.624295
Al	0.362060	-0.298069	-0.125650
N	-2.209323	0.127696	-0.890384
O	-1.214116	-0.764291	-1.015910
N	-3.387244	0.006738	-1.430009
O	-3.582880	-0.988002	-2.092633

Total correction:	1601.7412 kJ/mol
Final single point energy:	-4740948.2147 kJ/mol
Final Gibbs free energy:	-4739570.0598 kJ/mol

Gibbs free energy solvent correction: -220.9468 kJ/mol

TS-1

xyz, charge: -1, multiplicity: 1

N	1.151093	0.256028	-1.115060
C	1.680760	0.074962	-2.300238
C	2.059629	0.842074	-0.267949
C	3.009661	0.571133	-2.291722
H	3.692025	0.591386	-3.127496
C	3.267785	1.021065	-1.012418
H	4.203707	1.418916	-0.660100
N	-0.294282	-1.870667	-1.444618
C	-0.705782	-3.146337	-1.171015
C	0.300218	-1.891125	-2.682405
C	-0.393906	-3.969255	-2.229080
C	0.247585	-3.164059	-3.197912
H	0.627469	-3.489740	-4.154272
N	-2.040253	-1.143927	0.526595
C	-3.141388	-0.502308	1.022474
C	-2.263730	-2.492455	0.649115
C	-4.046772	-1.426057	1.485785
H	-5.013925	-1.222747	1.919006
C	-3.483340	-2.699706	1.248088
H	-3.933669	-3.652087	1.480160
N	-1.056465	1.535436	0.031061
C	-0.067737	2.456016	0.351914
C	-2.052982	1.730145	0.928712
C	-0.437458	3.158093	1.496320
H	0.128027	3.943350	1.973050
C	-1.713790	2.724466	1.840879
H	-2.319797	3.086905	2.655157
H	-0.048585	2.408071	-2.283037
C	0.825580	2.945653	-1.934206
C	2.043157	3.758893	0.097598
C	1.059118	2.765806	-0.469683
H	1.596681	4.758636	0.088983
H	1.689695	2.670242	-2.541951
H	2.944646	3.800528	-0.511525
H	0.081864	1.142782	-4.334438
C	-0.285912	0.232684	-3.853589
C	1.753577	-1.020115	-4.524019
C	0.866606	-0.648724	-3.338674
H	2.576388	-1.666556	-4.218833
H	-0.871978	-0.329337	-4.582179
H	2.170532	-0.124327	-4.989367
H	0.586337	-4.430502	0.869093
C	-0.121945	-3.667341	1.199691
C	-1.963101	-4.883070	0.076796
C	-1.270252	-3.522942	0.177887
H	-2.795082	-4.857312	-0.626897
H	-0.522026	-3.959577	2.173132
H	-2.347540	-5.190113	1.050239
C	-3.349225	0.976922	0.843228
C	-3.969947	1.234313	-0.545845
C	-4.324129	1.495883	1.900512
H	-4.121166	2.303513	-0.715452
H	-4.494576	2.567367	1.780445
H	-3.330698	0.853411	-1.341510

H	-3.953010	1.312753	2.908899
H	-4.933499	0.728105	-0.621686
H	-5.289604	0.999346	1.805666
H	2.324123	3.527122	1.121480
H	0.645752	4.011799	-2.107490
H	1.175609	-1.544161	-5.285026
H	-0.963341	0.523562	-3.050370
H	-1.258651	-5.650387	-0.246390
H	0.431283	-2.740860	1.339035
H	-0.582870	-5.028905	-2.301369
Al	-0.431169	-0.391243	-0.178810
N	1.826278	0.465783	1.054495
O	0.716336	-0.314957	1.240039
C	2.525446	0.799952	2.173194
C	3.701551	1.571723	2.139369
C	2.083842	0.345656	3.431590
C	4.391536	1.865934	3.302666
C	2.791954	0.652227	4.576408
C	3.954815	1.413030	4.537700
H	4.081050	1.965737	1.210945
H	1.179572	-0.237955	3.494369
H	5.291343	2.465720	3.232089
H	2.418353	0.288032	5.526002
H	4.500078	1.646647	5.442297

Total correction: 1833.4578 kJ/mol
Final single point energy: -5007593.3123 kJ/mol
Final Gibbs free energy: -5005999.6888 kJ/mol
Gibbs free energy solvent correction: -214.2944 kJ/mol

TS-2

xyz, charge: -1, multiplicity: 1

N	-0.753499	1.162978	0.541276
C	-0.488484	2.272005	1.306143
C	-2.022679	1.100912	0.215659
C	-1.732003	2.936473	1.455557
H	-1.912415	3.825606	2.040887
C	-2.703226	2.223548	0.767431
H	-3.751316	2.462541	0.703309
N	0.680717	-0.404387	1.973137
C	0.803274	-1.555717	2.695163
C	0.551737	0.637328	2.859074
C	0.761485	-1.258195	4.039672
C	0.604452	0.144500	4.142898
H	0.558745	0.716784	5.057363
N	1.412514	-1.883909	-0.209188
C	2.347734	-2.055971	-1.190768
C	1.627421	-2.851573	0.738390
C	3.130448	-3.147175	-0.899130
H	3.957787	-3.523231	-1.480811
C	2.666174	-3.658047	0.334771
H	3.071609	-4.501014	0.871769
N	1.302505	0.790501	-1.230794
C	1.489517	2.146985	-1.024783
C	2.404793	0.324314	-1.847503
C	2.783369	2.491085	-1.440232
H	3.213542	3.480883	-1.420702
C	3.345372	1.348101	-1.998635

H	4.311322	1.269195	-2.471176
H	1.636076	4.587541	0.228103
C	0.820731	4.507660	-0.485106
C	-0.779765	2.966245	-1.631629
C	0.449767	3.085977	-0.797900
H	-0.638024	3.640623	-2.483667
H	-0.039156	5.044483	-0.089042
H	-1.684513	3.298134	-1.121829
H	2.160708	3.461885	1.756380
C	2.026345	2.417123	2.042580
C	0.201299	3.001368	3.586994
C	0.570122	2.091653	2.412459
H	0.944736	2.920064	4.380510
H	2.646573	2.232841	2.920989
H	-0.770619	2.741922	4.005622
H	-1.166130	-3.447571	2.681118
C	-0.603451	-3.392433	1.746923
C	1.482345	-3.925470	2.968742
C	0.836176	-2.911689	2.022492
H	2.504666	-3.641408	3.216836
H	-0.589104	-4.383019	1.286204
H	1.497960	-4.919222	2.517964
C	2.457219	-1.098263	-2.347376
C	3.765673	-1.334606	-3.099132
C	1.292615	-1.314116	-3.334551
H	3.858546	-0.643255	-3.938326
H	1.351789	-0.613669	-4.171972
H	4.631293	-1.210576	-2.448044
H	0.322855	-1.186166	-2.857246
H	3.791114	-2.347234	-3.502017
H	1.335784	-2.330172	-3.730699
H	-0.928780	1.970668	-2.037697
H	1.128528	5.020485	-1.402895
H	0.175809	4.046896	3.271151
H	2.417296	1.790984	1.245655
H	0.912412	-3.999169	3.895478
H	-1.140923	-2.724874	1.076303
H	0.824209	-1.956907	4.859840
Al	0.304974	-0.351983	0.073616
N	-2.335831	-0.003379	-0.534429
O	-1.312293	-0.905000	-0.629074
C	-3.481485	-0.292345	-1.221050
C	-4.489883	0.662336	-1.427572
C	-3.662985	-1.575522	-1.763467
C	-5.642953	0.328294	-2.115499
C	-4.822401	-1.883301	-2.449419
C	-5.831368	-0.945776	-2.630974
H	-4.363649	1.677606	-1.083989
H	-2.890292	-2.318153	-1.639118
H	-6.400770	1.088750	-2.260688
H	-4.936652	-2.883047	-2.850468
H	-6.735287	-1.199078	-3.168592

Total correction:	1833.3449 kJ/mol
Final single point energy:	-5007583.9573 kJ/mol
Final Gibbs free energy:	-5005990.0412 kJ/mol
Gibbs free energy solvent correction:	-213.7272 kJ/mol

TS-7

xyz, charge: -1, multiplicity: 2

N	-0.189976	-2.340583	0.260228
C	-0.579285	-3.428401	1.013732
C	0.345835	-2.848891	-0.901261
C	-0.291704	-4.587846	0.342488
H	-0.494310	-5.592507	0.678180
C	0.305746	-4.217310	-0.881987
H	0.661001	-4.882730	-1.652938
N	-0.172952	-1.044624	2.644694
C	0.297658	-0.328802	3.716801
C	-0.687115	-2.217254	3.155183
C	0.091049	-1.020483	4.880226
C	-0.544549	-2.228244	4.520354
H	-0.852777	-3.013475	5.192132
N	-0.033203	1.414769	1.288364
C	-0.792026	2.373204	0.660442
C	0.084075	1.804321	2.598504
C	-1.202825	3.313366	1.588770
H	-1.790224	4.194569	1.382294
C	-0.626460	2.956531	2.825103
H	-0.717141	3.485932	3.759983
N	-0.148462	0.092983	-1.059180
C	0.098089	-0.738726	-2.124589
C	-0.741519	1.226902	-1.575652
C	-0.378029	-0.179326	-3.280659
H	-0.322370	-0.602508	-4.271264
C	-0.924957	1.074850	-2.927227
H	-1.347042	1.803623	-3.602409
H	0.066500	-3.054238	-3.619244
C	1.044725	-2.718700	-3.275345
C	2.365736	-1.544522	-1.537574
C	0.939187	-1.964310	-1.951674
H	3.000967	-2.424360	-1.418601
H	1.687328	-3.593961	-3.172467
H	2.374576	-1.005600	-0.588841
H	-2.955160	-2.172017	1.367823
C	-2.807527	-3.015054	2.035853
C	-1.252855	-4.619147	3.077446
C	-1.314406	-3.292749	2.312340
H	-0.226898	-4.887064	3.326620
H	-3.259784	-3.883260	1.551862
H	-1.689230	-5.425660	2.487633
H	2.351846	0.320424	1.992900
C	2.355917	0.855340	2.942849
C	1.066439	1.729507	4.873970
C	0.940271	1.010903	3.533325
H	1.722548	1.174950	5.546093
H	2.985511	0.287928	3.630434
H	0.097907	1.834786	5.362528
C	-0.823595	2.528777	-0.830664
C	-2.046191	3.335439	-1.274949
C	0.441974	3.342294	-1.202427
H	-2.022485	3.502186	-2.351896
H	0.482420	3.516616	-2.279200
H	-2.976105	2.825990	-1.032617
H	1.341232	2.798631	-0.914093
H	-2.057766	4.319515	-0.804297

H	0.445337	4.305062	-0.686574
H	2.808695	-0.886671	-2.288500
H	1.483666	-2.082262	-4.045312
H	-1.827726	-4.557926	4.002521
H	-3.341161	-2.811441	2.966053
H	1.493954	2.724113	4.736770
H	2.815766	1.829800	2.765433
H	0.362227	-0.703765	5.875210
Al	-0.220786	-0.474911	0.788584
O	-2.476058	0.137344	1.309248
N	-2.934737	1.090213	0.841452

Total correction:	1571.5903 kJ/mol
Final single point energy:	-4400094.255 kJ/mol
Final Gibbs free energy:	-4398746.0235 kJ/mol
Gibbs free energy solvent correction:	-198.9723 kJ/mol

$[Al(pyrrolato)_4]^-$

xyz, charge: -1, multiplicity: 1

N	1.161032	0.845209	-1.172461
C	0.925809	1.136427	-2.486321
C	2.346188	1.437143	-0.832233
C	1.949119	1.904122	-2.983033
H	2.035744	2.275358	-3.992560
C	2.863638	2.098617	-1.917620
H	3.791304	2.649328	-1.945997
N	1.055809	-0.958022	1.214433
C	0.819402	-1.117123	2.550734
C	2.159326	-1.704520	0.906358
C	1.760403	-1.956328	3.092497
C	2.623075	-2.334897	2.033919
H	3.483429	-2.983551	2.093744
N	-1.167801	-1.154271	-0.906763
C	-2.294904	-0.776874	-1.583273
C	-0.982755	-2.487386	-1.140962
C	-2.824688	-1.857486	-2.242701
H	-3.716512	-1.859219	-2.850370
C	-1.979850	-2.959182	-1.957229
N	-1.080170	1.235253	0.890437
C	-2.232571	0.964854	1.575349
C	-0.812261	2.564722	1.057884
C	-2.696527	2.109128	2.174358
H	-3.589327	2.198399	2.774035
C	-1.782282	3.139342	1.840070
H	-1.834952	4.177004	2.131672
H	1.827695	-2.256141	4.126938
Al	-0.007253	-0.011151	0.006325
H	0.041677	0.765887	-2.982874
H	2.747825	1.330942	0.164077
H	-0.004285	-0.608326	3.028333
H	2.551906	-1.720318	-0.099228
H	-2.654006	0.239945	-1.531639
H	-0.151258	-3.013843	-0.697225
H	-2.095057	-3.975377	-2.301446
H	-2.654400	-0.028891	1.574538
H	0.053107	3.014097	0.594642

Total correction:	833.3375 kJ/mol
Final single point energy:	-2834986.2789 kJ/mol
Final Gibbs free energy:	-2834324.7742 kJ/mol
Gibbs free energy solvent correction:	-188.2791 kJ/mol

[Al(pyrrolato)$_4$-NO]$^{\bullet-}$

xyz, charge: -1, multiplicity: 2

N	1.388721	1.142216	-0.824684
C	1.797568	2.289892	-0.441152
C	1.925211	0.866726	-2.128133
C	2.683444	2.908256	-1.426066
H	3.148182	3.877331	-1.326824
C	2.742093	2.066838	-2.465801
N	-0.846108	1.256727	0.740302
C	-1.556825	2.385829	0.475086
C	-0.234707	1.438350	1.943514
C	-1.401669	3.294823	1.498348
C	-0.550684	2.681831	2.449544
H	-0.231385	3.086884	3.398010
N	-2.067677	-0.884304	-0.740900
C	-2.976939	-1.004716	0.269749
C	-2.512023	-1.665808	-1.768843
C	-3.992700	-1.851724	-0.104265
H	-4.849879	-2.124300	0.492783
C	-3.692030	-2.279228	-1.420971
H	-4.272200	-2.945498	-2.041980
N	0.665115	-1.464169	0.224506
C	1.935925	-1.427591	0.728880
C	2.309709	-2.675557	1.162960
H	3.256295	-2.938621	1.611062
C	1.209597	-3.531376	0.911759
H	-1.861831	4.268934	1.563770
N	0.825040	0.601232	-3.061855
O	-0.186842	0.085779	-2.401444
C	0.230369	-2.753805	0.343160
H	1.458895	2.725045	0.494307
H	-2.141088	2.469333	-0.429276
H	0.380253	0.660454	2.371104
H	-2.841113	-0.472372	1.198084
H	-1.959796	-1.728619	-2.692566
H	2.497960	-0.507391	0.759895
H	1.141414	-4.587626	1.122633
H	-0.758125	-3.032939	0.013107
H	3.276420	2.199299	-3.394340
Al	-0.350805	-0.066363	-0.560527
H	2.565304	-0.029535	-2.052737

Total correction:	859.6302 kJ/mol
Final single point energy:	-3175667.8541 kJ/mol
Final Gibbs free energy:	-3174986.3576 kJ/mol
Gibbs free energy solvent correction:	-210.2548 kJ/mol

[Al(pyrrolato)$_4$-ONNO]$^{-}$

xyz, charge: -1, multiplicity: 1

N	1.408824	-0.161997	1.079563

C	1.504116	-0.427569	2.323300
C	2.598005	0.542954	0.672718
C	2.774017	0.035028	2.888147
H	3.078902	-0.084319	3.916461
C	3.430803	0.665332	1.908689
H	4.393222	1.147818	1.954526
N	-1.464927	1.145082	-1.522429
C	-2.795260	0.840013	-1.555105
C	-1.218277	1.961855	-2.588947
C	-3.394739	1.453871	-2.628394
C	-2.376156	2.177169	-3.296369
H	-2.480077	2.790831	-4.178595
N	-1.315642	-0.078236	1.114707
C	-1.550415	-1.327342	1.605983
C	-1.678512	0.811124	2.078094
C	-2.065356	-1.239617	2.881824
H	-2.374890	-2.065406	3.504516
C	-2.144701	0.140904	3.187396
H	-2.519063	0.588441	4.095369
N	0.259866	-1.246647	-1.354146
C	-0.120486	-1.462571	-2.649784
C	0.971108	-2.349029	-0.963785
C	0.344340	-2.679906	-3.081867
C	1.046212	-3.253374	-1.993815
H	1.540872	-4.212694	-1.965995
H	-4.439109	1.397816	-2.895974
Al	-0.193455	0.315542	-0.377551
N	2.103509	1.788652	0.148368
O	0.962702	1.734994	-0.535727
N	2.725417	2.881855	0.469498
O	2.249599	3.921490	0.070604
H	0.690129	-0.909847	2.855935
H	3.102767	-0.048639	-0.105719
H	-3.233286	0.203880	-0.801734
H	-0.229700	2.352169	-2.769969
H	-1.338608	-2.202092	1.009096
H	-1.587282	1.873920	1.909938
H	-0.706951	-0.729786	-3.181948
H	1.369894	-2.431783	0.034342
H	0.193976	-3.107512	-4.061342

Total correction: 893.4682 kJ/mol
Final single point energy: -3516548.3677 kJ/mol
Final Gibbs free energy: -3515842.4841 kJ/mol
Gibbs free energy solvent correction: -214.2729 kJ/mol

[^{Me}**1***-ON]$^{•−}$

xyz, charge: -1, multiplicity: 2

N	-1.517400	1.007303	-0.450508
C	-1.536973	2.268507	-0.677139
C	-2.244561	0.715441	0.766129
C	-2.412166	2.955680	0.267579
H	-2.605277	4.017159	0.288882
C	-2.826989	2.032897	1.148373
H	-3.441787	2.198877	2.019817
N	1.132984	1.509571	-0.240071
C	2.390549	1.687525	0.265572
C	0.792538	2.664717	-0.896077
C	2.851804	2.941728	-0.073867

C	1.826440	3.568680	-0.813428
H	1.853301	4.559218	-1.241556
N	1.649892	-1.155208	0.144486
C	1.798692	-2.447037	-0.293683
C	2.862542	-0.728973	0.619228
C	3.094146	-2.847168	-0.070455
H	3.524140	-3.805692	-0.317221
C	3.772881	-1.751300	0.516159
H	4.809685	-1.724510	0.813688
N	-1.053681	-1.619623	-0.268298
C	-2.374656	-1.679843	0.100914
C	-0.654855	-2.883696	-0.584949
C	-2.812484	-2.980086	0.002829
H	-3.802752	-3.345753	0.234146
C	-1.714059	-3.751705	-0.440593
H	-1.709466	-4.815434	-0.621133
H	-4.903269	-0.968504	-0.671623
C	-4.231904	-0.124311	-0.509402
C	-3.916225	-0.810806	1.866748
C	-3.188268	-0.484785	0.558102
H	-4.534645	0.027777	2.197698
H	-3.760345	0.104896	-1.464567
H	-3.220288	-1.058921	2.663806
H	-0.348142	1.000510	-2.803978
C	-0.536463	2.062535	-2.950740
C	-0.801685	4.312239	-1.907723
C	-0.526596	2.838164	-1.624921
H	-1.788737	4.445383	-2.355524
H	-1.494835	2.163456	-3.466836
H	-0.067659	4.707938	-2.609238
H	2.607198	1.773273	2.984402
C	2.454920	0.767375	2.587279
C	4.540215	0.968395	1.273404
C	3.046050	0.663400	1.164743
H	5.027370	0.253655	1.937786
H	2.949252	0.052301	3.248344
H	4.701758	1.961196	1.695741
C	0.733003	-3.148923	-1.101374
C	0.822215	-2.644658	-2.559070
C	1.000247	-4.653503	-1.109468
H	0.653303	-1.568518	-2.620363
H	1.984275	-4.869506	-1.527067
H	1.811642	-2.845532	-2.974861
H	0.265802	-5.172598	-1.727130
H	0.068932	-3.133518	-3.181163
H	0.957769	-5.065278	-0.101576
H	-4.580501	-1.662204	1.716965
H	-4.839286	0.733855	-0.208538
H	-0.747208	4.914259	-1.000702
H	0.248308	2.453442	-3.598496
H	5.031935	0.923958	0.301445
H	1.386400	0.562753	2.633299
H	3.806237	3.371601	0.186635
Al	0.082977	-0.104970	0.125760
O	-1.188600	0.391984	2.873176
N	-1.054257	0.371252	1.621476

Total correction: 1574.2768 kJ/mol
Final single point energy: -4400109.7839 kJ/mol
Final Gibbs free energy: -4398755.6839 kJ/mol

Gibbs free energy solvent correction: -217.005 kJ/mol

15 References

1. G. R. Fulmer, A. J. M. Miller, N. H. Sherden, H. E. Gottlieb, A. Nudelman, B. M. Stoltz, J. E. Bercaw and K. I. Goldberg, *Organometallics*, 2010, **29**, 2176-2179.
2. A. Baeyer, *Ber. Dtsch. Chem. Ges.*, 2006, **19**, 2184-2185.
3. S. Depraetere, M. Smet and W. Dehaen, *Angew. Chem. Int. Ed.*, 1999, **38**, 3359-3361.
4. F. Ebner, H. Wadepohl and L. Greb, *J. Am. Chem. Soc.*, 2019, **141**, 18009-18012.
5. F. Ebner, P. Mainik and L. Greb, *Chem. Eur. J.*, 2021, **27**, 5120-5124.
6. Bruker, *SAINT, V8.40A*, Bruker AXS Inc., Madison, Winsconsin, USA.
7. L. Krause, R. Herbst-Irmer, G. M. Sheldrick and D. Stalke, *J. Appl. Crystallogr.*, 2015, **48**, 3-10.
8. G. M. Sheldrick, *Acta Crystallogr. A: Found. Adv.*, 2015, **71**, 3-8.
9. G. M. Sheldrick, *Acta Crystallogr. C: Struct. Chem.*, 2015, **71**, 3-8.
10. C. R. Groom, I. J. Bruno, M. P. Lightfoot and S. C. Ward, *Acta Crystallogr. B: Struct. Sci. Cryst. Eng. Mater.*, 2016, **72**, 171-179.
11. D. Kratzert, *FinalCif, V130*, https://dkratzert.de/finalcif.html.
12. F. Neese, *WIREs Comput. Mol. Sci.*, 2022, **12**, e1606.
13. G. A. Andrienko, Chemcraft 1.8, https://www.chemcraftprog.com, (accessed January 31, 2024).
14. P. Pracht, F. Bohle and S. Grimme, *Phys. Chem. Chem. Phys.*, 2020, **22**, 7169-7192.
15. C. Bannwarth, E. Caldeweyher, S. Ehlert, A. Hansen, P. Pracht, J. Seibert, S. Spicher and S. Grimme, *WIREs Comput. Mol. Sci.*, 2021, **11**, e1493.
16. S. Grimme, J. G. Brandenburg, C. Bannwarth and A. Hansen, *J. Chem. Phys.*, 2015, **143**, 054107.
17. S. Kozuch and J. M. L. Martin, *Phys. Chem. Chem. Phys.*, 2011, **13**, 20104-20107.
18. S. Kozuch and J. M. L. Martin, *J. Comput. Chem.*, 2013, **34**, 2327-2344.
19. S. Grimme, J. Antony, S. Ehrlich and H. Krieg, *J. Chem. Phys.*, 2010, **132**.
20. S. Grimme, S. Ehrlich and L. Goerigk, *J. Comput. Chem.*, 2011, **32**, 1456-1465.
21. F. Weigend and R. Ahlrichs, *Phys. Chem. Chem. Phys.*, 2005, **7**, 3297-3305.
22. S. Grimme, *Chem. Eur. J.*, 2012, **18**, 9955-9964.
23. A. Klamt, *J. Phys. Chem.*, 1995, **99**, 2224-2235.
24. A. Klamt, V. Jonas, T. Bürger and J. C. W. Lohrenz, *J. Phys. Chem. A*, 1998, **102**, 5074-5085.
25. A. Klamt, *COSMO-RS From Quantum Chemistry to Fluid Phase Thermodynamics and Drug Design*, Elsevier Science Amsterdam, 2005.
26. C. C. Pye, T. Ziegler, E. van Lenthe and J. N. Louwen, *Can. J. Chem.*, 2009, **87**, 790-797.
27. E. J. Z. Baerends, T.; Atkins, A. J.; Autschbach, J.; Bashford, D.; Baseggio, O.; Brces, A.; Bickelhaupt, F. M.; Bo, C.; Boerritger, P. M.; Cavallo, L.; Daul, C.; Chong, D. P.; Chulhai, D. V.; Deng, L.; Dickson, R. M.; Dieterich, J. M.; Ellis, D. E.; van Faassen, M.; Ghysels, A.; Giammona, A.; van Gisbergen, S. J. A.; Goez, A.; Gtz, A. W.; Gusarov, S.; Harris, F. E.; van den Hoek, P.; Hu, Z.; Jacob, C. R.; Jacobsen, H.; Jensen, L.; Joubert, L.; Kaminski, J. W.; van Kessel, G.; Knig, C.; Kootstra, F.; Kovalenko, A.; Krykunov, M.; van Lenthe, E.; McCormack, D. A.; Michalak, A. M., M.; Morton, S. M.; Neugebauer, J.; Nicu, V. P.; Noodleman, L.; Osinga, V. P.; Patchkovskii, S.; Pavanello, M.; Peeples, C. A.; Philipsen, P. H. T.; Post, D.; Pye, C. C.; Ramanantoanina, H.; Ramos, P.; Ravenek, W.; Rodrguez, J. I.; Ros, P.; Rger, R.; Schipper, P. R. T.; Schlns, D.; van Schoot, H.; Schreckenbach, G.; Seldenthuis, J. S.; Seth, M.; Snijders, J. G., SCM, Theoretical Chemistry, Vrije Universiteit, Amsterdam, The Netherlands, https://www.scm.com.
28. E. D. Glendening, C. R. Landis and F. Weinhold, *J. Comput. Chem.*, 2013, **34**, 1429-1437.
